# Understanding and quantifying the impact of solute–solvent van der Waals interactions on the selectivity of asymmetric catalytic transformations†

**DOI:** 10.1039/d4sc04329d

**Published:** 2024-12-26

**Authors:** Riya Kayal, Lorenzo Baldinelli, Ingolf Harden, Frank Neese, Giovanni Bistoni

**Affiliations:** a Max-Planck-Institut für Kohlenforschung Kaiser-Wilhelm Platz 1 45470 Mülheim an der Ruhr Germany ingolf.harden@mpg.kofo.de; b Department of Chemistry, Biology and Biotechnology, University of Perugia Via Elce di Sotto, 8 06123 Perugia Italy giovanni.bistoni@unipg.it

## Abstract

The majority of enantioselective organocatalytic reactions occur in apolar or weakly polar organic solvents. Nevertheless, the influence of solute–solvent van der Waals forces on the relative kinetics of competitive pathways remains poorly understood. In this study, we provide a first insight into the nature and strength of these interactions at the transition state level using advanced computational tools, shedding light into their influence on the selectivity. In addition, we introduce a series of computational tools tailored for detailed exploration of the role of the organic solvent across diverse research disciplines. As a case study, we selected a highly relevant asymmetric organocatalytic transformation catalyzed by a chiral Brønsted acid. Our analysis reveals that strong dispersion interactions exist between the transition state and the solvent, predominantly involving specific groups of the catalyst rather than being uniformly distributed around the solute. Short-range repulsion between the transition state and the solvent often counteracts the effect of these dispersion forces on the transition state energy, resulting in a minimal overall influence of solute–solvent van der Waals forces on enantioselectivity. However, for certain geometric configurations of the transition states, the effect these interactions remains significant, favoring specific reaction channels. These results suggest that integrating solvent structural and electronic information into catalyst design strategies could offer new avenues for tuning selectivity of organocatalytic processes.

## Abstract 2

Advanced computational tools are used to investigate the nature and strength of van der Waals interactions between solute and solvent molecules at the transition state level, highlighting their role in selectivity.

## Graphical abstract

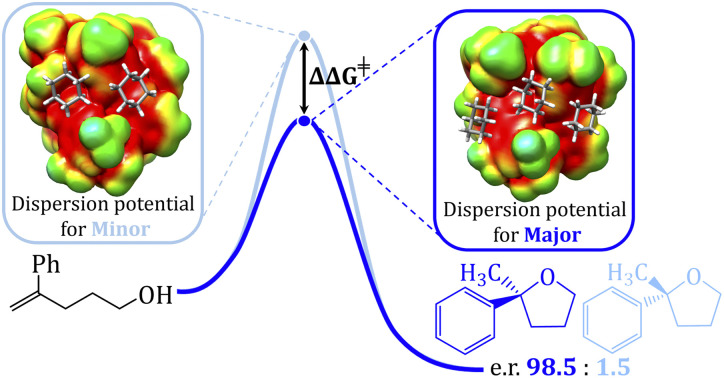

## Introduction

One of the fundamental challenges of synthetic chemistry is the design of environmentally benign and cost-effective chemical processes for the selective synthesis of a specific enantiomer. Chiral catalysts are the tools for achieving that goal. The design of enantioselective catalysts with broad scope is therefore the primary focus area of research in many branches of chemistry.^1,2^

While the activity of a catalyst depends on a number of factors that are contingent upon the nature of the transformation, the enantioselectivity of a chemical process is commonly attributed to one of two main factors. In the prevailing scenario, the selectivity is dictated by short-range steric effects in the transition states,^3–6^ blocking undesired reaction pathways. Alternatively, attractive noncovalent interactions such as hydrogen bonds or London dispersion forces can be used to favor the desired reaction pathway.^7–10^

Clearly, the choice of the appropriate solvent plays a critical role in this context. Unfortunately, unambiguous information into the chemical interactions between solvent molecules and the competing transition states, and their influence on reaction rate and selectivity, are frequently overlooked in the design of new synthetic processes. Such investigations are crucial for enabling a holistic approach for the design of highly selective catalytic transformations.^11^ Since the solvent can influence the reaction outcome in many different ways, *e.g.*, through electronic interactions with the catalyst, reactants or products or by simply dictating the solubility of the different species, *ad hoc* experimental investigations are needed to specifically investigate the role of solute–solvent interactions.^12–15^ Experimental studies on molecular balances have proven instrumental in this context, shedding light into the competition between intramolecular solute–solute and intermolecular solute–solvent interactions for the conformational stability of finely tuned model systems. A much debated yet still unsettled issue in this context concerns the extent of the attenuation of London dispersion forces in solution in the presence of organic solvents. Previous results indicate that the extent of this mitigation is contingent upon solute and solvent nature. In many cases, it has a profound influence in the conformational preferences of molecular balances in organic media.^16–20^

Even though the vast majority of highly relevant organocatalytic transformations occurs in organic solvents,^10,21–25^ an in-depth understanding on the role played by solute–solvent interactions in the differential stabilization of TS conformers is still lacking. This is a highly important task since van der Waals (vdW) solute–solvent interactions might preferentially stabilize one specific TS, thus influencing the selectivity of the transformation. Numerous examples can be found in the literature of diastereo- and/or enantioselective chemical processes whose selectivity is highly influenced by the solvent.^10,16,21–35^ However, the role played by vdW solute–solvent interactions in this context is often overlooked. One of the problems stems from the lack of direct experimental information. While experimental kinetic data are invaluable to deepen our understating of these effects, direct information on the role played by vdW solute–solvent interactions at the TS level can ultimately be obtained only through computational investigations.

Direct solute–solvent interactions are routinely included in the computational modeling of chemical reactions when charged intermediates are considered and/or when polar solvents like water or methanol are used that can engage in strong and directional noncovalent interactions with the catalyst and/or the substrate.^36–43^ In contrast, only a few computational studies specifically address the role played by vdW solute–solvent interactions involving organic solvents, and the role of the solvent is only included in an averaged way using standard implicit solvation schemes The reason for this difference in the treatment of polar *vs.* non-polar solvents is twofold. First, vdW interactions are generally considered to have a smaller influence on reaction energetics compared to stronger, directional noncovalent interactions like hydrogen bonding or dipole–dipole interactions, which dominate in polar solvents. Second, capturing vdW solute–solvent interactions in molecular simulations is challenging due to their non-directional nature. Their impact on reaction mechanisms and outcomes, if present, would depend on the cumulative effect of many weak interactions. Accurately modeling these interactions would require including numerous solvent molecules explicitly in the calculations and properly accounting for dynamic effects, potentially leading to extremely complex and computationally demanding simulations. For these reasons, explicit vdW interactions are often overlooked.

A notable exception was provided by Duarte and Paton, who investigated the solvation of ion-pair structures containing chiral phosphoric acids using both implicit and explicit models.^44^ Another example was discussed by Ping Chin and Krenske, who reported that optimizing the key ion-pairs considering explicit solvation lead to less tightly bound structures, with a potential deep impact on the selectivity of the transformation.^45^

Tkatchenko and coworkers have investigated protein–water interactions in various biological systems using explicit solvation combined with a tight-binding-DFT approach and a many-body dispersion framework.^46^ Their findings demonstrated that solute–solvent van der Waals (vdW) interactions contribute up to 30% of the total solvation energy in their examples, underscoring the crucial role of these interactions in protein stability within aqueous solutions.^47^

Additionally, they explored the influence of static electric fields on intermolecular dispersive interactions, a critical and challenging aspect to model in biological systems, such as ion channels, using existing DFT-based dispersion schemes. Employing a quantum Drude oscillator model, they showed that field-induced vdW interactions can significantly impact the binding energy of (bio)organic dimers and threonine–serine substitution energies in *K*_cv_ ion channels.^48^

To the best of our knowledge, the role of vdW solute–solvent interactions on the differential transition state stabilization of competing pathways in asymmetric transformations in organic media has never been discussed before. One of the reasons for this lack of computational insights stems from the fact that the relative energy between competing TSs leading to different enantiomeric products is often of the order of a few kcal mol^−1^ for highly selective transformations. Hence, the modeling of asymmetric reactions requires the calculation of relative reaction rates with great accuracy (ideally with errors less than 1 kcal mol^−1^). To date, it is not possible to achieve this kind of accuracy directly from *ab initio* molecular dynamic simulations including explicit solvation for asymmetric reactions involving complex catalytic systems.

**Scheme 1:**
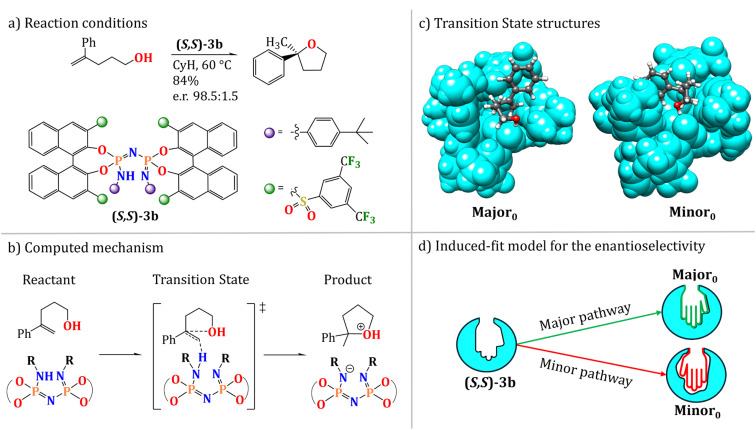
(a) Reaction overview and experimental conditions for the case study investigated in the work, namely the hydroalkoxylation of terminal olefins catalyzed by a bulky IDPi Brønsted acid in cyclohexane (CyH). (b) The proposed mechanism of the transformation. (c) Most stable transition states leading to the major (Major_0_) and minor (Minor_0_) enantiomeric products experimentally detected. (d) Representation of the “induced-fit” model for the interaction of the catalyst and the substrate, which is ultimately responsible for the selectivity. Details can be found in ref. 49.

In the present work, we provide a first insight into the role of vdW interactions in the differential stabilization of TS conformers using a landmark example of organocatalytic transformation as a case study,^10,49^ namely the hydroalkoxylation of terminal olefins catalyzed by a bulky IDPi Brønsted acid in cyclohexane (CyH) (Scheme 1a).

This reaction was selected for this study for two main reasons. First, the catalyst is large and flexible, potentially existing in multiple conformations that can be differentially stabilized by vdW interactions. Second, our group previously reported a thorough computational study of this transformation (ref. 49) using an implicit solvation model. While our results qualitatively reproduced the experimental selectivity trend, they failed to provide a quantitative match, overestimating the enantioselectivity, which suggests a possible role for the explicit solvent.

The preferred pathway for the reaction is shown in Scheme 1b based on our previous mechanistic investigations. In the selectivity-determining step, the catalyst proton is transferred to the terminal methylene group of the folded substrate, leading to simultaneous formation of a C–O bond (Scheme 1c). The catalyst–substrate interaction was described by an induced-fit model, in which both the catalyst active site and the substrate are distorted to optimize their interaction in the TS. The selectivity in this case is linked to the energy required to achieve the optimal geometric match between the catalyst and the substrate in the competing TSs that lead to different enantiomeric products (Scheme 1d).

We note that this selectivity model can be viewed as an extension of the activation strain model (ASM) proposed by Bickelhaupt and Houk.^50^ While ASM is commonly used to discuss activation barriers in terms of the geometrical strain of the reactants in the transition state, our previous work (ref. 49) focused on unraveling the individual energy contributions associated with the relative strain of both the catalyst and substrate in competitive reaction channels – specifically, the relative changes in dispersion interactions originating from individual functional groups. This was achieved through a series of geometry modifications of the catalysts followed by energy decompositions, as discussed in the original manuscript. This approach demonstrated that the disruption of intra-catalyst dispersion forces determines the observed selectivity. However, the influence of vdW solute–solvent interactions, including the potential role of solvent-induced attenuation of London dispersion forces on the relative conformational preferences of the transition states, and their consequent effects on the stereocontrolling factors of this transformation, remains unexplored.

## Results and discussion

The role of vdW solute–solvent interactions in stabilizing the enantio-determining transition states was examined using three complementary computational strategies, each offering insights into different aspects of this complex phenomenon. The scope of each computational strategy is summarized as follows:

(i) An implicit solvation model was used to describe in an approximated way the average effect of solute–solvent vdW interactions on the enantioselectivity as well as the effect of solvent polarity.

(ii) A hybrid implicit/explicit solvation model was used to determine whether strong and directional vdW interactions between the solvent and the catalyst–substrate adduct are present in the transition states, or if the vdW interactions between the solute and solvent are non-directional and homogeneously distributed. Additionally, the model was used to discuss the potential role of these interactions in the selectivity of the transformation.

(iii) An *ad hoc* vdW potential representing the interaction of the solute with the solvent was defined and the main features of this potential were discussed. This tool provides a clear-cut visual representation of the key functional groups in the catalyst that are more likely to engage in strong vdW interactions with solvent, and we believe that it could be useful in future catalyst design strategies.

The basic principles underlying each of these strategies are outlined in the following before discussing the key chemical results. Additional computational details are provided in the ESI.† Unless otherwise specified, calculations were performed at the B3LYP-D3(BJ)/def2-TZVP level with a development version of ORCA based on ORCA 5.0.^51^ It is noted that in the context of this work, “solute” refers to the combined system of catalyst and alkene substrate.

### The average effect of the solvent: results from implicit solvation models

The average effect of the solvent on the relative energy between the transition states in the selectivity-determining step was investigated using different implicit solvation schemes, each of them accounting for a different physical component of the solute–solvent interaction. Cyclohexane (CyH) and acetone (Ac) were used as solvent, as representative examples of organic apolar and polar solvents, respectively. The analysis was performed on ten low-energy conformers initially optimized in the gas phase in ref. 49 (Fig. S1 and S2†). Only conformers with significantly different structural features were selected for this analysis, identified using an RMSD-based criterium.

Implicit solvation corrections were included in three different ways. First, the role of solvent polarity was studied using the conductor-like polarizable continuum model (C-PCM).^52^ Second, the implicit solvation model based on density (SMD)^53^ was used, which accounts for both electrostatic and non-electrostatic effects to the solvation free energy. The latter are included *via* the so-called “Cavity-dispersion solvent structure” (CDS) term, and incorporate approximately the average role of solute–solvent vdW forces. For the sake of comparison, the SMD model was also used without the CDS term (this approach is denoted hereafter as SMD′).

**Fig. 1:**
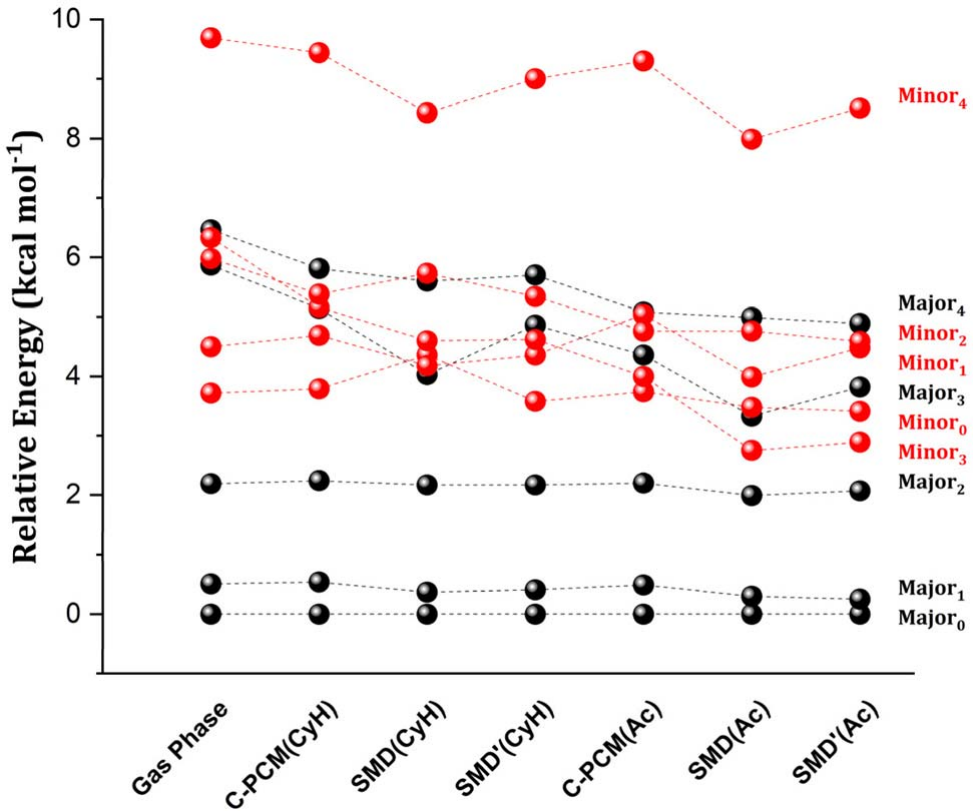
The role of the solvent on the relative stability between different transition state conformers leading to the major (in black) and minor (in red) enantiomeric products. Relative electronic energies at the DFT level (*vide infra*) are calculated for cyclohexane (CyH) and acetone (Ac). Different implicit solvation schemes are tested, namely C-PCM, SMD and SMD without the vdW contribution (SMD′).

In Fig. 1, we report the relative energy between the TS conformers obtained using the settings just described (see Table S1† for the energetics). In the following, the subscript in the conformer name indicates its energy ranking in the gas phase for each enantiomeric pathway (Minor/Major), with 0 representing the lowest energy conformer. In all cases, the energy of the most stable conformer for the TS leading to the major enantiomeric product, Major_0_, was taken as reference.

In the gas phase, the lowest energy conformers correspond to those leading to the major enantiomeric product observed experimentally. Notably, when implicit solvation is included, the relative energy between Major_0_ and the most stable TS leading to the minor enantiomeric product remains largely unchanged, regardless of the solvent model used. In general, a significant solvation effect on the relative energy between TS conformers is seen for the higher energy structures, with many showing energy inversions across different computational settings.

**Scheme 2:**
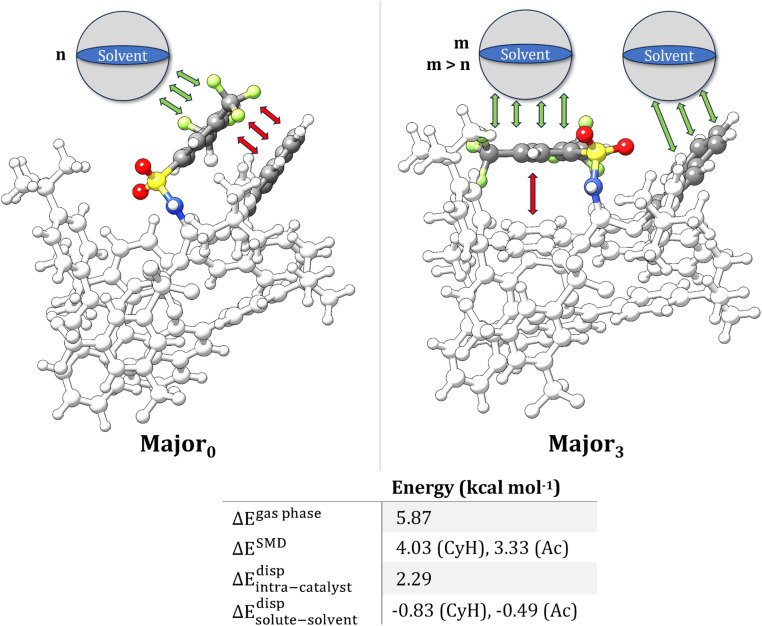
The competition between intra-catalyst (red arrows) and solute–solvent vdW (green arrows) interactions in two different transition state conformers leading to the major enantiomeric product. The key Ph-(CF_3_)_2_ and aryl groups of the catalyst are emphasized. Relative electronic energies are reported in the gas phase (Δ*E*^gas phase^) and using implicit solvent (Δ*E*^SMD^). Relative intra-catalyst dispersion forces (Δ*E*^disp^_intra-catalyst_) are estimated at the B3LYP-D4 level. The differential stability provided by solute–solvent vdW forces (Δ*E*^disp^_solute–solvent_) is estimated approximatively as the difference between the corresponding CDS terms in SMD calculations (*vide infra*).

To gain initial insights into the role of solute–solvent van der Waals (vdW) interactions in the TSs, it is useful to examine the individual contributions of electrostatics and CDS to the SMD solvation. The latter can be estimated from the difference between SMD and SMD′ energies (see Table S2†). This analysis revealed that the differential stabilization of the TS conformers due to the electrostatic contribution can reach up to 2–3 kcal mol^−1^, depending on the computational settings. In contrast, the vdW contribution remains below 1 kcal mol^−1^. Despite the comparatively smaller role of vdW interactions, a correlation analysis between the differential stability provided by solute–solvent vdW interactions and the structural features of the TS revealed some intriguing aspects. Specifically, the aminyl Ph-(CF_3_)_2_ group of the catalyst engages in strong intra-catalyst dispersion interactions in Major_0_ and Minor_0_ as detailed in ref. 49 and in the ESI.† The rotation of the aminyl Ph-(CF3)_2_ group in certain TS conformers disrupts these intra-catalyst London dispersion interactions, raising their energy with respect to Major_0_ in the gas phase. However, the rotation of the aminyl Ph-(CF_3_)_2_ group also correlates with increasing attractive solute–solvent vdW interactions in solution (Scheme 2). This effect likely originates from the increased area that is accessible to the solvent upon the rotation.

While solute–solvent vdW interactions appear to be less significant than intra-catalyst dispersion forces, these results provide initial evidence to the notion that attenuation of intramolecular LD forces in organic solvents is possible at the TS level for organocatalytic transformations in organic solvents, with a potential effect on the selectivity of many transformations.

### The role of directional solute–solvent vdW interactions: results from hybrid implicit/explicit solvation models

Analyses based solely on implicit solvation schemes can only provide information on the average effect of the solvent and are unable to describe the details of the interactions between the solute and solvent at the molecular level. Our goal in this section is to determine whether strong and directional vdW interactions are present in the TSs and their potential effect on the selectivity using a hybrid implicit/explicit approach. For this study we focus on the solvent cyclohexane (CyH), which was the solvent originally selected in the experimental study reported in ref. 10.

In our protocol, we follow an iterative approach in which an increasing number of solvent molecules is included in the simulations, while the remaining of the solvent is described using an implicit solvation scheme. For the pathways leading to each of the enantiomeric products, it is necessary to identify a complete structural ensemble for the system containing both the TS and the solvent molecules in order to identify the preferred sites of the catalyst for the interaction with the solvent. This is far from being a trivial task even with a few solvent molecules due to the large number of degrees of freedom. Hence, to reduce the complexity of the problem, we focused our attention on the most stable TS conformers leading to each enantiomeric product, namely Major_0_ and Minor_0_.

**Fig. 2:**
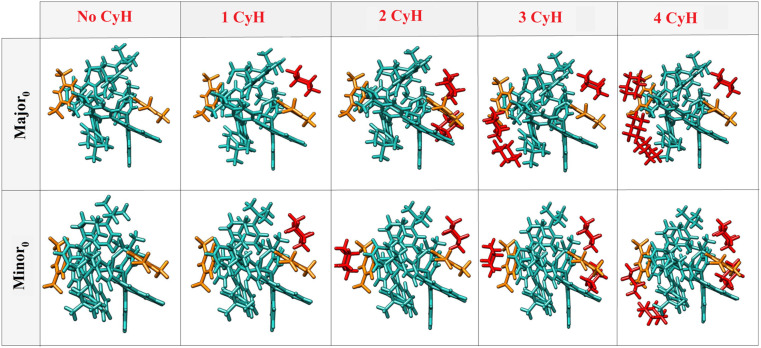
The most stable solvated TS structures obtained for an increasing number of explicit solvent molecules. Solvent molecules are colored in red, the key Ph-(CF_3_)_2_ groups of the catalyst are shown in orange, while the rest of the solute is depicted in aqua green.

Our computational protocol was inspired by Grimme's quantum cluster growth (QCG) approach.^54^ However, the focus in the present case is the description of microsolvation at the TS level and the identification of key differences in solute–solvent interactions for TS conformers leading to different enantiomeric products. In the present case, Major_0_ and Minor_0_ were considered as initial guess structures. In the next step, an initial conformational sampling is performed using the Conformer–Rotamer Ensemble Sampling Tool (CREST)^55–57^ at the *x*TB-GFN-FF^58^ level of theory (see ESI for details†). Importantly, the solute (meaning both, the catalyst and the alkene substrate) was kept frozen at its DFT-optimized TS structure. This allows us to obtain conformers that are lower in energy with respect to those obtained from an unconstrained sampling due to the significant reduction in the number of degrees of freedom. In order to avoid dissociation of the solute–solvent aggregate, an ellipsoid wall potential^56^ was used. Afterwards the whole conformer ensemble was optimized at the GFN2-*x*TB level, again with fixed solute. The most stable conformers with significantly different structural features, identified using a RMSD-based criterium, were subjected to constrained geometry optimizations at the PBE-D3/def2-SVP level of theory.^59^ Afterwards, fully relaxed transition state geometries were obtained using the r2SCAN-3c composite method.^60^ For the next iteration, another solvent molecule was added to the most stable optimized structure from the previous iteration, and the above conformational sampling was reapplied. Ideally, selecting the optimal number of solvent molecules would require a systematic study where solvent molecules are progressively added until solvation energy convergence is reached. However, for the systems studied here, such calculations are computationally prohibitive. Therefore, we repeated this procedure up to the addition of four solvent molecules, at which point we observed qualitative convergence in the key structural features and energetics for the solute–solvent adduct, as detailed in the following. The lowest in energy conformers for the solvated TS complexes Major_0_ and Minor_0_ are shown in Fig. 2.

An important finding of this analysis is that the solvent molecules preferentially cluster around the Ph-(CF_3_)_2_ groups in all structures. Hence, these groups are engaged in strong vdW interactions with both the solvent and the remainder of the catalyst. While these results already suggest that solute–solvent vdW interactions might indeed attenuate the effect of intra-catalyst dispersion forces on the selectivity, it should be mentioned that several different solvated TS conformers of similar energy were identified for a given number of solvent molecules. Hence, it is difficult to draw unambiguous information on the presence of directional vdW interactions based on the analysis of a single solvated structure.

**Fig. 3:**
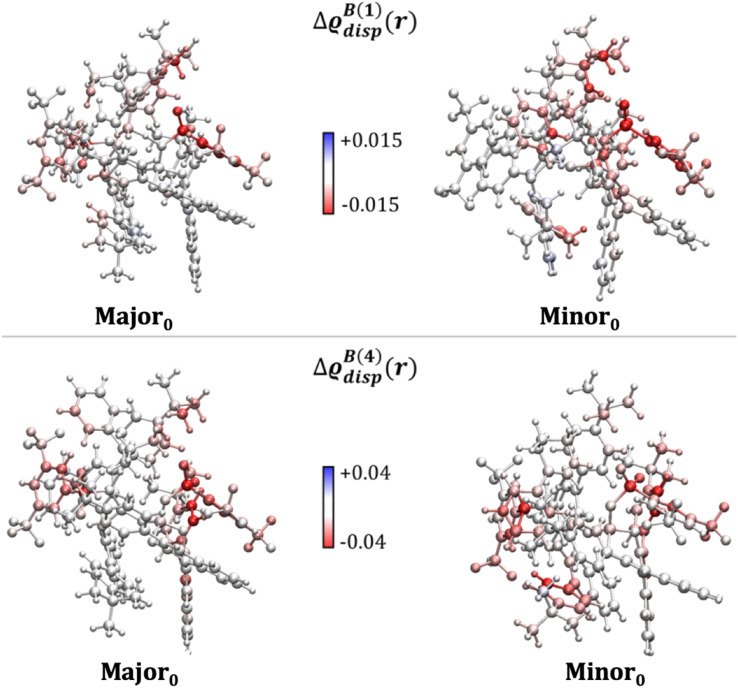
Boltzmann-average of the dispersion difference function Δϱ_disp_^B(*N*)^(*r*) emphasizing the contribution of each atom in the solvated transition states to the dispersion interaction with the explicit solvent (cyclohexane). *N* is the number of solvent molecules. Δϱ_disp_^B(*N*)^(*r*) in kcal mol^−1^ bohr^−3^ was evaluated on the ball-and-stick surfaces.

To corroborate these findings with information from the entire structural ensemble, we developed a computational strategy that is based on a recently introduced computational scheme for the quantification of atomic contributions to the dispersion energy of a chemical system.^61^ In the present case, the approach was used to estimate the contribution of each atom A in the solvated TS to the solute–solvent dispersion energy, Δ*ε*^disp^_A_. This is computed as the difference between the atomic London dispersion contribution of A within the solvated TS and the same quantity computed for the isolated TS. A Boltzmann average of Δ*ε*^disp^_A_ over the entire structural ensemble was performed to get Boltzmann-averaged atomic contributions, Δ*ε*^B,disp^_A_. These contributions are shown graphically in Fig. 3*via* the dispersion difference function, which is defined as:1
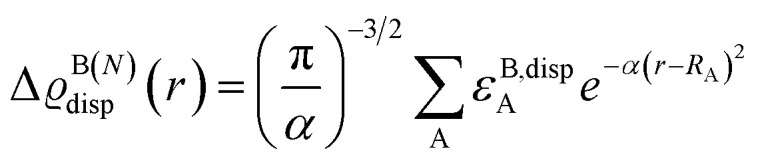
where the superscript *N* denotes the number of explicit solvent molecules considered.

Irrespective of the number of solvent molecules considered, these results clearly show that the atoms contributing most significantly to the solute–solvent dispersion interaction are those belonging to the Ph-(CF_3_)_2_ groups of the catalyst. These findings are derived from an average over the entire structural ensemble and are thus not biased by the selection of a specific structure.

Importantly, integrating Δϱ_disp_^B(*N*)^(*r*) over the entire space provides quantitative information about the tendency of each conformer to engage in dispersion interactions with the solvent. Specifically, the solute–solvent dispersion interaction amounts to −10.2 and −7.4 kcal mol^−1^ for Minor_0_ and Major_0_, respectively, when *N* = 1, and to −27.6 and −25.4 kcal mol^−1^ when *N* = 4. For comparison, the overall difference in energy between Minor_0_ and Major_0_ amounts to 3.4, 3.9 and 2.8 kcal mol^−1^ in the implicit solvent and with *N* = 1 and *N* = 4, respectively (see Table S5† for the energetics obtained at different levels of theory and with an increasing number of solvent molecules). These results demonstrate that the solvent is engaged in strong and directional dispersion interactions with the solute. However, for the most stable TS conformers leading to each enantiomeric product, these interactions are balanced to some extent by the repulsive part of the vdW potential, and hence the overall effect of explicit solvation on the relative energy between the TS conformers is not particularly significant, consistent with the results discussed in the previous section.

### Insights from a molecular dispersion potential (MDP)

Hybrid explicit/implicit solvation models are computationally demanding and hence they cannot be implemented in standard computational workflows. In addition, one could argue that simulations containing a finite number of solvent molecules cannot capture the details of the intricate array of solute–solvent vdW interactions under the experimental conditions. Hence, we decided to develop an approach that is free from both of these limitations and that provide information that are complementary to those obtained in the previous sections. Specifically, we developed a molecular dispersion potential (MDP) providing insights into the inherent tendency of each TS conformer to engage in vdW interactions with the solvent.

The definition of a dispersion potential is not unprecedented,^62^ and different definitions are possible based on different assumptions. In our opinion, the local energy decomposition (LED) is an attractive way to define such potential since it is based on high-accuracy coupled cluster (CC) wavefunctions from which the dispersion energy emerges in a straightforward and natural way.^58,59^ At lower computational cost, one can extract the dispersion energy from the semiclassical dispersion models like the ones proposed by Grimme *et al.*^60^ In the present case, we based our analysis on an expression derived from the D3(BJ) correction,^61^ but the scheme proposed here can be easily generalized to any mean field or post-HF scheme. The expression for the MDP reads:2
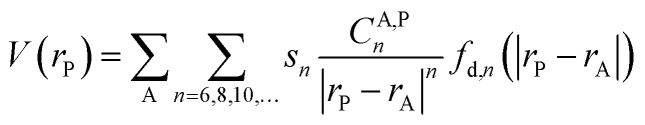
where the first sum runs over all atoms of the system, P indicates the solvent “probe”, *C*^A,P^_*n*_ represents the dispersion coefficient with order *n*, *s*_*n*_ is a scaling factor and *f*_d,*n*_(|*r*_P_ − *r*_A_|) is the damping function. Ideally, *C*^A,P^_*n*_ should be parametrized for each solvent over a large number of experimental data to obtain quantitative information. However, since the main features of the potential are expected to be the same irrespective of the specific nature of the probe, we decided to use the He atom as a probe in the present case. In this way, all the parameters in eqn (2) are defined at the B3LYP-D3(BJ) level, consistent with the other results reported in this manuscript. In addition, since the repulsive contribution of the vdW potential between the solute and the solvent in a given point in space is reasonably related to the value of the solute electron density at that point, we decided to plot the MDP on an isosurface of the one electron density of the solute.

**Fig. 4:**
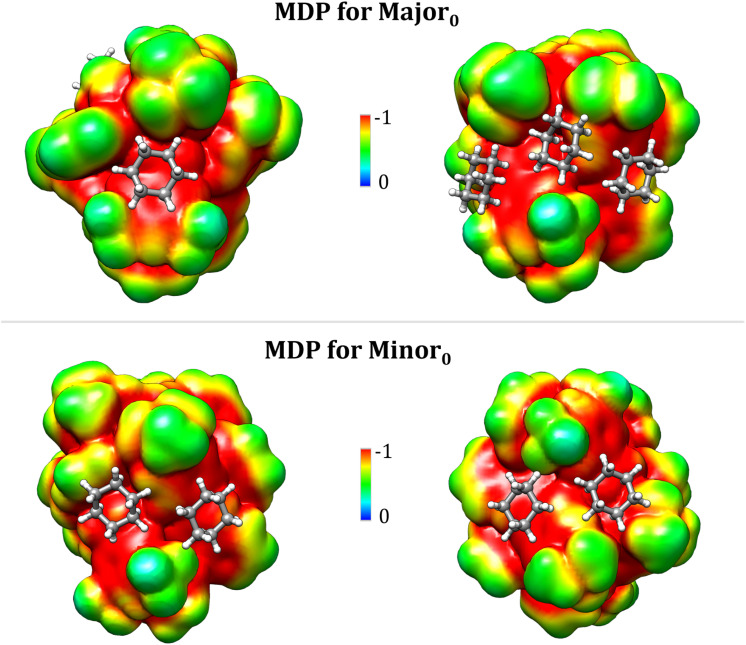
Molecular dispersion potential (MDP) for the Major_0_ (upper panel) and Minor_0_ (lower panel) transition states. Four cyclohexane molecules are superimposed onto the MDP in the positions they occupy in the corresponding most stable solvated conformers (Fig. 2). Notably, all solvent molecules are located in regions where the potential is most attractive. The MDP is plotted on an isosurface of the one electron density (isovalue: 0.0005 a.u.).

The MDP associated with Major_0_ and Minor_0_ are reported in Fig. 4. Importantly, the key sites in the catalyst for the interaction with the organic solvent that were identified in the previous sections are also those where the MDP potential is most attractive. These results confirm the vdW nature of the interaction between solute and the organic solvent in this system and provide an independent verification of the findings reported in the previous sections concerning for example the importance of the Ph-(CF_3_)_2_ groups of the catalyst. Specifically, intense red areas are found close to the Ph-(CF_3_)_2_ groups, indicating that solvent molecules preferentially cluster in these regions, as also demonstrated in the previous section. Indeed, superimposing the MDP with the corresponding most stable solvated transition state conformer obtained from the hybrid implicit/explicit solvation model reveals a nearly perfect alignment between the red regions in the MDP and the positions of the explicit solvent molecules. This suggests that the MDP could serve as a valuable and cost-effective predictive tool for exploring the role of the solvent in various chemical reactions. Additionally, the predictive nature of this approach makes it especially promising for studying vdW interactions not only within the field of catalysis but also across diverse areas of chemical research, including drug design and materials science.

## Conclusions

We investigated the role of van der Waals interactions on the selectivity and mechanism of a landmark example of organocatalytic transformation in an organic solvent. In doing so, we introduced a new set of computational tools that can be used to investigate in detail the role of the organic solvent in this important area of chemical research and also across different research domains.

First, an implicit solvation model was used to estimate the average effect of solute–solvent van der Waals interactions and solvent polarity on enantioselectivity. While this approach offers valuable insights, it lacks a detailed description of explicit vdW solute–solvent interactions, potentially leading to an incomplete representation of the role of the solvent. To address this limitation, we implemented a hybrid implicit/explicit solvation model, enabling us to examine whether strong, directional vdW interactions occur between the solvent and the catalyst–substrate adduct in the transition states. However, achieving convergence in results from hybrid models with respect to the number of solvent molecules is challenging, as quantitative convergence typically requires a large number of solvent molecules, which leads to a steep increase in computational cost. As a cost-effective alternative, we developed an *ad hoc* vdW potential to visually represent the functional groups of the catalyst most likely to engage in strong vdW interactions with the solvent. While this tool provides valuable qualitative insights for catalyst design, it does not allow for precise quantification of solvation energies. Nevertheless, it serves as a useful predictive tool for identifying regions of the catalyst where solvent interactions may exert significant influence.

For the reaction considered in this work, the solvent exhibits strong van der Waals interactions with specific key functional groups in the catalyst. While the net effect of these interactions on the enantioselectivity is negligible for the case study considered here, they have a profound influence on the stability of many high-energy transition states leading to the enantiomeric products. Importantly, solute–solvent dispersion forces have the potential to attenuate the influence of intra-catalyst dispersion forces, potentially impacting the selectivity of many chemical processes.

This attenuation could potentially influence the selectivity of various reactions, particularly for organocatalytic transformations catalyzed by large and flexible catalysts with many structural degrees of freedom. In such cases, competing pathways may exhibit significantly different transition state structural features, and the solute–solvent interactions could vary substantially between the different conformations, potentially impacting the relative energy of the transition states and favoring one pathway over the other, thereby influencing reaction selectivity. Our findings call for further computational and experimental studies to deepen our understanding of solute–solvent van der Waals interactions in asymmetric catalytic transformations.

## Data availability

The raw data used in this manuscript are available in the ESI† and from the corresponding author upon reasonable request. Additional tables and figures are provided in the ESI.†

## Author contributions

RK conducted most of the calculations reported in this work and drafted the original manuscript. LB developed the tools for quantifying the atomic contributions to dispersion energy and generating the molecular dispersion potential, and also contributed to the manuscript writing. FN participated in discussing the results and writing the manuscript. IH contributed to data generation, collection, analysis, and manuscript writing. GB conceived and supervised the project, and finalized the manuscript with input from all authors.

## Conflicts of interest

There are no conflicts to declare.

## Supplementary Material

SC-OLF-D4SC04329D-s001
