# Supplementary material for: Understanding and quantifying the impact of solute–solvent van der Waals interactions on the selectivity of asymmetric catalytic transformations
Source: Chem Sci. 2024 Dec 26;16(6):2700–9. doi: 10.1039/d4sc04329d (PMC11718370; doi:10.1039/d4sc04329d)
Supplement: SC-016-D4SC04329D-s001 [file SC-016-D4SC04329D-s001.pdf]

Supplementary Information (SI) for Chemical Science.
This journal is © The Royal Society of Chemistry 2024

Understanding and Quantifying the Impact of Solute-Solvent Van-der-Waals Interactions on the Selectivity of Asymmetric Catalytic Transformations in Organic Solvents – Supporting Information

Riya Kayal [a], Lorenzo Baldinelli[b], Ingolf Harden *[a], Frank Neese [a], Giovanni Bistoni *[b]

[a]*Max-Planck-Institut für Kohlenforschung, Kaiser-Wilhelm Platz 1, 45470 Mülheim an der Ruhr (Germany)*

[b]*Department of Chemistry, Biology and Biotechnology, University of Perugia Via Elce di Sotto, 8, 06123 Perugia (Italy).*

E-mail: ingolf.harden@mpg.kofo.de
E-mail: giovanni.bistoni@unipg.it

Table of Contents

S1 Effect of implicit solvation models on the relative stability of TS conformers 2

S2 Decomposition of relative electronic energies in geometric preparation and catalyst-substrate interaction contributions 3

S3 3D Structures of selected conformers 4

S4 Hybrid implicit/explicit solvation model: Computational details and number of optimized conformers at each step 5

S5 Effect of hybrid explicit/implicit solvation models on the relative stability of TS conformers........8

S6 Influence of explicit solvation on transition state structures........9

S7 References........10

S8 XYZ Coordinates 11

S1 Effect of implicit solvation models on the relative stability of TS conformers

Table S1. Relative electronic energies (in kcal mol^{-1}) between transition state conformers using different implicit solvation models and solvents at the B3LYP-D3(BJ)/def2-TZVP level of theory. **Major$_0$** was used as reference in all cases. CyH: cyclohexane, Ac: acetone, GP: Gas phase. E(SMD') = E(SMD) – E(CDS).

	GP	CPCM/CyH	CPCM/Ac	SMD/CyH	SMD/Ac	SMD'/CyH	SMD'/Ac
**Major$_0$**	0.00	0.00	0.00	0.00	0.00	0.00	0.00
**Major$_1$**	0.51	0.54	0.49	0.37	0.30	0.41	0.25
**Major$_2$**	2.19	2.24	2.20	2.17	1.99	2.17	2.07
**Major$_3$**	5.87	5.14	4.36	4.03	3.33	4.86	3.82
**Major$_4$**	6.46	5.81	5.08	5.60	4.99	5.70	4.89
**Minor$_0$**	3.72	3.79	3.74	4.36	3.48	3.58	3.41
**Minor$_1$**	4.50	4.69	5.04	4.18	3.99	4.36	4.48
**Minor$_2$**	5.98	5.38	4.76	5.73	4.76	5.34	4.59
**Minor$_3$**	6.33	5.16	4.00	4.60	2.75	4.62	2.89
**Minor$_4$**	9.69	9.44	9.30	8.43	7.99	9.00	8.51

Table S2. Cavity-dispersion solvent structure (CDS) term contribution to the relative energy between TS conformers at the B3LYP-D3(BJ)/def2-TZVP +SMD level (SMD – SMD'). All energies are in kcal mol^{-1}.

	SMD – SMD' (CyH)	SMD – SMD' (Ac)
**Major$_0$**	0.00	0.00
**Major$_1$**	-0.04	0.05
**Major$_2$**	-0.01	-0.08
**Major$_3$**	-0.83	-0.49
**Major$_4$**	-0.10	0.10
**Minor$_0$**	0.77	0.07
**Minor$_1$**	-0.18	-0.48
**Minor$_2$**	0.39	0.17
**Minor$_3$**	-0.02	-0.14
**Minor$_4$**	-0.57	-0.52

S2 Decomposition of relative electronic energies in geometric preparation and catalyst-substrate interaction contributions

The relative electronic energies between the conformers were decomposed into additive contributions corresponding to the catalyst/substrate distortion and catalyst-substrate interaction (Table S3). The results confirm that the lowest in energy TS conformer, **Major0**, features the smallest energetic contribution from the geometric distortion over the entire set of conformers considered in this work. Hence, the design of highly selective catalysts should consider the structural features of the substrate and of the chiral pocket of the catalyst.

Table S3. Relative electronic energies $\mathbf{\Delta E_{el}}$, relative geometric preparation energies $\mathbf{\Delta E_{Geo\_prep}}$ and relative catalyst-substrate interaction energies $\mathbf{\Delta E_{int}}$ for five major and minor transition state conformers at the B3LYP-D4/def2-TZVP level of theory. All energies are in kcal mol^{-1}.

	ΔE_{el}	ΔE_{Geo_prep}	ΔE_{int}
**Major$_0$**	0.00	0.00	0.00
**Major$_1$**	0.37	0.56	-0.20
**Major$_2$**	2.58	1.95	0.63
**Major$_3$**	5.54	2.64	2.90
**Major$_4$**	7.03	5.59	1.44
**Minor$_0$**	3.70	6.13	-2.44
**Minor$_1$**	4.46	8.28	-3.82
**Minor$_2$**	6.17	1.78	4.39
**Minor$_3$**	6.65	7.85	-1.20
**Minor$_4$**	9.57	13.38	-3.81

S3 3D Structures of selected conformers

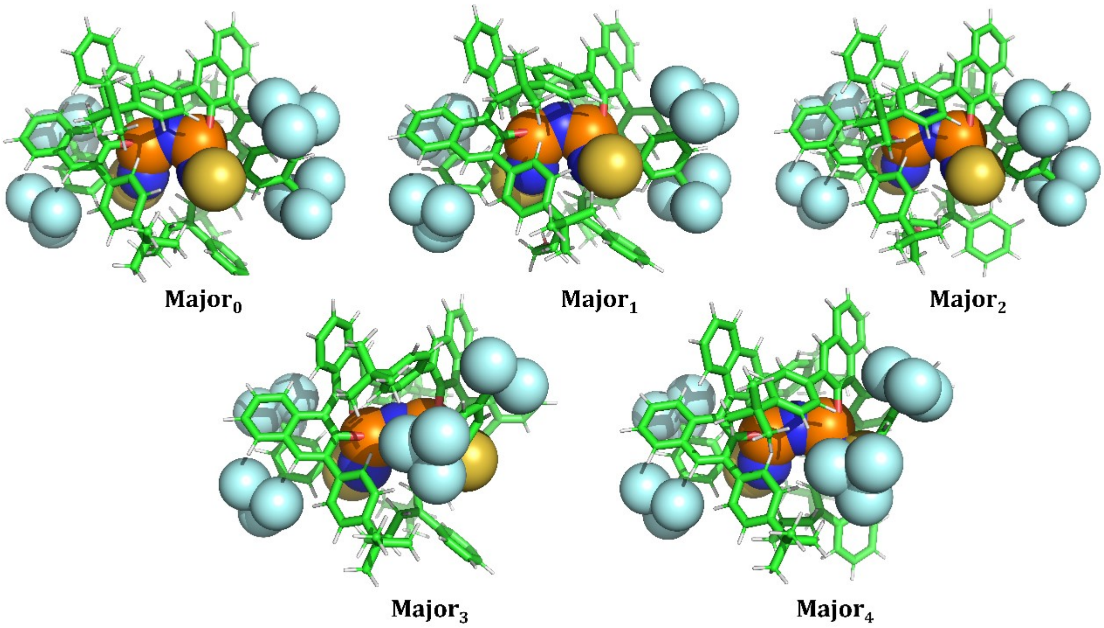

Figure S1. 3D structures of **$Major_0$ to $Major_4$** highlighting the core catalyst and the Ph-(CF_3)$_2$ groups.

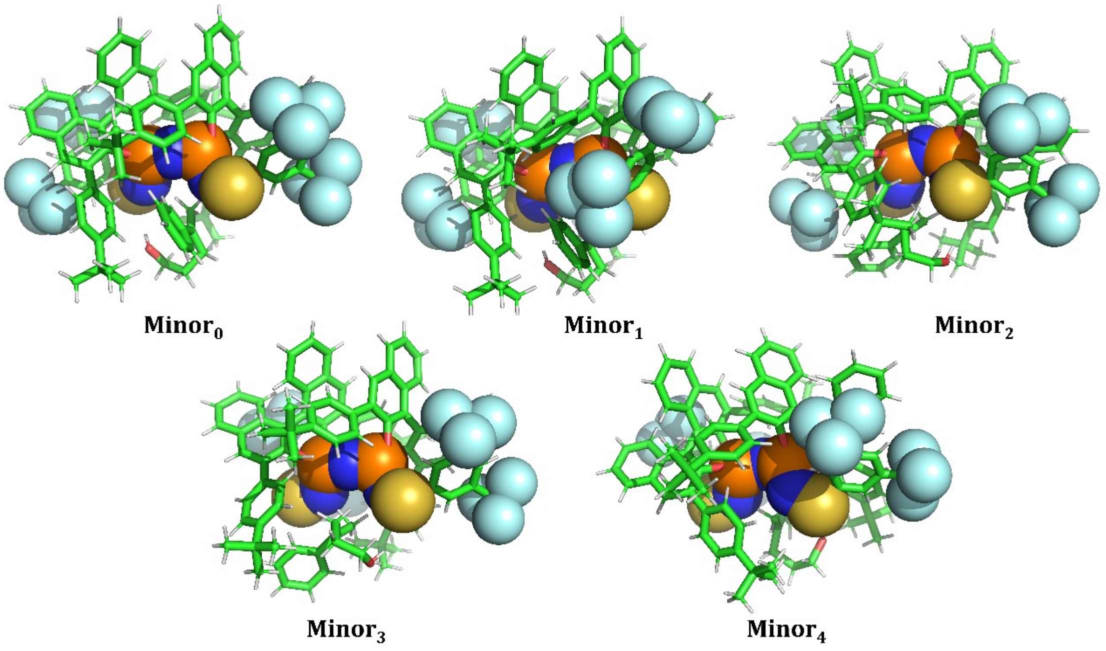

Figure S2. 3D structures of **$Minor_0$ to $Minor_4$** highlighting the core catalyst and the Ph-(CF_3)$_2$ groups.

S4 Hybrid implicit/explicit solvation model: Computational details and number of optimized conformers at each step

The hybrid explicit/implicit solvation was carried out in an iterative manner, adding one solvent (cyclohexane) molecule at each step to the most stable conformer of both the major and minor stereoisomers of the TS complex ($\mathbf{Major_0}$ and $\mathbf{Minor_0}$, respectively). The starting geometries of the conformers without solvent molecules were obtained from a previous work by our group[1]. Conformational sampling in the presence of a single solvent molecule was performed using the Conformer–Rotamer Ensemble Sampling Tool[2, 3] (CREST) from the Extended Tight-Binding Program Package[4-6] (xTB), at the xTB-GFN-FF[7] levels of theory. For the metadynamics simulations, a simulation time of 50 ps was chosen. In addition, the solute was kept frozen at its DFT-optimized TS structure. In order to avoid dissociation of the solute-solvent aggregate, an ellipsoid wall potential[3] was used. Afterwards, the whole conformer ensemble was optimized at the GFN2-xTB level, again with fixed solute. For the next iteration, another solvent molecule was added to the most stable optimized structure and the above conformational sampling was reapplied. This scheme was repeated up to the addition of four solvent molecules. The most stable conformers with significantly different structural features were subjected to constrained geometry optimizations PBE-D3[8]/def2-SVP level of theory. Afterwards, fully relaxed transition state geometries were obtained using Grimme's r2SCAN-3c composite method[9]. From this protocol we obtained the lowest-in energy transition states for the major and minor enantiomeric pathways with 0 to 4 added solvent molecules. The number of TS conformers optimized at the xTB and DFT level at each step are reported in Table S4.

Table S4. Numbers of xTB/DFT geometry optimizations performed for $\mathbf{Major_0}$ and $\mathbf{Minor_0}$ with one to four cyclohexane (CyH) molecules.

	xTB	DFT
1 CyH	1382	78
2 CyH	469	59
3 CyH	809	43
4 CyH	884	35

For the solvated system with four solvent molecules the xTB optimized structures together with their relative electronic energies are given in **Figure S3** and **Figure S4**.

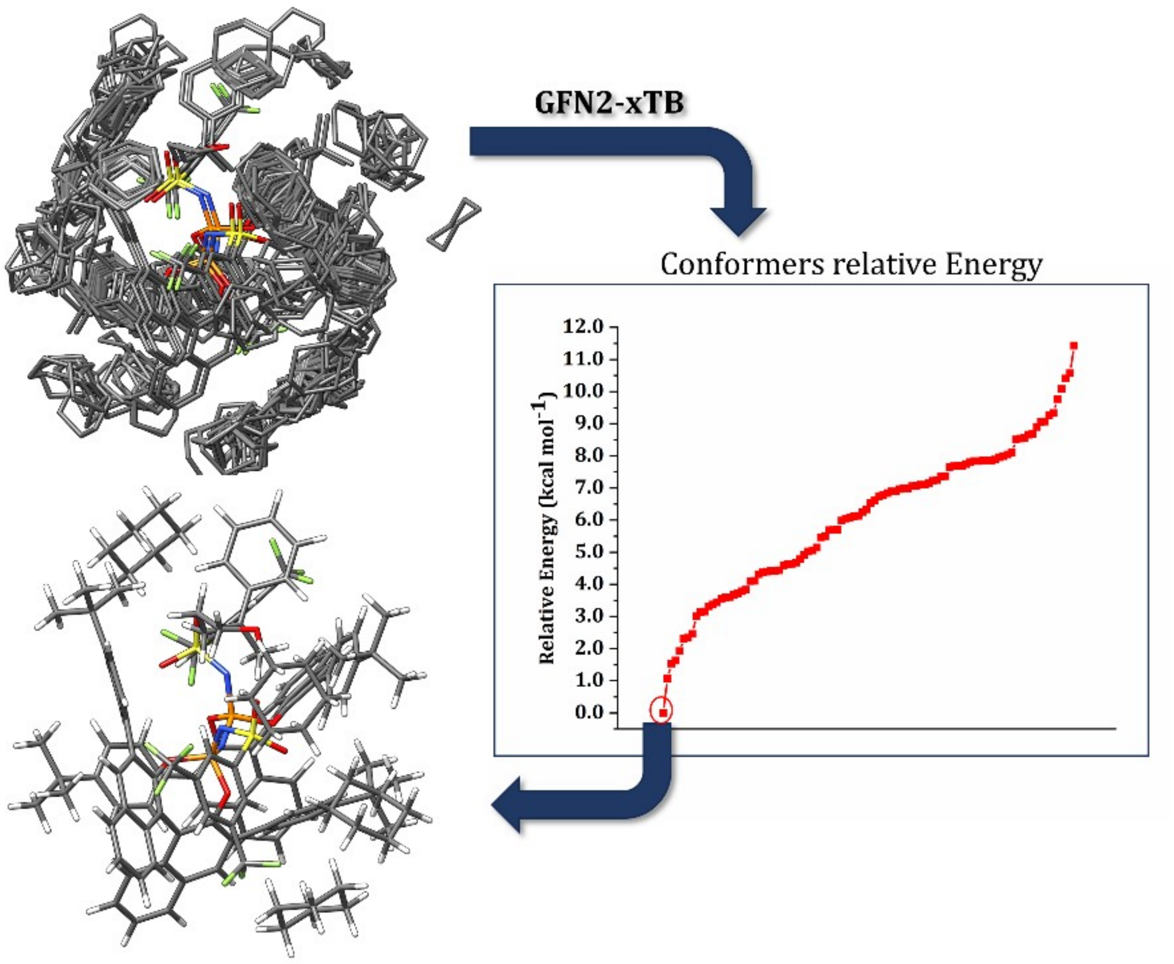

Figure S3. Low energy conformers for **Major$_0$** with 4 CyH molecules optimized at the GFN2-xTB level. Only the solvent network was optimized while the transition state was kept frozen in its DFT optimized structure. For clarity, the hydrogen atoms are omitted in the overlay of the individual conformers. The lowest-in energy conformer is shown at the bottom.

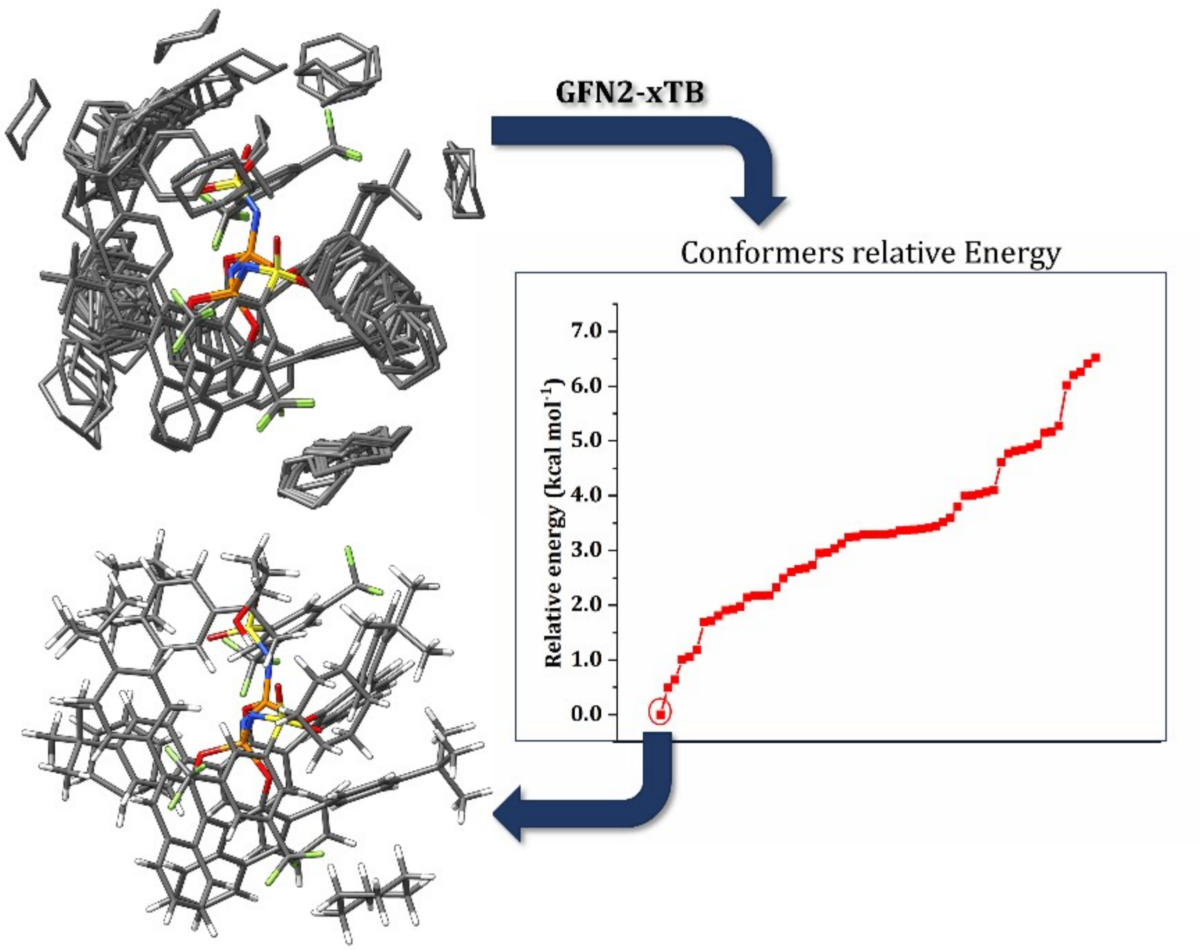

Figure S4. Low energy conformers for **Minor$_0$** with 4 CyH molecules optimized at the GFN2-xTB level. Only the solvent network was optimized while the transition state was kept frozen in its DFT optimized structure. For clarity, the hydrogen atoms are omitted in the overlay of the individual conformers. The lowest-in energy conformer is shown at the bottom.

S5 Effect of hybrid explicit/implicit solvation models on the relative stability of TS conformers

Table S5. Relative electronic energies (in kcal/mol) between **$Minor_0$** and **$Major_0$** with 0 – 4 solvent molecules at different levels of theory.

	B3LYP-D3	M06-2X	ωB97M-V
	ΔE_{adduct}	ΔE_{adduct}	ΔE_{adduct}
0 CyH	3.38	7.47	5.98
1 CyH	3.88	8.1	6.65
2 CyH	4.79	8.86	8.25
3 CyH	4.24	8.75	7.04
4 CyH	2.76	8.02	5.94

S6 Influence of explicit solvation on transition state structures

To test the influence of explicit solvation on the transition state geometry, the geometries of the fully relaxed transition states optimized *in vacuo* are compared with the fully relaxed transition states explicitly solvated by four cyclohexane molecules (**Figure S5)**.

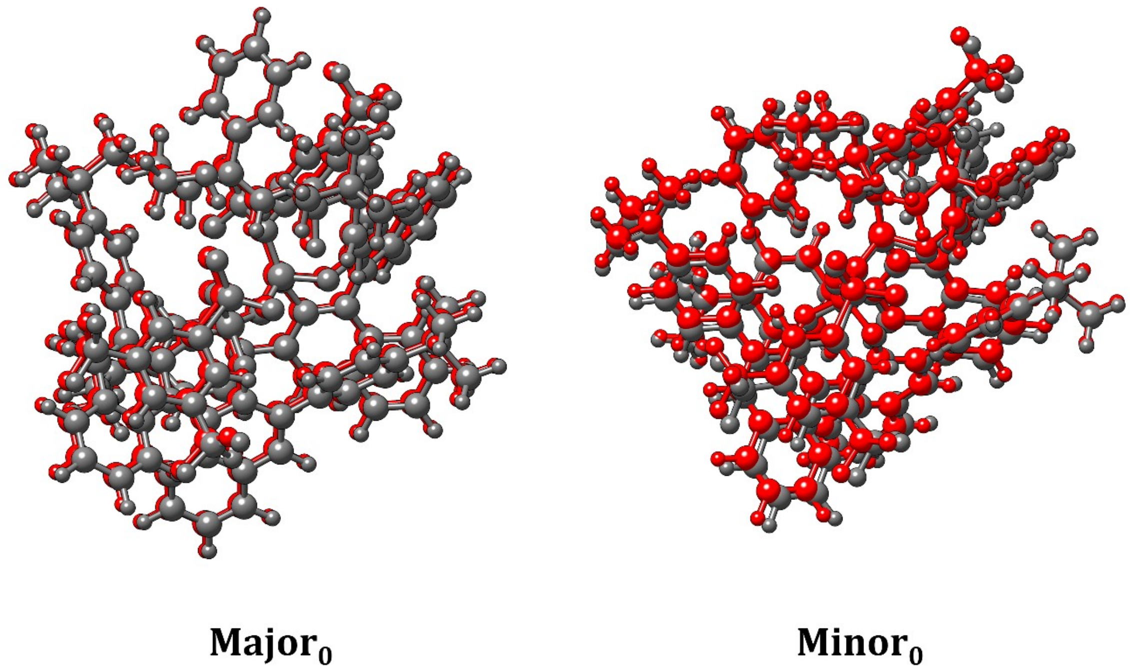

Figure S5. Optimally aligned transition state structures of the transition states optimized at the r2SCAN-3c level of theory in gas phase (dim grey) and with four explicit cyclohexane molecules (red) for **Major$_0$** and **Minor$_0$**. RSMD: 0.09 Å (**Major$_0$**), 0.32 Å (**Minor$_0$**).

As can be seen from the small RMSD values, implicit solvation has rather small impact on the transition state structure. As expected, the largest deviations in structure occur for the groups that are in direct interaction with the solvent molecules.

S7 References

(1) Harden, I.; Neese, F.; Bistoni, G. An induced-fit model for asymmetric organocatalytic reactions: a case study of the activation of olefins via chiral Brønsted acid catalysts. *Chemical Science* **2022**, *13* (30), 8848-8859.
(2) Grimme, S. Exploration of chemical compound, conformer, and reaction space with meta-dynamics simulations based on tight-binding quantum chemical calculations. *Journal of Chemical Theory and Computation* **2019**, *15* (5), 2847-2862.
(3) Pracht, P.; Bohle, F.; Grimme, S. Automated exploration of the low-energy chemical space with fast quantum chemical methods. *Physical Chemistry Chemical Physics* **2020**, *22* (14), 7169-7192.
(4) Bannwarth, C.; Ehlert, S.; Grimme, S. GFN2-xTB—An accurate and broadly parametrized self-consistent tight-binding quantum chemical method with multipole electrostatics and density-dependent dispersion contributions. *Journal of chemical theory and computation* **2019**, *15* (3), 1652-1671.
(5) Bannwarth, C.; Caldeweyher, E.; Ehlert, S.; Hansen, A.; Pracht, P.; Seibert, J.; Spicher, S.; Grimme, S. Extended tight-binding quantum chemistry methods. *Wiley Interdisciplinary Reviews: Computational Molecular Science* **2021**, *11* (2), e1493.
(6) Grimme, S.; Bannwarth, C.; Shushkov, P. A robust and accurate tight-binding quantum chemical method for structures, vibrational frequencies, and noncovalent interactions of large molecular systems parametrized for all spd-block elements (Z= 1–86). *Journal of Chemical Theory and Computation* **2017**, *13* (5), 1989-2009.
(7) Spicher, S.; Grimme, S. Robust atomistic modeling of materials, organometallic, and biochemical systems. *Angewandte Chemie International Edition* **2020**, *59* (36), 15665-15673.
(8) Becke, A. D. Density-functional thermochemistry. III. The role of exact exchange. *The Journal of Chemical Physics* **1993**, *98* (7), 5648-5652. DOI: 10.1063/1.464913 (acccessed 7/3/2023).
(9) Grimme, S.; Hansen, A.; Ehlert, S.; Mewes, J.-M. r2SCAN-3c: A "Swiss army knife" composite electronic-structure method. *The Journal of Chemical Physics* **2021**, *154* (6). DOI: 10.1063/5.0040021 (acccessed 7/3/2023).

S8 XYZ Coordinates

228

Major0

```
C   0.95233617156085    -4.15906190571433    -0.19026716977022
C   -0.24753804718309    -4.26749649187697     0.69994190794283
C   -0.94871694601085    -5.63413049101322     0.68786552371341
C   -1.73537055920114    -5.87034627138391    -0.59855393129617
O   -0.91758569956540    -5.69570473954126    -1.75830891151776
H   0.10599264318222    -4.05873400890095     1.72420346312513
H   -1.64241876675060    -5.68195483610246     1.54234663540330
H   -2.59809123593921    -5.18432173125433    -0.63968355659230
C   2.03262218463091    -5.15233466427240    -0.08522917323837
C   2.76703020805937    -5.50205306429230    -1.23250990662907
C   2.36531795646483    -5.76273203148524     1.13788446120265
C   3.81188858793088    -6.41872992676378    -1.15931069714763
H   2.48719968429815    -5.06766742062576    -2.19140301892670
C   3.42170867543421    -6.66627536767062     1.21322940603732
H   1.82742374741006    -5.48542307364903     2.04279251593045
C   4.14832226716641    -6.99863653928534     0.06623909481319
H   4.36177905282858    -6.68696601555740    -2.06307805262739
H   3.68310460644303    -7.11218563304252     2.17400552188540
H   4.97166738145748    -7.71204357015865     0.12769373227756
C   1.11618447328953    -3.05534745848289    -1.00774865073004
H   1.97192031010427    -3.00981671199697    -1.68357203425939
H   0.22275687188955    -2.49981116039524    -1.30101355292971
H   2.83132237995775     5.31350316931467    -5.17397273816990
C   2.63841525247914     4.51030865127845    -5.89896521936860
C   2.52100797095152     3.13802421247037    -5.20210419312245
H   3.46727903989806     4.49783112759272    -6.62314914684524
H   1.71205513900323     4.75595848167494    -6.43791342644336
C   1.34863705129541     3.19171550780011    -4.21655810862497
C   3.83294427207812     2.84425200449640    -4.44784146247932
C   2.32965102726600     2.05996324984914    -6.27809315136995
C   2.42142823800652    -0.71918778913537    -3.38836500846461
C   1.37128333212036     4.10451470559794    -3.14851193046724
C   0.21102640277230     2.38273597254100    -4.33783943151503
H   4.01777224900658     3.58188309818390    -3.65579107364104
H   3.81076998492020     1.84866260637951    -3.98506120844133
H   4.68337586417282     2.87394491592085    -5.14587445770082
H   1.43076154485119     2.24514919763230    -6.88386627423481
H   3.19484101473064     2.06203725565085    -6.95688295906270
H   2.25357124755459     1.05451382051254    -5.83955274910002
C   3.63599233179721    -0.95513908335665    -2.71441269194502
C   1.86507391546664    -1.68378986873081    -4.21961429057387
H   1.89610495569866     0.22546078615892    -3.26405209488161
C   0.30661545276972     4.21546524949932    -2.26211597919267
H   2.24237076963084     4.74331639130305    -2.99607111772145
C   -0.86202327953648     2.48816957108766    -3.45244768053711
H   0.14209064809812     1.64934319548331    -5.14071793229835
C   4.27863675109365     0.06379181929699    -1.85364968344656
```

C	4.26137139377547	-2.19330018521168	-2.92909038586293
C	2.48899900234451	-2.92309696968888	-4.44151273523256
H	0.90444265622070	-1.46241778942881	-4.68747081966583
C	-0.84167328804357	3.41653461155415	-2.40141596809218
H	0.37821870466847	4.91394461186443	-1.42722758095851
H	-1.72140076322563	1.83570215626111	-3.59154853379092
C	3.52949402581549	0.98886434743368	-1.07086518427382
C	5.65843373501779	0.19139831030512	-1.80974801259561
C	3.70529563125084	-3.14737876663851	-3.78218753621549
H	5.18727437012124	-2.43017892599396	-2.40332116559862
C	1.82314926421258	-3.96198289044769	-5.34601086464547
C	-1.97874292801035	3.60328107888541	-1.47266978407708
O	2.13908397006995	0.85719785362168	-1.08574035868642
C	4.09165959425263	2.04371051892432	-0.36217142059437
H	6.27410521148608	-0.47041670779349	-2.42050362276404
C	6.30362397402298	1.15362319400904	-1.00026748252114
H	4.23082725974063	-4.09341644870585	-3.91001600868027
C	1.61962574241689	-3.35470160669893	-6.74952416523317
C	0.45236111310900	-4.34189375287590	-4.74767084579351
C	2.66605598496504	-5.23673530631337	-5.48869156863794
C	-2.72250357620906	2.51312318317732	-0.94131986175886
C	-2.35658213561647	4.87007952119729	-1.05702284482358
P	1.34287617985751	0.46828302785012	0.26346057533700
C	5.51945607307752	2.09781681050593	-0.25755257194570
C	3.22211231921537	3.09455092274136	0.23298624788276
C	7.71952230601267	1.20411352521896	-0.90437255052881
H	2.58257606403061	-3.06914707913852	-7.19783191800160
H	1.13489533826913	-4.08888568311883	-7.41028439922581
H	0.98205698949193	-2.46087759674158	-6.71563627921382
H	-0.20038648628493	-3.46598582392338	-4.63414007157716
H	-0.05743904830570	-5.06468290544957	-5.40198106522790
H	0.56753547735662	-4.80802466285425	-3.75874315296356
H	2.80559224873440	-5.74633042566778	-4.52369007506637
H	2.15281261904067	-5.94120711183416	-6.15833348210230
H	3.65688888134322	-5.02677445634162	-5.91813519444101
O	-2.36821462193125	1.22451950233954	-1.34902373325617
C	-3.77888127936038	2.64314809478410	-0.05096784062466
H	-1.83197439736682	5.73847944837382	-1.46044046967965
C	-3.39914871400898	5.08113058628905	-0.12452936634764
O	2.02746235035917	1.38797465253757	1.39428369264678
N	-0.12707285037946	0.84463560271572	-0.05012817681026
N	1.72288696493921	-1.05088371235938	0.74477703934316
C	6.18959871001132	3.02153731188034	0.58449246102975
C	3.36045176229976	4.47553310673391	-0.13284829874359
C	2.19099954530153	2.75013099112557	1.09158835024376
H	8.30636470575474	0.48370193639081	-1.47713964884910
C	8.34077085473754	2.12075657318723	-0.08419953819856
P	-1.57128922491953	0.27348513089110	-0.30705019616286
C	-4.46230646574843	1.43225167914158	0.47844305485587
C	-4.12343041054539	3.95940081237073	0.40276386438844

C	-3.72179765929130	6.38892794536355	0.32694899664183
S	2.28005995695483	-1.48021436838555	2.23485699175324
H	1.46403335610246	-2.09273734827305	-0.04711423567888
C	7.56627954874955	3.02891811275515	0.67310247337669
H	5.60177689610278	3.71818489388817	1.18072688135273
C	4.30274634042578	4.93407165568378	-1.09019882693447
C	2.46455320674828	5.43369441589425	0.45067050377059
C	1.28474265351546	3.67150578318797	1.68341873435303
H	9.42893626644130	2.14069721655693	-0.00937090537249
O	-2.32524875279837	0.56141651312202	1.09558433303368
N	-1.77060817677712	-1.23614813898999	-0.77070673797357
C	-3.71705948615344	0.43458632230623	1.09423638710176
C	-5.88059916409224	1.25649711710968	0.38590828464645
C	-5.11013001003307	4.20390116684215	1.39391569409284
H	-3.16934683554424	7.23298694280007	-0.09158627740425
C	-4.69546336577627	6.59186096687255	1.28018040893956
O	2.25312253358583	-2.93509584996030	2.24641003017099
O	1.62842848665395	-0.73932036214465	3.30202982193566
H	8.05974761620234	3.73381555547262	1.34327832882212
H	4.97104658453156	4.21768728341439	-1.56447779710217
C	4.37569404549590	6.26779681408926	-1.43638908714380
C	2.57774235172739	6.80145601077119	0.08396510307543
C	1.46347752115216	5.00511817432560	1.35343740952561
C	0.19235963048819	3.25037977112525	2.58743391864571
S	-2.23891941041351	-1.71289846349140	-2.22905016753540
C	-4.27983930800286	-0.69357447790243	1.75504973685284
C	-6.47900846265822	0.13074134611762	1.04376721886708
C	-6.71427970041598	2.12378408081119	-0.36532794429485
C	-5.38773812648663	5.48560042200271	1.82306504120382
H	-5.64986021368815	3.36223336185697	1.82435182746664
H	-4.92782521331999	7.60109890287998	1.62286098716296
H	5.10340294791270	6.59195290120507	-2.18188665030708
C	3.51236550246373	7.21446002432874	-0.83996199987093
H	1.89484773347948	7.51947237115284	0.54300335708405
H	0.80579320806452	5.75276534828468	1.80106639819948
C	0.37470541041859	2.29310145256931	3.59462554057609
C	-1.07692151215207	3.84174196527516	2.47100086894282
O	-1.63734835192157	-0.95395535396529	-3.32363522357607
O	-2.10606096692279	-3.17242391046300	-2.25978375986724
C	-5.66278525790325	-0.79242841383729	1.73611529450410
C	-3.46207569183483	-1.69885129570568	2.47162251488803
C	-7.88591275344876	-0.04571584232466	0.96872971178858
C	-8.07549359260768	1.91036648148087	-0.43789802739147
H	-6.26472684899355	2.95549819578111	-0.90629944860714
H	-6.14544464923010	5.64437310193672	2.59188775146931
H	3.58308115045881	8.26686218219235	-1.11807725769685
C	-0.66488115060106	1.95783904608785	4.46185489564676
H	1.33543967109604	1.79610767761832	3.71162909446171
C	-2.10618547457685	3.50623065855354	3.34276033554649
H	-1.26817119603383	4.55282788881636	1.66572107421315

H	-6.14491452082573	-1.61352742911619	2.26816354830546
C	-2.29840850227452	-1.36070937703840	3.18846076224433
C	-3.88179168770901	-3.03728453652009	2.52047532276554
H	-8.33051805178554	-0.90352154710127	1.47670089002882
C	-8.67075379332529	0.82481619972815	0.24402955079031
H	-8.69448659929987	2.57810428766764	-1.03845491593934
F	5.50294116487860	2.60681300520052	3.80405038432170
C	-1.92600001930228	2.56080020270586	4.36648779283398
H	-0.47050725960150	1.20793884762952	5.22793150160380
H	-3.07484045137313	3.98900126046855	3.20773308049863
C	-1.62486159035388	-2.31144690358167	3.94773646914565
H	-1.92204384422687	-0.33979918050104	3.17393690009760
C	-3.20850790618639	-3.98080087536170	3.29691540391059
H	-4.75300884034587	-3.34992212706765	1.94202163358822
H	-9.74872372726339	0.66907785956770	0.18210656778668
C	6.25298626806435	1.48067004345431	3.96223037131555
C	-3.06829552910866	2.25903976448951	5.34249706639002
C	-2.07566049602777	-3.63842631462738	4.04759413233784
H	-0.72764331873673	-1.99572677739088	4.48269514243242
H	-3.59032329531320	-5.00140468321707	3.31662736708111
F	6.13214892357340	1.11476714723594	5.27088817026800
C	5.79668472608646	0.37074253350739	3.04806583497049
F	7.55071757696061	1.82184000450476	3.76257224486848
C	-2.71932442298432	1.12679322461933	6.31909075654145
C	-4.33745417920055	1.85451525519199	4.56757353130664
C	-3.36131649226852	3.53828757411123	6.15504140555649
C	-1.35601196618926	-4.62669956006515	4.97185397535700
C	6.71400240664016	-0.36432686635372	2.29933791848726
C	4.43569395477682	0.05491958997772	3.01589502581415
H	-3.57255235223247	0.94588768110095	6.98886857214882
H	-2.50422607952336	0.18548702786537	5.79234792806455
H	-1.85337598118365	1.38069745577078	6.94766587875251
H	-4.17315808378632	0.93693154704522	3.98751973254611
H	-5.16413328279439	1.66971424050895	5.26998035984682
H	-4.65558872790091	2.64270226198784	3.87273420759453
H	-3.65384084690943	4.37001183898944	5.49863662860971
H	-4.18280719726840	3.35802482781862	6.86520881179620
H	-2.47465894170007	3.85265143400704	6.72449817956601
C	0.08897753350958	-4.84663747321513	4.48369210327968
C	-2.06021359154608	-5.98965118268101	5.02662204115245
C	-1.32449608745403	-4.03994084274536	6.39899744288066
C	6.26254682817511	-1.42920395756248	1.51383701888873
H	7.77136730663604	-0.10862277440841	2.32148288065473
C	4.00845402283765	-1.00574254401820	2.22503408939020
H	3.71158005414620	0.61488263494446	3.60617978218994
H	0.63782380089239	-3.90279269719067	4.36460231381788
H	0.63941327435624	-5.47653222904434	5.19916052868285
H	0.08808240866697	-5.36328388952194	3.51319534755062
H	-1.51991393357736	-6.65472001325843	5.71557483399698
H	-3.09486592335291	-5.90039456109294	5.38872892635938

H	-2.07895028213391	-6.47942479607353	4.04182046660477
H	-0.83735739639092	-4.74712891040455	7.08714802506651
H	-0.76566039415207	-3.09518475560115	6.43541991413356
H	-2.34289892425974	-3.84653367905476	6.76580908974652
C	4.90824026779812	-1.75831450060743	1.46959078180900
C	7.25097358328149	-2.29367207930659	0.77170762175331
H	4.55385845087126	-2.60016655653353	0.87369675088543
F	8.43247575109014	-1.66127102634288	0.55328615866371
F	6.77371141232601	-2.69100153147645	-0.44192676667335
F	7.53140966471249	-3.43035148870508	1.47000665894857
C	-4.87434602555718	-2.29308311555851	-1.64788062486447
C	-4.01063958194956	-1.41111094359788	-2.29586320933251
H	-4.47041711911316	-3.14212036447214	-1.09679180050204
C	-6.25125646252246	-2.08456649328304	-1.73716834849512
C	-4.50246501528126	-0.33046968072756	-3.02134122155997
C	-6.76383115674427	-0.99924104444279	-2.45117322170237
C	-7.17880927690048	-3.09440104248325	-1.11159061409565
C	-5.88287314895966	-0.12557049022782	-3.08782318195711
H	-3.80879693302714	0.33382661521327	-3.53450000061069
H	-7.83755096283026	-0.83326039423037	-2.50377062965896
F	-6.78514334402620	-3.43793373000327	0.15101223556858
F	-7.20692519530902	-4.25160491276361	-1.83239607803648
F	-8.45801075868718	-2.64918465939598	-1.02522031636818
C	-6.39695475672920	1.03064783637668	-3.90714578246734
F	-7.72435420603451	1.25176061552440	-3.72624711262219
F	-5.74617381503272	2.19047754845309	-3.60650920364775
F	-6.20615135414728	0.81490297765231	-5.24083359141759
H	-1.15418560410016	-4.81828522974948	-2.12354976975354
H	-2.12529141753192	-6.90206084630949	-0.60972846710184
H	-0.21017208117846	-6.43925747823175	0.82039834358377
H	-0.95625846179399	-3.46421641536478	0.45091780169297

228
Major1

C	1.14225135555243	-4.10623797884394	0.38891768615733
C	-0.12267090111808	-4.26182411554462	1.17806019823149
C	-0.69837550141657	-5.68857369030530	1.21552717337449
C	-1.42128627311534	-6.06587903847399	-0.07419290770109
O	-0.58558925457388	-5.91383264120383	-1.22292472967566
H	0.10816037705846	-3.94024008513117	2.20786412981202
H	-1.41364074127352	-5.75806704531748	2.05029151744243
H	-2.32904533517654	-5.44888155846452	-0.18392341198646
C	2.32064526292484	-4.91710776337191	0.73547962800315
C	3.28131700938217	-5.21845661108600	-0.24835985639351
C	2.53707612038339	-5.38178403927671	2.04655987596055
C	4.43248066248908	-5.93221905137966	0.07304213759073
H	3.10586180330778	-4.90572611889331	-1.27702380004337
C	3.69338872151347	-6.08568048744309	2.36910552297130
H	1.81873190557203	-5.14165758400849	2.82868008333153

C	4.64758620152721	-6.36028326828516	1.38523747095717
H	5.16443904571179	-6.15913685429458	-0.70374429798407
H	3.85619769609648	-6.41561714032941	3.39609945603393
H	5.55431661598994	-6.91082751636420	1.64052779918650
C	1.24865295683720	-3.11315394235042	-0.56678544847773
H	2.14949951001081	-3.04153464187481	-1.17916815380351
H	0.32701454165173	-2.71982459984874	-0.99864352667110
H	1.48691668086260	3.87612365712271	-7.04091020343598
C	2.40545266101846	3.78743710684691	-6.44287513378594
C	2.34411893069250	2.54813312656914	-5.52531641592250
H	2.52004951925889	4.71226423029115	-5.86008087740147
H	3.26018101322037	3.71040309618417	-7.13238579438977
C	1.14733838403762	2.70196513878432	-4.58055652616395
C	3.64665249193287	2.46224790321187	-4.70584191811005
C	2.24218930618302	1.29350921697632	-6.40360878962546
C	2.41071257470515	-1.08336844715401	-3.26335742066604
C	0.05232416467921	1.82871280759897	-4.59001189280881
C	1.10526364365026	3.76987239453502	-3.66731370301713
H	4.51344033692515	2.38203960608697	-5.37932151915820
H	3.78538947666035	3.35211764159067	-4.07824783245429
H	3.64347516130772	1.58233953007582	-4.04869125440256
H	1.35298016249500	1.31670420842086	-7.05035611152726
H	3.12534706103102	1.23265637952124	-7.05605574811324
H	2.21148867071840	0.37449241297857	-5.80087621956486
C	3.63506978576231	-1.21491222190058	-2.57737966124581
C	1.88583203482297	-2.13660747142290	-4.00228625399045
H	1.85271745023761	-0.15092789221095	-3.22317415605385
C	-1.03906940147023	2.01127460491155	-3.74091297532278
H	0.03363151074839	0.97814188479239	-5.27091491574994
C	0.02056646570295	3.95838441168854	-2.81834055551425
H	1.94376193563408	4.46508190182369	-3.60238241002594
C	4.24810579762197	-0.09985621056263	-1.81896815745320
C	4.30234389433249	-2.44559301213006	-2.68740288915747
C	2.55489179006898	-3.36592183986871	-4.12348214597815
H	0.91671565560464	-1.99026832443933	-4.48174702987347
C	-1.08200256746907	3.08636548251506	-2.84159233540449
H	-1.85925177217483	1.29819285563042	-3.78401851675437
H	0.04113242041184	4.77860582829652	-2.09919716178834
C	3.47630712373115	0.90897289070336	-1.17129073436269
C	5.62656896632597	0.04313423479431	-1.75674989322186
C	3.77933796970519	-3.48864841568034	-3.45232385047396
H	5.23727093841398	-2.60316069848526	-2.14901400701835
C	1.93535333595111	-4.49685453157822	-4.94620835987695
C	-2.23272332979408	3.33399319770100	-1.94392818332637
O	2.08706446592600	0.75905077244076	-1.17981445356609
C	4.01896317574549	2.05991425027767	-0.61130300648597
H	6.26059165312350	-0.68307769460215	-2.26717541368226
C	6.24871326777993	1.10361978896723	-1.06056148802153
H	4.34080643098779	-4.42110987067509	-3.51031549127895
C	0.59189340684529	-4.90282709061435	-4.30630145108138

C	2.84020421009908	-5.73498283838160	-5.00635437571077
C	1.69221618822541	-3.99863194578002	-6.38591740426939
C	-2.90679679359973	2.28367433233282	-1.26028164039962
C	-2.68934043711575	4.61959068626078	-1.70204591861749
P	1.26272845272096	0.59197100291314	0.19941954937314
C	5.44275926332396	2.13769190333636	-0.47948443619407
C	3.13390413511986	3.18445989641332	-0.20315808528980
C	7.66113575265201	1.17260562728793	-0.92933534312395
H	-0.09408519028658	-4.04926860436481	-4.22072304116944
H	0.10111844497866	-5.67515662055137	-4.91729615266580
H	0.74852351434843	-5.31881825887701	-3.30137166292634
H	3.01204007682069	-6.16261333182737	-4.00747558140449
H	2.35839608993549	-6.51162056829324	-5.61719033259881
H	3.81565960913166	-5.50788278761794	-5.46191892115209
H	1.00465369973540	-3.14230870801991	-6.40785271175521
H	2.63532167909241	-3.68992532168326	-6.86035443151641
H	1.24771012010549	-4.80252729679637	-6.99139437927639
O	-2.47421909167875	0.97623112337533	-1.49307655596731
C	-3.96519597319693	2.46709006841740	-0.38209024266438
H	-2.22107904925885	5.45620930889682	-2.22445136888475
C	-3.74096805821152	4.89048268956345	-0.79509967754272
O	1.93496791972940	1.66851123378681	1.18837681804366
N	-0.20060840077631	0.92442540288454	-0.19757321793731
N	1.60822293758786	-0.83968496347466	0.91793755821698
C	6.08671373868127	3.17727715990345	0.23909859923288
C	3.26730118668847	4.49120269207650	-0.78168113348962
C	2.09183004104785	2.97057345957034	0.68226490136955
H	8.26520722960483	0.38171845250436	-1.37793693059668
C	8.25676387653912	2.19941692018721	-0.22927804934974
P	-1.60869831816929	0.22922109209463	-0.34577962167887
C	-4.56520639742933	1.29632447867445	0.31517511093533
C	-4.39122876635210	3.80925440725062	-0.10955454387510
C	-4.14459123930065	6.22525455820833	-0.52362699871575
S	2.13309733035949	-1.04341592815220	2.46590807818259
H	1.43007299680805	-1.99219018902292	0.27021055039284
C	7.46000070127817	3.20419987374025	0.36558537920340
H	5.47995901462529	3.94920629040732	0.71088989850653
C	4.23087951430166	4.80400441342017	-1.77530750975189
C	2.34429454582326	5.51599870892847	-0.38231301310205
C	1.16842973191903	3.96332719815360	1.10802219613034
H	9.34211959570118	2.23453517539221	-0.12491759837987
O	-2.37207242636882	0.65517254199280	1.01581456142327
N	-1.70850963071372	-1.33980883099098	-0.60160559638349
C	-3.75295140221891	0.44015272364052	1.04802828629459
C	-5.96803956894286	1.01157393942509	0.26023071418434
C	-5.38946217990250	4.12635832161215	0.84898881784307
H	-3.64738633912065	7.03644643036007	-1.05985421651331
C	-5.12617277182523	6.49684832491357	0.40404049894870
O	2.04091636085514	-2.47425519674625	2.71394560717554
O	1.51137456701723	-0.10841076549545	3.38708944897337

H	7.93351981330710	4.00019853769772	0.94140403660031
H	4.92227906909151	4.03130077531341	-2.10561279099463
C	4.29402307030449	6.06385641097934	-2.33477113650145
C	2.44714468014899	6.80617540606905	-0.96749901605102
C	1.33184346078471	5.22598324969331	0.56284997035922
C	0.08015414020220	3.67297471324450	2.06662294924609
S	-2.13723396632028	-2.02057568334680	-1.98936533217741
C	-4.23561795317297	-0.62703010168259	1.85664114636699
C	-6.48476126724447	-0.06501938741341	1.05585482149489
C	-6.86094001175238	1.71778277592679	-0.58577250744126
C	-5.74612150943091	5.43523244306674	1.10141604277441
H	-5.87279429007655	3.31957039520518	1.39718044216660
H	-5.42057822647030	7.52731421664279	0.60762026098990
H	5.03762503805619	6.27528583578134	-3.10462807411988
C	3.39984711025403	7.07846640690062	-1.92487407590354
H	1.74304374668395	7.57788152094414	-0.64915169983870
H	0.65628054808787	6.02640090253664	0.87130128348703
C	0.29305631297946	2.91038246903963	3.22254324710737
C	-1.20866075669502	4.18923728240146	1.85105639882089
O	-1.59458412513660	-1.35693020378134	-3.17314718634945
O	-1.89373228153223	-3.45952595362232	-1.84694458583322
C	-5.60625535361472	-0.83230450056525	1.85511386758189
C	-3.34336013187957	-1.45917815039239	2.69601217038730
C	-7.87472450906484	-0.35153576532283	1.01036684393377
C	-8.20285863137856	1.39920192891538	-0.62342606264875
H	-6.47015105027213	2.50792692575747	-1.22541701277223
H	-6.51092174572552	5.65114708990969	1.84901600292240
H	3.46173707948217	8.07165269036206	-2.37173005576361
C	-0.73756545661970	2.68832909195178	4.13489853289126
H	1.27249103390614	2.48007341049256	3.42133505616709
C	-2.23069591372945	3.96508082104717	2.76670193641629
H	-1.41972749840302	4.74667971213708	0.93700869013806
H	-6.02840081169185	-1.61576383345992	2.48591567807255
C	-3.63344024848535	-2.81874130260125	2.90556847685333
C	-2.22964030887210	-0.92710444206378	3.36681087046052
H	-8.25809484488982	-1.16986400829738	1.62275304807781
C	-8.71885541231043	0.36421044524699	0.18916688865515
H	-8.86747385899257	1.94258375271361	-1.29628457143046
F	6.53620612749081	-2.79631434146449	-0.01909186990096
C	-2.02035556338075	3.21504318410983	3.93634877391603
H	-0.52134543216076	2.08832844142065	5.01807535217603
H	-3.21747022213528	4.37868168568005	2.55401213022547
C	-2.87007660256401	-3.59322102674107	3.77549026722563
H	-4.46735041996035	-3.27849860889183	2.37176690241118
C	-1.46570013081969	-1.71167143664427	4.22840139820833
H	-1.95266885948899	0.11655911680535	3.22977781699505
H	-9.78240095544371	0.12390950790086	0.15332651514869
C	7.05919214085778	-2.23568644561279	1.10754726624237
C	-3.16118596628325	3.02466318416956	4.94231322704342
C	-1.77273274995064	-3.05774865479968	4.47080512266229

H	-3.14717777588588	-4.63904902090376	3.91643818855561
H	-0.60864910587944	-1.24619650467859	4.71250513927728
F	8.24397138650088	-1.66704178976494	0.76246852379802
C	6.10990693885901	-1.24846770967685	1.73910965562231
F	7.34350949244196	-3.26276975860087	1.95765297272867
C	-2.74940234280296	2.14791220301464	6.13337847559342
C	-4.36856043798538	2.36492821663452	4.24850540042043
C	-3.58160317962384	4.41070578738793	5.47471161694315
C	-1.00944602420715	-3.88953416932339	5.50823579194735
C	6.60613240679349	-0.10078209593618	2.36543416220855
C	4.74567648228600	-1.53498489369421	1.75205895235622
H	-2.45160252247275	1.13843827675255	5.81385185863393
H	-1.91855316494662	2.59369090664863	6.69937846585409
H	-3.60168251643544	2.04179466588628	6.81988878137611
H	-5.20447473459169	2.26584177690594	4.95752573526137
H	-4.71728720478435	2.96123060886581	3.39520450718827
H	-4.11486860394729	1.36191710746648	3.88047480054341
H	-2.73886272075174	4.91085171236966	5.97357623156438
H	-3.92926610631743	5.06323851970166	4.66153906507780
H	-4.40122378436620	4.30604156346918	6.20206161820721
C	-0.76439230356901	-5.32492204770806	5.00870067005627
C	-1.87321341745582	-3.95044851816994	6.78716352768491
C	0.35345841172827	-3.26931291783387	5.85549785355636
C	5.72312963262244	0.76237760750443	3.01178732803640
H	7.67153674407340	0.11951825929182	2.34378500345696
C	3.88018082767130	-0.65083657062357	2.39738223213943
H	4.35995553217228	-2.43932594768028	1.27974333932497
H	-0.16878417763922	-5.33065578766373	4.08540436379830
H	-0.21348002466370	-5.89425156807394	5.77174047469309
H	-1.70020289222683	-5.86510436877703	4.81033553123344
H	-2.05757704465368	-2.94059620072484	7.18075612278296
H	-2.84727116202234	-4.41806574512399	6.58367034248933
H	-1.36341333854393	-4.53828348498443	7.56610877718245
H	0.24861040835881	-2.29212221086671	6.34742978608686
H	0.88737022881803	-3.93011854268187	6.55405758048880
H	0.97778486280508	-3.13505183091157	4.96094140117308
C	4.35149143954160	0.49397589746498	3.02977409473320
C	6.22739980640976	1.96907973064642	3.76396061570261
H	3.65150201547104	1.15651949673208	3.53732408725553
F	6.12048850028945	1.78171695109925	5.11126059083947
F	5.50642515582200	3.08628918349093	3.46970645651793
F	7.53036919522829	2.24116941199741	3.50245829697542
C	-4.49956746570670	-0.92375843512736	-2.92797150016275
C	-3.92680158435980	-1.86264030699751	-2.07575732484979
H	-3.85767583064082	-0.28375578047793	-3.53119547703928
C	-5.89164568788517	-0.83045605225973	-3.00196295644659
C	-4.72020241215502	-2.71437630503202	-1.30840516846770
C	-6.70289687134283	-1.67682943671014	-2.24677150654567
C	-6.49335761291813	0.17086045878286	-3.95484326356962
C	-6.10902641441399	-2.62122510351526	-1.40634124282101

H	-4.25168000120248	-3.45248756040208	-0.65769419114155
H	-7.78614382883988	-1.59858738609845	-2.30699906176885
F	-6.27383684560120	-0.18782391056291	-5.25278782673979
F	-5.94467027840137	1.40934122393327	-3.79861692449951
F	-7.83602737048500	0.29986849864050	-3.79943801622758
C	-6.95552925499989	-3.60566948877009	-0.64097393547860
F	-8.26628294538204	-3.25570449675843	-0.60598713707111
F	-6.88885450497681	-4.85006506524957	-1.19468016414810
F	-6.53891675521496	-3.73951952743964	0.65364054845319
H	-0.84878649119722	-5.06637597079463	-1.63544761554507
H	-1.73679970961150	-7.12149011634653	-0.02299159571094
H	0.10309823028497	-6.41600464459986	1.41530763479335
H	-0.87536617913648	-3.55320929497250	0.80329718233546

228
Major4

C	1.03008268945352	4.31022921575620	-1.41769690325358
C	0.15648604484812	4.07118534810497	-2.61594029635138
C	-1.35190580978852	3.99376756338027	-2.35084613611549
C	-1.92201098207294	5.33843726393475	-1.91329582366510
O	-1.26604830316351	5.86942325679133	-0.76444697339227
H	0.37074590511257	4.82125366243936	-3.39046519474163
H	-1.57085726387891	3.22639073182938	-1.59348883972231
H	-3.00981223036538	5.23928052244957	-1.73633863450425
C	2.04812093149104	5.36293303915330	-1.49052527319807
C	1.76187000974933	6.59331439433536	-2.11104118752262
C	3.31412711246664	5.17988782997056	-0.90679792139345
C	2.70486699951695	7.61743267160103	-2.12011824573155
H	0.77086264404488	6.76709356709817	-2.52901844023106
C	4.26687129619657	6.19250894781808	-0.94888226361543
H	3.55702296784659	4.22505126200233	-0.44594228211707
C	3.96414526385051	7.41790173077740	-1.54823659912984
H	2.45685611521773	8.57650355409711	-2.57697822265178
H	5.25336788084885	6.02296233458463	-0.51407222033430
H	4.70827985710277	8.21568803170616	-1.57149590155671
C	0.95725584374653	3.46865074742643	-0.31969036249059
H	1.48647505420880	3.75147798006690	0.59260588302300
H	0.02076286290174	2.92041382225662	-0.17096586318318
H	1.72598174786092	1.13405501072999	7.32954263989197
C	2.66529098907313	1.08699339583246	6.75974854740187
C	2.95452112138612	-0.33167711099188	6.24943755488006
H	3.47560146094767	1.40622474093783	7.43121997480425
H	2.60905294175599	1.81186042798458	5.93470257947959
C	1.86922957666221	-0.84196235803936	5.29590086311322
C	3.01636126038658	-1.28821410397202	7.45986887953894
C	4.32581801318937	-0.32108966160470	5.54609149620750
C	2.38485102104902	2.35405180031810	3.06278224120586
C	0.67175498550277	-0.15295971522476	5.06196054600513

C	2.03057037220434	-2.07645762239611	4.64599053147016
H	3.26172531739758	-2.31311465845858	7.14701727543706
H	3.78752707619252	-0.95321156859215	8.17042296925014
H	2.05084281457318	-1.31718533267060	7.98526368721511
H	4.59154859859607	-1.31120674307461	5.15136132601104
H	4.33805834602050	0.38953007375510	4.70965200747625
H	5.10955281299019	-0.02229462769229	6.25848468346320
C	3.65188347785130	2.38277770517195	2.45614663315327
C	1.68324311267487	3.52964497855368	3.32772485646054
H	1.92452497807195	1.40480756871136	3.32693164097180
C	-0.33054340054858	-0.67768815923663	4.24558054855798
H	0.49695731158505	0.81760013466242	5.52549019176028
C	1.03679988481805	-2.60770595916660	3.83340921316533
H	2.95272304223674	-2.64479996275548	4.77141538806663
C	4.41032205368817	1.15660807823990	2.12437415866535
C	4.19729130808647	3.64630001156200	2.16380841591684
C	2.21090816413676	4.78829507171881	3.01001215934447
H	0.68603321246380	3.44172159440385	3.75635029022551
C	-0.17688733749068	-1.92899802217453	3.63049695223805
H	-1.24148181155474	-0.10102589783657	4.09939271763986
H	1.21066794540910	-3.56161089392990	3.33352204151542
C	3.77033234571796	-0.03905408483001	1.69272261824031
C	5.79418089322303	1.12363906927341	2.18993838448191
C	3.49605676324291	4.81479568073157	2.44092240608712
H	5.16751487060268	3.70871363795575	1.66722451757193
C	1.43994915327897	6.09295647321380	3.22710852584916
C	-1.25428528894414	-2.58085858215335	2.85425513138890
O	2.37461569837408	-0.01645382635748	1.56553868442879
C	4.43730627563852	-1.22148658444915	1.40408534200352
H	6.33086386689552	2.00380259750645	2.54846853025849
C	6.54602228217630	0.00078930203130	1.77491833715459
H	3.94531616329037	5.77076496741772	2.16757997443491
C	0.04954106820112	5.85017255281709	3.83094647920231
C	1.26177352944582	6.80930321620683	1.87147537612952
C	2.24306342827772	6.99878883754365	4.18302278943057
C	-2.11613010618200	-1.87370743991641	1.97025420755238
C	-1.47451412199608	-3.94456677894439	2.97268320443661
P	1.72570116297723	-0.10158425971110	0.09006586196880
C	5.86829285216553	-1.19250759558014	1.35868932439798
C	3.67252205416584	-2.47338670041354	1.16715856946960
C	7.96521551754306	0.03295786623879	1.74189198634611
H	0.11151028323207	5.37196748258388	4.81981482999522
H	-0.46463525544743	6.81375636133088	3.95863693545649
H	-0.57714578156050	5.22090731682160	3.18317435002755
H	2.22564262410707	7.02554474059948	1.38968295781243
H	0.66838596502022	6.20214699827640	1.17466620369357
H	0.73380721093095	7.76322911915318	2.01861749686544
H	3.23761178732629	7.23395695340250	3.77863818781389
H	1.70950953051025	7.94809036868205	4.34137451089079
H	2.38022418453728	6.51342445440769	5.16043955902003

O	-1.93201960378159	-0.49472718260453	1.84209261393735
C	-3.14566704246450	-2.45991972128747	1.24878714911630
H	-0.85711289882426	-4.52079604990748	3.66435098247906
C	-2.47251941540925	-4.61971066467407	2.23349183319510
O	2.44792187936333	-1.37999077182958	-0.57279435339556
N	0.21212863268882	-0.30267047250742	0.31847151203561
N	2.12567222747139	1.10582920847343	-0.94356817105789
C	6.63913966661032	-2.27735017893436	0.86642730643346
C	3.89355826365525	-3.63733343350992	1.97764330665077
C	2.67016737503591	-2.52388854275799	0.21129276108668
H	8.47310861795017	0.94097659106135	2.07356849509051
C	8.68761650997378	-1.04367691327989	1.27490465149052
P	-1.29356088209146	0.09865186202046	0.47544957885011
C	-3.99197740051220	-1.63943336861243	0.34299478644770
C	-3.32196898978885	-3.88016428912389	1.34437314030208
C	-2.62948293471690	-6.02831124665264	2.33303836934697
S	3.53048927096880	1.30397592476606	-1.80889493368674
H	1.58682102837691	2.30533777990687	-0.71912538681576
C	8.01596838251312	-2.20093093176032	0.81849220600952
H	6.12904283251378	-3.17163447357439	0.50819205812093
C	4.81623016720633	-3.66649660219192	3.05614534404128
C	3.10884582709010	-4.81150575467478	1.72503457727373
C	1.86058880093008	-3.66314803695071	-0.04788252599380
H	9.77710133003172	-0.99985376782588	1.24076010879632
O	-2.00898311421972	-0.78211437193147	-0.68244412872936
N	-1.72032619189414	1.62881783354011	0.36545260874989
C	-3.40383958633399	-0.86897992329929	-0.65216964050419
C	-5.42062939204718	-1.63272287244416	0.45217848882530
C	-4.26182590559692	-4.60060118396069	0.56113682078054
H	-1.98214106095394	-6.57478046907169	3.02230197220033
C	-3.56237468073307	-6.69455001784267	1.56913413261903
O	4.71802162173357	1.21207419823025	-0.97637771602875
O	3.28921508419826	2.49457930472286	-2.61124391291108
H	8.58977446276511	-3.03869417984961	0.41973770388224
H	5.39556177338151	-2.77322133539384	3.28224224279698
C	4.98036641499173	-4.80200153792818	3.82241134428259
C	3.31375331378354	-5.96917298595289	2.52264109909220
C	2.12180905537538	-4.79129812060928	0.71350889753619
C	0.77299804442083	-3.67897164960433	-1.05108019845994
S	-2.21428413441242	2.55589772008904	1.57881871197991
C	-4.13637514216856	-0.20407772623302	-1.67758876833894
C	-6.18511385309813	-0.95192271961704	-0.55130072292362
C	-6.10960929306040	-2.23486697957672	1.53599328499364
C	-4.37784875152104	-5.97134473417754	0.66904589091588
H	-4.89329147636368	-4.06050666714644	-0.14243157859079
H	-3.66832396067227	-7.77721210956121	1.65034932984536
H	5.69198513339687	-4.79548929440898	4.64938242202571
C	4.23121346563044	-5.96924830944468	3.54992956185423
H	2.71605953382907	-6.85819216519953	2.31034824454884
H	1.54379926270390	-5.69850588920392	0.52806279606964

C	0.89617625367077	-3.11830087503215	-2.33033326185116
C	-0.43259907643233	-4.33058297955322	-0.74206019429642
O	-1.51521067268930	2.31146065760935	2.83776153795415
O	-2.25641719783406	3.93321540903666	1.07714300838068
C	-5.51845087408266	-0.29246418264634	-1.60821630292401
C	-3.49000932913233	0.48863679187140	-2.81571680876685
C	-7.60204638855603	-0.94792841825606	-0.45806927909117
C	-7.48653522220584	-2.19201545539832	1.61089541229333
H	-5.53555873012170	-2.71610862068618	2.32640167825701
H	-5.10280988739823	-6.50120716351075	0.04919752385469
H	4.37473866951993	-6.86269262912575	4.15914078327146
C	-0.13389202570928	-3.22941884321127	-3.26589662919657
H	1.81622379619941	-2.61025646420236	-2.61299192726008
C	-1.45285004696785	-4.43845656313110	-1.67867513143844
H	-0.58128694478146	-4.73978085793162	0.25762371398631
H	-6.12093929475574	0.16222252202188	-2.39538921072550
C	-4.12044405238251	1.58408436207690	-3.42616505166540
C	-2.29050279242919	0.03093815125459	-3.39348164974818
H	-8.17364134110247	-0.42995711683615	-1.23046195913471
C	-8.24238561836792	-1.55512014842990	0.60044808839948
H	-7.99421603291310	-2.64282906366253	2.46455696514612
F	5.56080218041257	-4.24376709964486	-4.25826922302380
C	-1.32884463229419	-3.89906003121552	-2.96881773116615
H	0.01363781368391	-2.78994935156090	-4.25112016603798
H	-2.36921480286433	-4.95124192076401	-1.38505304013154
C	-3.60333806925184	2.16895788392130	-4.58245243858420
H	-5.02981284080380	1.99618897334817	-2.98507224620686
C	-1.77782176211941	0.62675249990668	-4.54029505209969
H	-1.76359055242103	-0.81821122407320	-2.96282377689506
H	-9.33093506656051	-1.53222415968862	0.66863616932768
C	5.43545355073833	-3.37034015551253	-3.22756689362147
C	-2.45676659128914	-4.09458147506689	-3.98742499497851
C	-2.42356179651829	1.69969580238149	-5.17561093246626
H	-4.13693559910785	3.01342309900230	-5.01794655239361
H	-0.85564411850238	0.22284910591382	-4.96203698581032
F	6.68570540246169	-2.96401918630918	-2.88031503727972
C	4.54529466642790	-2.20010464023711	-3.56990263381994
F	4.93918439794848	-4.06812951484892	-2.16499841696555
C	-2.59897875783402	-5.60554212387563	-4.26851514889958
C	-2.17960729972023	-3.37459816324973	-5.31453650325411
C	-3.78476357915637	-3.56297421421436	-3.41208015641519
C	-1.83627331848684	2.29444220714441	-6.45915926473550
C	3.80143383321217	-2.18556196481909	-4.75113760039412
C	4.46069766104851	-1.14185109327026	-2.66397950834669
H	-3.40856095059740	-5.78239542205578	-4.99297063934477
H	-1.66729955346622	-6.01586073320648	-4.68428728301356
H	-2.83411855309944	-6.16229748086094	-3.35053501064008
H	-3.01282149237231	-3.55448795560691	-6.00910222027046
H	-2.09069188252075	-2.28753889622322	-5.17802796540278
H	-1.26143911670868	-3.74210250991703	-5.79526510809362

H	-4.04660639426559	-4.06085876905840	-2.46863657471345
H	-3.73016194348399	-2.48360727405998	-3.22046481870755
H	-4.60272922585605	-3.74071973840056	-4.12645039312277
C	-0.41197843576210	2.81674901708372	-6.18174893530684
C	-2.68009983666905	3.45672383667946	-6.99914207252969
C	-1.77101264229113	1.19226757539239	-7.53746777400869
C	2.96605451070540	-1.09984536688579	-5.02499068500799
H	3.88423402444337	-3.00687777091957	-5.46050294859605
C	3.59939094442288	-0.08390758094163	-2.94321555568911
H	5.05156616817209	-1.12982439213151	-1.74784312097052
H	-0.42714576824093	3.61344401058690	-5.42523347722996
H	0.24927687998172	2.01747401912573	-5.82032128915206
H	0.03050719810984	3.22481455064785	-7.10259491274845
H	-2.22347159245006	3.84899309917203	-7.91914056626495
H	-3.70323150247087	3.13680398628526	-7.24467610224031
H	-2.74169474673692	4.28588380936249	-6.27862053724984
H	-1.34451858322063	1.59730328925118	-8.46744150384626
H	-1.14352655260759	0.35019734181547	-7.21630144718152
H	-2.77513701550105	0.80270740187039	-7.75888492048372
C	2.84973290577240	-0.04535591131019	-4.11635921134858
C	2.16945072537465	-1.05541033380749	-6.30497706039651
H	2.21210362118764	0.81376024000359	-4.32556278445928
F	2.19409494660943	0.18069151138356	-6.86934309353226
F	0.84933707409241	-1.35383176285533	-6.08516496168647
F	2.62274227526104	-1.93787070151215	-7.22735830149520
C	-4.27656705302918	1.33377771698019	2.95275490352195
C	-3.93256056505192	2.09759541581899	1.84204902914107
H	-3.50111510168555	1.02508237346826	3.65240271295878
C	-5.61534519351090	0.98861935434571	3.15416275792699
C	-4.90220138490929	2.53076966851242	0.93883411112116
C	-6.60117415928380	1.41437261375453	2.26494620579217
C	-5.96915059053332	0.18043048390816	4.37675402805305
C	-6.23716648256193	2.19114276785861	1.16206902753787
H	-4.61361987317589	3.14081782510060	0.08292693315917
H	-7.64162660918397	1.14082921179717	2.42545503608451
F	-7.26830352455223	-0.21430653881554	4.38577597621095
F	-5.75879510957449	0.89342268561976	5.51994849941219
F	-5.20221475714972	-0.94321206221830	4.47621292586540
C	-7.28850871024121	2.73971682405420	0.23174844058326
F	-8.49712068146840	2.14481740211600	0.39665826886612
F	-7.47210440497583	4.07657463801007	0.42703943455850
F	-6.93983395238686	2.58819516612140	-1.08108141285200
H	-1.48141456004660	5.26076619749537	-0.02363302499366
H	-1.78934555496533	6.08745364937670	-2.71228978081882
H	-1.86014672219452	3.66810463089756	-3.27081627938179
H	0.49428643941055	3.10644686428925	-3.03426268068903

228
Major3

C	-0.09054972114119	3.45724128948675	2.77429227922828
C	1.11701017212202	2.71057320407120	3.25633347502460
C	2.21521274793605	3.61721497085614	3.84892731106798
C	3.04351261988366	4.31164568657139	2.77704112161463
O	2.24146762809566	5.18580847733462	1.98323994347971
H	0.79586896591984	1.99437364195739	4.03097304688617
H	2.89118785419362	2.99693456481565	4.45556552556751
H	3.52520071493442	3.54970458424824	2.13982897763834
C	-0.87010321686449	4.24252144397558	3.74039915072988
C	-0.89841968349756	3.90816348019616	5.10743015576355
C	-1.61186431995020	5.35792043912152	3.31071227286450
C	-1.65810810772901	4.64935204197331	6.00678423115690
H	-0.35230766864577	3.03525965160750	5.46365669614651
C	-2.36043982210464	6.10752449871798	4.21258650463636
H	-1.57020184419360	5.65317380379709	2.26363452432315
C	-2.39031879708781	5.75379017326366	5.56369103653146
H	-1.68365667202748	4.36224082538626	7.05892164591594
H	-2.91857785016560	6.97714515708570	3.86168949783296
H	-2.98024528828301	6.33883728847461	6.27104674707091
C	-0.50237001005909	3.32628508500072	1.46516711697299
H	-1.32457862412935	3.93292498519488	1.08169660767706
H	0.21802257305103	2.94682180205457	0.73547574988293
H	-5.09705391754340	1.27770199585611	-4.57938003172493
C	-4.62742095413596	2.25899857454522	-4.72781769082098
C	-3.36770999222419	2.16023999302541	-5.61038042914358
H	-4.38844672666972	2.67343693873316	-3.73920447871667
H	-5.36835704333282	2.91950025986133	-5.20304701475657
C	-2.36369257049078	1.18227426842369	-4.99189350316412
C	-2.78389641524511	3.57125766228464	-5.77025961349456
C	-3.76696295625297	1.62983614442610	-7.00374117852392
C	-3.52219808567600	4.80147856671339	-0.18216800844952
C	-2.75541129333163	-0.13061024915056	-4.68091808531053
C	-1.02794021755510	1.51993358327662	-4.74111386507484
H	-1.91798152178538	3.58323509765241	-6.44802055396411
H	-3.54823805959461	4.23690176358419	-6.19677623157496
H	-2.47425408913427	3.99386018017179	-4.80324970522537
H	-4.48894249549480	2.31201940441386	-7.47811450396354
H	-2.88637941510354	1.54928687536328	-7.65728636928015
H	-4.22973045299619	0.63503978063530	-6.93610851436982
C	-3.28490834386936	3.65826541054625	-0.96957104671078
C	-2.65942431470004	5.89234905290195	-0.21820431438864
H	-4.37902572638632	4.82125094199665	0.49325879190503
C	-1.86199950291186	-1.05348468429007	-4.15131554274530
H	-3.78652790044325	-0.44734409230076	-4.84308271875244
C	-0.12221964724045	0.59557446917904	-4.22189187850996
H	-0.66470641172835	2.52468335454761	-4.95446060602184
C	-4.24789753982348	2.53467415379754	-0.94479937311216
C	-2.13429397952679	3.66210438416759	-1.77600894594938
C	-1.49963650534122	5.89364633248008	-1.01363910615187
H	-2.88818377229950	6.75768969669383	0.40706458313745

C	-0.51632965566582	-0.71659997711596	-3.91907282612415
H	-2.22366967195124	-2.04932354421654	-3.89131116495720
H	0.90153705564010	0.91707975286218	-4.05126164938699
C	-3.85736243267064	1.17614268130014	-1.10907888322507
C	-5.60167464336438	2.76206551727643	-0.75238745922040
C	-1.26137958299911	4.74987903927324	-1.78655930349656
H	-1.90340818337948	2.80761678593774	-2.40836093028668
C	-0.56645162409983	7.10742268385511	-1.02075657391757
C	0.42341617167010	-1.74270016485320	-3.41551126654188
O	-2.48682356923548	0.90516311189933	-1.25509316075391
C	-4.73612447014839	0.10557721877080	-1.13260654395391
H	-5.96060630366738	3.78878515532886	-0.66463134113021
C	-6.53921600052453	1.71147470001810	-0.63639592602315
H	-0.36863764846176	4.67671924653936	-2.40583932751218
C	-0.13078635303982	7.44760028465945	0.41896492294107
C	-1.32915387569291	8.30777933079594	-1.61948539707376
C	0.69463304505210	6.85891231099363	-1.86095342017796
C	1.51834629585100	-1.44344019013590	-2.55640146400635
C	0.27110504321031	-3.07848609619418	-3.75913393505138
P	-1.74946486931595	0.19886950808884	-0.01023273400071
C	-6.11006878032241	0.35461236335851	-0.80728334120754
C	-4.26573117228540	-1.25852983806022	-1.48108677409517
C	-7.90118466579909	1.97297611778528	-0.33144782531178
H	-0.98803511843409	7.71509038289351	1.05335279522293
H	0.40065519234507	6.61036721383631	0.89252256347457
H	0.55019031453868	8.31163167346504	0.40748561147531
H	-1.64538856655150	8.09472184286638	-2.65103239186069
H	-2.22758302579332	8.55101155151067	-1.03378815778470
H	-0.68212296871150	9.19796505828249	-1.63394765108324
H	1.27714611186922	6.00531413610826	-1.48741470067445
H	0.44965735675203	6.67218596660601	-2.91670294021313
H	1.33972336347720	7.74858221643690	-1.82272688258503
O	1.70421650779930	-0.11969268844788	-2.13710559949715
C	2.42854923395340	-2.37696563297271	-2.08879841219768
H	-0.53839557626577	-3.36300847670661	-4.43341999496285
C	1.12669798835600	-4.09432317729048	-3.27587485340125
O	-2.55978062646820	-1.17546870460385	0.21925710548947
N	-0.28419680768967	-0.00911452034784	-0.45313013348357
N	-1.91167844033463	0.96856730529141	1.42091250620685
C	-7.05182355001917	-0.68477842371106	-0.59453996959209
C	-4.89714972212082	-1.98452806311079	-2.54829797787407
C	-3.21274944401318	-1.86650630329322	-0.81082046257640
H	-8.21858305957224	3.01159522811071	-0.21769495611097
C	-8.79750902644160	0.94202176692351	-0.15424018269341
P	1.25349587657931	0.23804240158635	-0.62289723457551
C	3.53291081051387	-1.94119060797954	-1.19323495186838
C	2.23366906283924	-3.75347924157113	-2.42907202221277
C	0.90646339203978	-5.45747671329183	-3.60835049209271
S	-3.11704667141920	0.81779851902122	2.55614263612036
H	-1.20129828254423	2.08538676842918	1.58713617072930

C	-8.36185032715569	-0.39761054221529	-0.27028509663284
H	-6.72830376846141	-1.72194919114033	-0.67670256559968
C	-5.86513496760985	-1.40008887516395	-3.40726446424513
C	-4.50243039266586	-3.33926293639407	-2.79860930962579
C	-2.83188062599125	-3.22381672013412	-1.00891086772429
H	-9.83835506062872	1.15550012237279	0.09294849936026
O	1.90907128646611	-0.95672991605713	0.26854135932857
N	1.90486607420570	1.63439067476241	-0.22562840036046
C	3.24366745711400	-1.29506758378419	0.00220033966736
C	4.90619817004877	-2.15363127776647	-1.53744547936000
C	3.05422146644544	-4.79845936528958	-1.93246534177559
H	0.06191270271992	-5.70440442748964	-4.25521806140534
C	1.73104554128666	-6.44807119610185	-3.12259360754419
O	-4.44338746446900	0.98257720022453	1.98923858434433
O	-2.67496497918361	1.65477170750957	3.66018524343367
H	-9.06580650821429	-1.21283770301929	-0.09690588460285
H	-6.15899714197467	-0.36428175592127	-3.24738963634860
C	-6.42586949679526	-2.11858571773105	-4.44274439387936
C	-5.10947700444573	-4.05727655742804	-3.86350729800706
C	-3.50208677164713	-3.92971328188371	-1.99390132698225
C	-1.80755574325207	-3.91049704852184	-0.18934980198249
S	2.42743895774150	2.74141971952856	-1.26605422131895
C	4.22334591319861	-0.98463200860494	0.98650006472250
C	5.91989777396843	-1.80002864341081	-0.58674314827561
C	5.30734787115782	-2.64958582797565	-2.80436794978536
C	2.81185713565598	-6.11247389905253	-2.27532788249250
H	3.88435858805649	-4.55352258690535	-1.27099891776110
H	1.54881243434602	-7.49169920724273	-3.38275917042058
H	-7.15937731027449	-1.64111564364534	-5.09413790908016
C	-6.05374858744259	-3.46294584956297	-4.67094763272932
H	-4.80110686443053	-5.09068848623610	-4.03548503464944
H	-3.22104701318822	-4.96846255019719	-2.17805558791549
C	-0.52728967867422	-3.38445172320792	0.01388282569307
C	-2.09067335965470	-5.17553213912653	0.35437241948003
O	1.57887712187783	2.89752860182772	-2.44454152468339
O	2.74739505144666	3.94475337558845	-0.49160397781464
C	5.54272231465004	-1.26231494276960	0.66456271564026
C	3.88410686509384	-0.46431961673700	2.32942081580072
C	7.28471661533015	-1.99476120375101	-0.92556404039757
C	6.64326036934941	-2.80662671917982	-3.11062173970138
H	4.54604535127187	-2.88661296020469	-3.54649186389901
H	3.45682877589861	-6.90035679992148	-1.88328324199525
H	-6.50804348804510	-4.02232601812982	-5.48986196546329
C	0.44351563816294	-4.10931652708967	0.70344953711903
H	-0.26609725214139	-2.41514531003709	-0.40346345691260
C	-1.11831172849038	-5.88819975732052	1.04871927051383
H	-3.09099335717826	-5.59665508349915	0.23950721795682
H	6.32061461688266	-1.06326835460976	1.40320140608641
C	4.72396447995471	0.46292643135166	2.96385814132214
C	2.79656659821282	-0.96689493125616	3.06536254630923

H	8.04761938717069	-1.72448677512290	-0.19306920001479
C	7.64177126494901	-2.48617607539046	-2.16261020980311
H	6.93127814107979	-3.16854245773463	-4.09841657485357
F	-0.80334816376812	-4.29176211567287	5.60186826379190
C	0.17993118962365	-5.38088528963318	1.22750841481169
H	1.43342442350778	-3.66773153047356	0.80366775974758
H	-1.37791338663191	-6.86885413034825	1.45195331115667
C	4.52032806222554	0.83261423649723	4.29384231854677
H	5.55658282761908	0.89814742124229	2.40921443814606
C	2.60195604150692	-0.59289918645172	4.39061185987556
H	2.11618908895223	-1.68510870059224	2.60919597368159
H	8.69440032009799	-2.61892938813342	-2.41664206757779
C	-0.60266236215786	-3.08930281834902	5.00866336863888
C	1.24933697252068	-6.23244966200821	1.91980331141691
C	3.46647266434178	0.29863241944651	5.04720043264421
H	5.20813019209369	1.54866074820413	4.74308442425575
H	1.77038493762627	-1.03668124745592	4.93764905310792
F	0.52930479526216	-3.19906903152835	4.25205214231709
C	-1.78425106995444	-2.64629780269221	4.18681988487263
F	-0.31785159187533	-2.19270570907721	5.99826733884192
C	2.58037403997260	-5.48038846868433	2.06534461970709
C	1.49052338618648	-7.49829078666208	1.07001255245836
C	0.77111508824189	-6.65355797405645	3.32315009104522
C	3.26649871216754	0.59881884582534	6.53699856148662
C	-2.75543691236721	-3.56939988640264	3.79236347486152
C	-1.88494947965911	-1.30409673730844	3.81828495296419
H	2.46792679447656	-4.56706737132908	2.66719019283710
H	3.31470324337550	-6.12484012441968	2.56965260660851
H	2.99694654150957	-5.20015112403838	1.08731419400407
H	2.27190958757720	-8.12069994969369	1.53218546956631
H	0.57800364677541	-8.10541411833359	0.98720788506140
H	1.81112864620459	-7.22995352394672	0.05405521474074
H	0.63557855860198	-5.77877964485914	3.97124411905530
H	-0.18065616980141	-7.20147060876850	3.28329326085808
H	1.51649158343328	-7.31332654557058	3.79235241043680
C	4.23067246941098	1.67791902183075	7.04874789754356
C	3.52121911955813	-0.70327990873296	7.32695571060072
C	1.82422645562404	1.07279725655837	6.80443636189575
C	-3.85694406786399	-3.13261822099384	3.05661555073671
H	-2.65767957760110	-4.61859866935709	4.06354375759548
C	-2.98580488305745	-0.89508819292482	3.07062653067911
H	-1.12457585286147	-0.58005501805057	4.11132570405984
H	5.28098720340925	1.36762387301888	6.95172989442009
H	4.10020687494605	2.62956740234631	6.51251843788486
H	4.03815113380262	1.86668333084599	8.11457608393569
H	3.38904717890301	-0.52797488551395	8.40545562818633
H	2.82574829305822	-1.49742391005620	7.02193777046091
H	4.54521696776441	-1.06605663247545	7.15812574227389
H	1.61413548110682	2.01190856666707	6.27290469861637
H	1.08195815752470	0.32434879145532	6.49527873599466

H	1.68298791002205	1.25536488592951	7.88017328713255
C	-3.98656922355776	-1.78893177837724	2.70210190458304
C	-4.94488569079480	-4.09443347166044	2.64810519641400
H	-4.84080858944209	-1.43057910680117	2.12765078307573
F	-6.09037190872256	-3.87552754310111	3.35332486294468
F	-4.59341689709412	-5.39102178602480	2.84658299897817
F	-5.26634669460206	-3.95817061044609	1.33058210876711
C	5.12785134781143	2.18992943841669	-1.01976561091397
C	4.00950690496484	2.11251702943991	-1.84907588114046
H	5.04303420608060	2.64553277128103	-0.03332359561618
C	6.34915041946568	1.69458422845845	-1.47869887767702
C	4.09970894195462	1.55585333505031	-3.12116917383103
C	6.45382310036780	1.11189211552558	-2.74428658766589
C	7.57391681518393	1.86121622160933	-0.61649971657402
C	5.32496815882745	1.04723558391930	-3.55941962327499
H	3.21773441942986	1.52457109043766	-3.75870660758941
H	7.40437615739466	0.70930902871619	-3.08724812070629
F	8.02453367592874	3.14729057969218	-0.63892427176856
F	8.60511678800915	1.07225696426588	-1.01371577922266
F	7.32267515631253	1.56413918211735	0.69354578919425
C	5.40344931303592	0.46494752772638	-4.94821121330324
F	4.43470600558910	-0.47129187638000	-5.15727666242169
F	5.22358681725420	1.42680098300839	-5.89828248471950
F	6.59944530489844	-0.12606182279771	-5.20003314458200
H	2.30132797435400	4.85597345618285	1.06188270952552
H	3.84621573466757	4.89761496273241	3.26178257226417
H	1.76886709116513	4.37113243860434	4.51500112408398
H	1.53553038265443	2.11656472579414	2.43145573853814

228
Major2

C	-1.18107351519381	4.33134819791149	0.28843096117256
C	-0.19448269347373	4.68839680712555	1.35885999758153
C	1.28873335152467	4.67304300855516	0.98107208799469
C	1.64732128727465	5.84108971586546	0.06972433343125
O	0.83456868189270	5.89828914360175	-1.10170829059460
H	-0.46584994645980	5.66123001647718	1.79435427269587
H	1.56086596994021	3.71844480124675	0.50882430777701
H	2.71940080481480	5.79139857968981	-0.19819996203422
C	-2.40208199503392	5.14275799084936	0.18210355740370
C	-3.64712067372956	4.52813844403955	-0.03267875198559
C	-2.34827943125385	6.54555058922028	0.27030569393935
C	-4.81043370305300	5.28597825591644	-0.12558239784987
H	-3.69066054916876	3.44296933577120	-0.11461543540801
C	-3.50786665114441	7.30586164764793	0.13581958518286
H	-1.38293962069674	7.03830258319649	0.38557937594876
C	-4.74378674738047	6.67963020750741	-0.04615759018416

H	-5.77245511750960	4.78844695510845	-0.26109984621887
H	-3.44753569369201	8.39427637860924	0.17817571432283
H	-5.65323890657165	7.27680931126602	-0.12776281878326
C	-1.03542255870780	3.19731716355198	-0.49238640180342
H	-1.68229557934329	3.08324367101513	-1.36468193469476
H	-0.03679719346017	2.76005517966268	-0.57681536851167
H	-4.72677332600623	-2.38675156375687	-5.31389241881675
C	-3.86178187304042	-2.45178345858245	-4.63666566232877
C	-2.55561978754467	-2.52884671327476	-5.45207054715957
H	-3.99554279720008	-3.33765555063877	-4.00137792878510
H	-3.87081737742661	-1.56657046439482	-3.98730690262839
C	-1.35880123510870	-2.67155467510883	-4.50586608112946
C	-2.46220972074770	-1.27487217547563	-6.33221634530796
C	-2.60356382502421	-3.77082694321673	-6.36695909175597
C	-2.59556996205424	1.09687832795350	-3.19180428236266
C	-0.25293492369955	-1.81227015862689	-4.53433639528130
C	-1.32685970706210	-3.72128398258901	-3.57255760606097
H	-1.57477196668299	-1.29479728061404	-6.98135705513407
H	-3.34705907981789	-1.21848617933137	-6.98277538173481
H	-2.43336831449543	-0.35510026577215	-5.73046758623135
H	-3.45719190684504	-3.70333746196419	-7.05885345372134
H	-1.68268009884676	-3.85292408562573	-6.96228226515610
H	-2.71080615252090	-4.69498163969827	-5.78156255123317
C	-3.80808664032933	1.14588346439699	-2.47470524277406
C	-2.11974661591624	2.21564975498616	-3.86610429311960
H	-2.00881988738520	0.18088259695204	-3.22069489324964
C	0.83815662775820	-1.99122697443036	-3.68314372896419
H	-0.22291961310798	-0.97713474915570	-5.23374582085913
C	-0.24487553728990	-3.90586468738785	-2.72056207103171
H	-2.17075992073694	-4.40747723075994	-3.49375563430961
C	-4.35554214211843	-0.01507954646848	-1.73756582568465
C	-4.52226147692221	2.35265175649841	-2.50005761731572
C	-2.81875500337872	3.43605194475462	-3.86631293009596
H	-1.15894409659685	2.13711671044371	-4.37759034991894
C	0.86873610906557	-3.04859962982179	-2.76171893267912
H	1.66572281687523	-1.28817860120119	-3.74241676284758
H	-0.27624125523696	-4.71176978220142	-1.98630048810507
C	-3.52286097435629	-0.96192262539175	-1.07513842993131
C	-5.72182507182935	-0.23999628681843	-1.67413951620787
C	-4.03954463578891	3.46910285267986	-3.18138566378723
H	-5.45586471460976	2.43469429434383	-1.94106401320480
C	-2.22439022552709	4.66152238993043	-4.56259303907524
C	2.01868218134635	-3.30734592904367	-1.86638111050643
O	-2.14629260033896	-0.73184118959907	-1.10959312410754
C	-3.99259616799448	-2.12067522019370	-0.46950298900692
H	-6.39755565099398	0.43921782209537	-2.19573161766524
C	-6.27771855538026	-1.31893929819023	-0.95056167886119
H	-4.61809013598969	4.39056412648218	-3.13908298652878
C	-3.14133077191913	5.88815226388083	-4.45644994879550
C	-1.99992071836572	4.33919923291096	-6.05383322381485

C	-0.87579375230787	5.00726103340793	-3.89915056781027
C	2.74712691401963	-2.26631834021711	-1.22512570868697
C	2.42694338011691	-4.60341815933765	-1.59387215943975
P	-1.33069425611623	-0.43468887638760	0.25021658501502
C	-5.41052641190473	-2.28172068669951	-0.33520516867621
C	-3.04107317917205	-3.16500859681345	-0.00424426662721
C	-7.68345767169362	-1.46686810465105	-0.81469241727275
H	-3.30432939720026	6.18312089044133	-3.40883336322907
H	-2.67368144097926	6.73941135790078	-4.97145434610611
H	-4.12010851264529	5.70984524113477	-4.92634476929538
H	-1.30904930123769	3.49522594605454	-6.18575452698444
H	-2.94882009621583	4.08247995667689	-6.54734540412803
H	-1.56797026742648	5.21125984940558	-6.56709137586999
H	-0.41021449474417	5.86296079445104	-4.41072398017774
H	-1.01869001313417	5.28477928778457	-2.84554848060398
H	-0.16938683891203	4.16654020984324	-3.93634194478258
O	2.37399214434575	-0.94936993574858	-1.50151229187227
C	3.81332019128662	-2.47000268115878	-0.36067771985161
H	1.91743622507500	-5.43413050688742	-2.08583375997396
C	3.48182242323572	-4.89298207128399	-0.69783077542559
O	-1.91566868136448	-1.51007832191740	1.29682663509647
N	0.14513580866831	-0.67090560344572	-0.14742067712596
N	-1.80294647285111	0.99652011313598	0.90446255936316
C	-5.99330073430792	-3.33149010022440	0.42015303789665
C	-3.11127031518034	-4.50997702905187	-0.50024123334212
C	-2.00916607313465	-2.84438153928338	0.86339333421648
H	-8.33263555794017	-0.72782881779171	-1.28820941936239
C	-8.21821392893677	-2.50225308709079	-0.07912332331934
P	1.59135255871646	-0.09491798758186	-0.37114850748856
C	4.48963933837769	-1.31312327528680	0.28744240661385
C	4.18637731279644	-3.82104827036638	-0.05338084691494
C	3.83687126739129	-6.23587285175495	-0.39870953430955
S	-2.29841049469482	1.23831635940969	2.46182500218405
H	-1.48405508875922	2.13856918080323	0.29324393295333
C	-7.36289116473953	-3.43602997924864	0.54898471546087
H	-5.34373898148904	-4.04909018999136	0.91924315837155
C	-4.04676452029881	-4.92648664407308	-1.48295289783922
C	-2.15513993566667	-5.46835014715002	-0.02344232143214
C	-1.04408667963513	-3.76719152605410	1.35179994262046
H	-9.29965049010451	-2.59750448234767	0.02812647842019
O	2.35403547453503	-0.51363968700410	0.99269631058516
N	1.79133545957756	1.44834236950886	-0.70983536478393
C	3.74587836370489	-0.38962660816750	1.01218466479705
C	5.90673572446352	-1.12316124837953	0.20365383829791
C	5.18492774261300	-4.15359287281966	0.89964768907164
H	3.29929890844388	-7.03958382039945	-0.90654670864943
C	4.82290059624647	-6.52263867770251	0.51939827669367
O	-2.38444622212854	2.68443638613515	2.60918200189187
O	-1.52371244939040	0.46002573948867	3.41467009971034
H	-7.78779793309168	-4.23938489952648	1.15202576388170

H	-4.75692205857074	-4.20361598809009	-1.88018591645936
C	-4.05779268354464	-6.22427149224826	-1.95130036688810
C	-2.20554118920163	-6.80084124481914	-0.51373915803627
C	-1.16071547005671	-5.07216911603216	0.90028314135256
C	0.03895586217113	-3.38424812946825	2.28365250671248
S	2.20383621273437	2.03222583879418	-2.15080569016941
C	4.31179222273529	0.64522374499234	1.81310677467823
C	6.50689090683652	-0.07766292632243	0.97995956557086
C	6.73564035371105	-1.89745119919499	-0.64790635247890
C	5.49426207046164	-5.46871548178103	1.17999393710504
H	5.70717807440465	-3.35381337859354	1.42156908907085
H	5.08117130204775	-7.55839452328602	0.74412113638380
H	-4.78157194094171	-6.51587519855157	-2.71388548922457
C	-3.13629916514655	-7.17552113890368	-1.45751291249542
H	-1.47793264042282	-7.52114891386959	-0.13381234725539
H	-0.45814648375994	-5.82296255504626	1.26661958037470
C	-0.16541397046202	-2.51814215459405	3.36656479871838
C	1.32190722017738	-3.93404058510215	2.12335063849433
O	1.59683461443824	1.32574519169393	-3.27602365706459
O	2.03428408686848	3.48645440393337	-2.08842709818675
C	5.69531192582975	0.74715197619101	1.79099103823311
C	3.50002013405250	1.53944542238772	2.67287640869855
C	7.91225454758961	0.11443978824283	0.90991249420544
C	8.09507695328634	-1.67063437726835	-0.70969979739235
H	6.28166303571248	-2.66531663864668	-1.27255047551121
H	6.26154577806739	-5.69569471035106	1.92178838963654
H	-3.15889517866736	-8.20008775806714	-1.83122701846596
C	0.86758522536333	-2.22428091436014	4.25626049661184
H	-1.13795362985510	-2.05854044939149	3.52554345318320
C	2.34326178898603	-3.64484094233952	3.02022105168414
H	1.52787379386388	-4.57956376460533	1.26841625387076
H	6.18606740988947	1.49065277230178	2.41990599115319
C	2.28429471931436	1.14111208215016	3.25534395717816
C	3.97049751632954	2.82667729708875	2.99590182294438
H	8.35927007667902	0.91130611901888	1.50729245624170
C	8.69259902762621	-0.66456848585184	0.08333281027988
H	8.71079337779262	-2.26384910267102	-1.38694427765220
F	-7.65775600480476	2.81341884673632	2.14111793005212
C	2.14368999372171	-2.78428125815265	4.11238069549814
H	0.65565427880127	-1.54221915049257	5.07896848004495
H	3.32053587996952	-4.09790166128291	2.85136817040043
C	1.57971707816745	1.98515002063866	4.11307491984963
H	1.87565502240612	0.15343734388199	3.05913031052027
C	3.27453615515791	3.65234897408047	3.87250033754042
H	4.88938316682291	3.19656224489580	2.53826052611069
H	9.76916073328787	-0.49690497714798	0.02824620932146
C	-7.40683039501934	1.71700110758374	1.37188161031770
C	3.28269453749607	-2.52896993747966	5.10508975576332
C	2.05679529539724	3.25646751162522	4.45363578149352
H	0.63488306981402	1.62128512365023	4.51488006008221

H	3.68834213739730	4.63685652083860	4.09798736186896
F	-7.07708562525969	2.18642838209911	0.13041658847190
C	-6.30442042487693	0.87375480856073	1.96190175684196
F	-8.57397276885515	1.03926087288654	1.24749572994597
C	3.57444816288380	-3.84812159355395	5.85160942234940
C	2.93055487533048	-1.44656156117115	6.13550798432569
C	4.55273362628657	-2.08375491497403	4.35428403240956
C	1.33188564871036	4.16782830104256	5.44910922652004
C	-6.59612853854447	-0.33368613704197	2.60137225782948
C	-4.99599532739070	1.35478912073122	1.91135572228486
H	2.68832205298342	-4.18765210610614	6.40732223231379
H	3.86026723775656	-4.64602206138567	5.15165233201063
H	4.39918680594741	-3.70804787452472	6.56719315883214
H	2.07099643794366	-1.73536305168109	6.75787666145913
H	3.78680659537362	-1.29097331370912	6.80779778647458
H	2.70455786490461	-0.48425256232090	5.65339268353727
H	4.88027227876055	-2.83850054991851	3.62703072319692
H	4.38510289369445	-1.14343618134342	3.81350143681282
H	5.37509158958358	-1.92504134441855	5.06793038700470
C	-0.06789016121258	3.64127228968162	5.79920209365230
C	1.18102663133573	5.59068765806511	4.87572582135346
C	2.17598010166401	4.23469257455774	6.74012298321453
C	-5.56457661634521	-1.05555732204146	3.20092454637963
H	-7.61709605498129	-0.70836998261560	2.62982456161779
C	-3.97717301611538	0.60648480651054	2.49900540197902
H	-4.76893660650711	2.31075963976851	1.44042985935988
H	-0.56464421005528	4.34700162103367	6.48062417265797
H	-0.70043934247801	3.53028449699260	4.90638289447892
H	-0.02346120743836	2.66741657065502	6.30731110188276
H	0.56977656594961	5.58687425094115	3.96289653746993
H	0.68543405177648	6.23936702543608	5.61324507668349
H	2.15219394685398	6.04423884905553	4.63464708497566
H	3.17593254252964	4.64563514145030	6.54090720347346
H	1.68342245972308	4.87724446025505	7.48590653939543
H	2.30191091808506	3.23286716860281	7.17527377260477
C	-4.24792558209915	-0.59170554113684	3.15227534531539
C	-5.84772064834731	-2.32458788316135	3.96682669051847
H	-3.43703207610990	-1.14455985235825	3.62387108499199
F	-7.12247721611695	-2.75934295848517	3.80483751404225
F	-5.65320668437059	-2.13975348049972	5.30380311357588
F	-5.01826831918272	-3.33774596403742	3.59060476384035
C	4.48897442359654	0.77327299823738	-3.07719277108630
C	3.97973550748993	1.77307324179937	-2.25498389769896
H	3.80626116608802	0.15564448625909	-3.65894589849333
C	5.87235646604262	0.58842262644232	-3.14693026219326
C	4.82693130788717	2.59596786860648	-1.51388770643049
C	6.73706488131774	1.40004253164205	-2.41369272509512
C	6.40684388502599	-0.47713602754796	-4.06972471718985
C	6.20634668798028	2.40649456087145	-1.60293945098556
H	4.40758393442307	3.38323755348961	-0.88740853041484

H	7.81267573383692	1.24701159129492	-2.46686531132344
F	6.19655208444699	-0.14965081918840	-5.37714921936069
F	5.78932115709901	-1.67621831931594	-3.86810584446008
F	7.74123270727545	-0.67873201142122	-3.91954202066206
C	7.11596861421761	3.35361057417706	-0.86305946109047
F	8.39662086999115	2.90857459208318	-0.79816141198096
F	7.14952666886840	4.57755919856071	-1.46386795450666
F	6.69671424050186	3.56689052420614	0.41936980576990
H	1.05723825734420	5.09535409482379	-1.61944984535021
H	1.48424039689653	6.79587350821046	0.59683990516612
H	1.88751100752600	4.73821041582766	1.90167439833743
H	-0.37415531049491	3.95083745873855	2.16241530093288

228

Minor0

C	0.16368008231994	-4.39100575019458	-0.74066650816382
C	0.55644303303982	-5.67987709972790	-1.39457878168619
C	-0.68570742798813	-6.50776421071276	-1.80991991205445
C	-1.67246186559458	-5.74604738511730	-2.68837943162214
O	-2.38879748842436	-4.79469636232902	-1.89991693900367
H	1.15573172553289	-5.45693931505936	-2.28919145987365
H	-1.22286918164959	-6.85451571875080	-0.91350166006187
H	-2.38214427309999	-6.46342286141596	-3.14109471079965
C	-0.34897568686094	-4.40581978229382	0.63166549818400
C	-0.19660396667881	-5.53395421454186	1.46465645875869
C	-0.92216952570991	-3.25053818896625	1.19135691084291
C	-0.57318335052161	-5.48752526777269	2.80282041464058
H	0.25800665553199	-6.44423610884316	1.07641602668745
C	-1.29771421746290	-3.20086059070024	2.52585879700572
H	-1.08959265826628	-2.38064036327388	0.56561269399438
C	-1.11556940583684	-4.31684195325291	3.34205249887312
H	-0.43113434886021	-6.36596696965055	3.43388322588249
H	-1.73365738671932	-2.28302384492874	2.91834454236440
H	-1.40102019637445	-4.27823841165605	4.39480731839982
C	0.37216531501778	-3.20541837943153	-1.42738208888423
H	0.75591540772508	-3.25934598080108	-2.44774624292144
H	-0.28541488469848	-2.35163078094618	-1.22881662145718
H	5.29780992652240	2.34786086187297	-5.49572637910433
C	4.58789796813458	2.38401699019772	-4.65568182746869
C	3.32163927078007	3.15767916143612	-5.07587377496400
H	5.09911698142765	2.85689517622928	-3.80570704036106
H	4.34011538061421	1.35284713819137	-4.37089448021938
C	2.34873881024822	3.25802114523027	-3.89686955695662
C	2.69910550761778	2.43415078952266	-6.27849385183966
C	3.71773398237089	4.58546621301967	-5.50686423263426
C	3.92443212461158	-2.11921002747121	-3.24338165165577
C	1.00618380714362	2.86696843248094	-3.98115182773277
C	2.77887146983099	3.78032926492273	-2.66551621997249

H	1.82386296953743	2.97180288120973	-6.67064435801954
H	3.43805981533462	2.36700576962190	-7.08999174859684
H	2.39281650453742	1.41061240697594	-6.01715682501650
H	2.83448942515355	5.15693070524032	-5.82689638452549
H	4.19511908430233	5.13448339681098	-4.68294963556129
H	4.42757738087081	4.54577117290012	-6.34713686574425
C	3.45611248141156	-0.86653929453837	-2.81885281468888
C	3.23765731789557	-2.85623573950891	-4.20911472671812
H	4.83174651961353	-2.53475823454249	-2.80019006634938
C	0.13825990322779	2.97905927782750	-2.89522372834650
H	0.61383941854680	2.44668043933367	-4.90636397487879
C	1.91810080579091	3.90104554542941	-1.58210161124161
H	3.81608965237856	4.09677083398879	-2.54042813143780
C	4.22237869275171	-0.08226411502847	-1.82590029596905
C	2.26910332727676	-0.38567259332354	-3.39701298224320
C	2.06071943045132	-2.37281999592120	-4.79999970847519
H	3.63433452531252	-3.82999985895362	-4.49571694264901
C	0.57465827361633	3.49871270766636	-1.66715316636554
H	-0.89115589712920	2.65267794403705	-3.01829779471888
H	2.30590363364805	4.29171368247683	-0.64298244827301
C	3.59195063025515	0.65328127494453	-0.78478950064102
C	5.60540333548403	-0.03191108973487	-1.87301313093614
C	1.59834680988688	-1.11926178249235	-4.36855661039541
H	1.86832863545601	0.58145189162375	-3.10302876412798
C	1.31185684886362	-3.13050945680037	-5.90033424299086
C	-0.33376997857052	3.69907256740421	-0.51602255748026
O	2.18975619599078	0.63436119863670	-0.73941750652615
C	4.27101410105600	1.38384161609610	0.18204709959555
H	6.12902535579125	-0.54568898353642	-2.68058308785106
C	6.36797524535549	0.65186971943281	-0.89903590276198
H	0.67757643986452	-0.69970126753006	-4.77311625112174
C	1.76033641270407	-4.59590922176892	-5.99959101324119
C	1.59874737405211	-2.42688229019073	-7.24437631939885
C	-0.20563557208274	-3.10661583287336	-5.63696896564970
C	-1.44287229537119	2.84770909626920	-0.24126458690640
C	-0.16701380784770	4.77927706031080	0.33668491846879
P	1.52484088041668	-0.27568687945401	0.41546874779517
C	5.70514918615236	1.34639692337413	0.16773657700889
C	3.51550404508478	2.17079142242170	1.19358984472147
C	7.78731640291195	0.63762261906175	-0.94004539343998
H	1.61088166444924	-5.12738956681475	-5.04713863257842
H	1.16729073960133	-5.11123519518503	-6.76856742505679
H	2.81861302150010	-4.68920180072841	-6.28274070444190
H	1.06510840169475	-2.93348793314489	-8.06305984837807
H	1.26696702525210	-1.37945038523918	-7.21484491797295
H	2.67444960528775	-2.43710020028419	-7.47313923846550
H	-0.44897937760040	-3.54228790700973	-4.65828400400324
H	-0.62120758117294	-2.09139157951854	-5.64779841067781
H	-0.72757792941940	-3.68927493807706	-6.41052798605798
O	-1.57594260039434	1.68042258557040	-1.00578311500566

C	-2.41903564320229	3.11431462410799	0.70574396201400
H	0.65979680228165	5.47044239414027	0.16662352618961
C	-1.03658811126017	5.02805835988551	1.42268093207361
O	2.28422111426985	0.19968330477681	1.75403231612363
N	0.00480768405972	0.02868170107190	0.43635703928375
N	2.01990661366730	-1.82673315822820	0.23941943342029
C	6.50077785349766	1.93374377745691	1.18504795329420
C	3.74333561050279	3.57632246869973	1.37128841860240
C	2.51881863708105	1.56954246815386	1.95203709654168
H	8.27799377128529	0.11090001841351	-1.76055469072328
C	8.53059352746028	1.24560728757930	0.04821108741187
P	-1.38266259987369	0.24773308242191	-0.27611111271764
C	-3.65845010087538	2.29590395506103	0.78012250953838
C	-2.19893039549175	4.20802325587072	1.60695668865474
C	-0.77993425635893	6.08310101553212	2.33869956890723
S	2.73253884553753	-2.77433788867552	1.39647111314233
H	1.32952180981895	-2.57393563493548	-0.62349034102178
C	7.87847614564462	1.88508858751800	1.12637341628024
H	6.01163792976013	2.41636984884772	2.02953397936450
C	4.62817467492208	4.32910780836161	0.55409234188090
C	2.99644671362773	4.27068273149529	2.37960627956424
C	1.73509791280410	2.23763211941569	2.93546522483826
H	9.62047914367269	1.21786194629256	0.01038802878976
O	-2.36739387186798	0.29049205335163	1.01430395176258
N	-1.82606472824452	-0.87617135846455	-1.30289112485890
C	-3.61473076138479	0.91734836331464	0.95437551210717
C	-4.94073958361049	2.93397246708795	0.67879501867850
C	-3.04318898011943	4.47358965559423	2.71623016643143
H	0.10877252438089	6.69823859985202	2.18267974703531
C	-1.61857385952010	6.31263608192948	3.40788301677318
O	2.77881079297102	-4.10331253991932	0.80470628742438
O	2.14435064144481	-2.57138089410309	2.70996374613485
H	8.46736766319376	2.33192904577617	1.92829475357677
H	5.17255096052095	3.82614910959399	-0.24349852463922
C	4.79103776093373	5.68587784518577	0.74256703288367
C	3.20591134735052	5.66431410055908	2.56374392448459
C	2.03336157853594	3.57650048929088	3.14354183126140
C	0.61147626047352	1.60152442910891	3.66168389169047
S	-2.34098773794871	-0.81928227542104	-2.81708457757523
C	-4.77513703016702	0.12783081231290	1.21376519949818
C	-6.11967258098723	2.16060516379090	0.93188914536562
C	-5.09534914783883	4.29235769285318	0.29806942859047
C	-2.75716414596347	5.49562251943804	3.59769289872322
H	-3.91353676477173	3.83969574335658	2.87958716818382
H	-1.40563235780350	7.11962009265583	4.11049928124045
H	5.46807185458331	6.24301591898285	0.09341949185396
C	4.08453897820589	6.36143092026130	1.76444426099722
H	2.63914899129534	6.17321336270071	3.34632379032550
H	1.48548063913448	4.12849918411985	3.90849836688734
C	0.58022058108188	0.25248865007009	4.05188809500836

C	-0.50746386752059	2.39003333460876	3.98302274140222
O	-1.46552270561042	-0.04923809839088	-3.69590924177209
O	-2.66195139321370	-2.19979280906517	-3.19629042013126
C	-5.99427471228631	0.78632266335788	1.23193181952592
C	-4.75166581510952	-1.33186130391883	1.47148454929170
C	-7.39360659230026	2.77976613611914	0.83459295612820
C	-6.34761066717614	4.86170719005756	0.19663142713451
H	-4.21114222192148	4.88134541037237	0.05992480478898
H	-3.41174707304105	5.67009178577259	4.45292769038344
H	4.22910679107051	7.43300847183618	1.90881975746885
C	-0.52964186366691	-0.27648661880908	4.71180357528025
H	1.41514529213765	-0.40995214731084	3.83523563402338
C	-1.60574375982999	1.85454260376911	4.64196599121312
H	-0.53990556968103	3.43442184029134	3.67487963755155
H	-6.89924170207987	0.20478383340460	1.41574170934656
C	-5.49481668215521	-1.86520564484561	2.53970672518252
C	-4.09809569908753	-2.23359580594523	0.62482845308495
H	-8.28138807641202	2.17440989147679	1.02730384722300
C	-7.50836735835983	4.10542456232607	0.47663814148647
H	-6.44252623716310	5.90139230808031	-0.11935457683346
F	6.57078727517547	-1.18171074980335	5.11155655372288
C	-1.65524384378369	0.50239586892632	5.01387237069018
H	-0.50183731915395	-1.33153230795747	4.98249976195657
H	-2.45039773334400	2.51209187288687	4.84594819509727
C	-5.57637390149862	-3.23989262616310	2.73729983581331
H	-6.00434917599963	-1.19058566615582	3.23006754042509
C	-4.19820106854975	-3.61143376775075	0.81602320118304
H	-3.50319725796668	-1.86155350704565	-0.20633495677440
H	-8.49237533454244	4.56911032793301	0.39381260171112
C	6.62200282350322	-0.39886878381210	3.99591835677830
C	-2.87529351561981	-0.04893137053562	5.75896439070759
C	-4.93724941341520	-4.14829067969717	1.87608700918277
H	-6.15584897846764	-3.61113485633075	3.58476713320647
H	-3.67390446715552	-4.26177137193095	0.11577620278114
F	7.89353955101307	0.06789518031075	3.89774574597608
C	6.19174677237859	-1.16777791997813	2.77162257499539
F	5.81143740011273	0.66975669141896	4.23252036842958
C	-2.97016847240575	-1.58023773222101	5.67111649781300
C	-4.17575125377389	0.53880458122659	5.17598534588798
C	-2.75537237727183	0.36113364587842	7.24216421315561
C	-5.07425886379457	-5.65598584306082	2.10516475498509
C	7.13566157647842	-1.63943759398126	1.85893992793153
C	4.83632419592683	-1.46034850362545	2.60865121554428
H	-2.09597382977830	-2.07628182839086	6.11570740381093
H	-3.85694851996686	-1.92376379647988	6.22355661566849
H	-3.07782778117279	-1.91789912007546	4.63079811255845
H	-4.25520886081931	1.62146180342855	5.34354437467935
H	-4.24206607144249	0.35549843397446	4.09485000987869
H	-5.04549363155028	0.06888549704549	5.65893921461368
H	-2.70384881854768	1.45472232567489	7.34323571305959

H	-3.62631954753339	0.00189045847365	7.81196587990921
H	-1.84720179027281	-0.06363031838854	7.69381417581343
C	-4.62529010953089	-6.00775648104714	3.53786668291164
C	-4.21975515750816	-6.46811783061848	1.12087229721779
C	-6.55340819209945	-6.05431141442771	1.91976293544413
C	6.71353962236735	-2.39860637044398	0.76622523353941
H	8.19094840055133	-1.41255316916274	1.99427373517053
C	4.43655652906031	-2.22156989784357	1.51342454941554
H	4.09538663242838	-1.12674699068653	3.33331467337450
H	-5.22216708666624	-5.48136254174170	4.29557894923136
H	-4.73570901190575	-7.08867084319154	3.71294622885009
H	-3.57020286780121	-5.73950837543693	3.68727963323523
H	-4.34566504943364	-7.54128659326009	1.32708322507514
H	-4.51338839518280	-6.28829856381692	0.07707880267079
H	-3.15201035468542	-6.22592304646472	1.21949224955155
H	-7.20575984483110	-5.52382762008436	2.62790205655486
H	-6.89519737752910	-5.81658716788471	0.90209314036706
H	-6.68044434684720	-7.13539999200002	2.08365672899618
C	5.36121451585773	-2.69197468459883	0.58298431837961
C	7.71936593566535	-2.98452499736212	-0.19227644759447
H	5.02434149809009	-3.30599977560019	-0.25181710272842
F	7.33602836302924	-2.82562023593431	-1.49334423039245
F	7.86518851322473	-4.32543376292973	0.00483692895458
F	8.94968156752599	-2.42706298336368	-0.06425456675315
C	-5.06354644332008	-0.58510487175462	-2.34980062461095
C	-3.90292633535708	0.06863399442568	-2.76385803647165
H	-5.03571497202629	-1.64387478290302	-2.09480239243574
C	-6.25177991298576	0.14035393601376	-2.26056500999532
C	-3.92450594291301	1.41451085865146	-3.11718598914306
C	-6.28764696126094	1.49891110699109	-2.58693318999730
C	-7.52055056889161	-0.56839003067841	-1.85586326394196
C	-5.12159071748769	2.12700993159188	-3.02136539173765
H	-3.01079188708600	1.89292630159143	-3.46561740287120
H	-7.21402525666916	2.06270267868746	-2.49411274892197
F	-8.12391254524940	-1.15567843309788	-2.92991567321254
F	-8.43274722152705	0.28522262304547	-1.31217860102241
F	-7.29221355436009	-1.54974073363401	-0.94649310191113
C	-5.12519536813022	3.57705213543125	-3.43411609799632
F	-4.10384127194021	4.26724119233717	-2.85129904755842
F	-4.95810019488948	3.70551546061823	-4.78281388016301
F	-6.28069683520098	4.21013657929426	-3.11368650077444
H	-2.44998198413518	-3.94314283241953	-2.38499758041804
H	-1.13832649258068	-5.24924333736630	-3.51699097732604
H	-0.33645598630250	-7.40327798774395	-2.34827952974109
H	1.18610801996702	-6.28365653333545	-0.72452997344990

228

Minor1

C	-0.50924055700997	1.00867440599439	-4.42697253156850
C	-0.37890789768404	1.72198090371704	-5.73660837703674
C	-1.76820625579577	2.14708791572996	-6.28108537951683
C	-2.59440012489676	2.99095508023863	-5.31596832055596
O	-3.13525898156144	2.16443212700305	-4.28380031372959
H	0.23860748082654	2.61923972325936	-5.58776934734667
H	-2.35521588735493	1.25499358989990	-6.55044380110987
H	-3.41451018615294	3.47740714793193	-5.87697624568085
C	-0.94390524390766	-0.39283013744475	-4.40035730927497
C	-1.28322015983462	-1.02523612707892	-3.18756839170804
C	-0.96937711870588	-1.16931597980444	-5.57676746590585
C	-1.61676140504934	-2.37314824880939	-3.14627597135913
H	-1.30343084161829	-0.45380475032226	-2.26384672103685
C	-1.30076236655918	-2.52081701146125	-5.53672633023153
H	-0.69905575436948	-0.72240519529536	-6.53225247098717
C	-1.62212409071959	-3.12905332804447	-4.32061773505242
H	-1.87899820070979	-2.83338882248476	-2.19382749713440
H	-1.30095867077153	-3.10527927372302	-6.45771566717576
H	-1.86964397673139	-4.19047778788789	-4.28616128755012
C	-0.16417354200653	1.68632246140752	-3.27381648779454
H	0.15566278700448	2.72708461082139	-3.35780628310561
H	-0.65028379546508	1.43676107497052	-2.32622098902993
H	3.62892977800522	7.81756104531162	1.88950943886549
C	2.97610775196563	6.94928639342997	2.05928914872399
C	3.82501958302167	5.75126400803828	2.50720786034136
H	2.44515210928685	6.73846000367926	1.11914007596679
H	2.23654464780106	7.23455616257111	2.82126713137158
C	2.97826915774069	4.49507411203327	2.73194573855239
C	4.52676310734980	6.10942022935800	3.83450565672512
C	4.89112419552142	5.48730841154956	1.42422557970603
C	3.47379415693121	3.70828566743086	-2.86496218851705
C	3.59810859157074	3.26558080509186	3.01176690385589
C	1.57756248514543	4.50145069340370	2.69123191001724
H	3.78924127196173	6.30946556797016	4.62525104240789
H	5.17585271013922	5.29084642935058	4.17622145695161
H	5.14877159667579	7.00828607772659	3.70561964898294
H	5.51487232086820	6.38383293207465	1.29006505808677
H	5.55544082350870	4.65492808394192	1.69364955211665
H	4.42141901527862	5.24678591577478	0.46097395196878
C	3.34303205196046	3.36398506315820	-1.51133432186812
C	2.58334668770704	4.59705460719187	-3.46861321361233
H	4.25640627700158	3.23713128458995	-3.46186133962787
C	2.86012231060425	2.11063367860434	3.23314311791080
H	4.68714233551618	3.19820491063015	3.04470903511380
C	0.82971745436320	3.34284795568253	2.90483786436058
H	1.04010626352140	5.42354036308439	2.47327555100115
C	4.30645211671794	2.42376674007582	-0.89935309078758
C	2.27639388950282	3.92884805542692	-0.79237705544615

C	1.52833978653333	5.18018425988916	-2.75099216276472
H	2.71798047342333	4.82319513054073	-4.52630956177629
C	1.45537583442472	2.11819041189894	3.18175252268043
H	3.38652237336957	1.17500010297444	3.41653216636116
H	-0.25522557911047	3.40532129993489	2.85748054109289
C	3.88200988687325	1.34742471147162	-0.07704167282938
C	5.66112160431388	2.49891335984250	-1.16800341108342
C	1.39810419610042	4.81996458297501	-1.39925779281952
H	2.13387778014503	3.68246101955990	0.25757520328066
C	0.57469967575405	6.20569729168766	-3.37236557849247
C	0.70340583967875	0.87953210457550	3.47985227192191
O	2.50788320459009	1.25376259348524	0.19983903471109
C	4.71608746962434	0.36937792901951	0.43914543323093
H	6.03761692325559	3.32565058080402	-1.77337470272881
C	6.56920787308187	1.50491079592962	-0.73467468409195
H	0.58003283932887	5.22080954760571	-0.80101931719977
C	0.60667261208895	6.16534510901431	-4.90783822556553
C	1.01363918431462	7.60913428064473	-2.90185749933746
C	-0.87300821704577	5.95318661328216	-2.91353694523870
C	-0.56009629834298	0.56775909277738	2.89924842366577
C	1.20066165355217	-0.03809831746482	4.39499454647707
P	1.70736387532103	0.12745104667074	-0.62151834784323
C	6.09843440728037	0.39920958799181	0.05007286128005
C	4.18353380054133	-0.68365278190573	1.34143634073344
C	7.93833103915190	1.55393886975032	-1.10768638650126
H	0.34876784694523	5.16511176904965	-5.28855923844565
H	-0.12702199022809	6.87831607547666	-5.31051558803787
H	1.59056819041153	6.44272348365118	-5.31191854248826
H	0.98362550364202	7.67972333061669	-1.80528815319747
H	2.03987464113639	7.83046579433320	-3.22933374128167
H	0.34314757270653	8.37860212886164	-3.31449174080279
H	-1.55153040711469	6.66307310207265	-3.40936778152926
H	-1.20303719200239	4.93543890269424	-3.16143325425457
H	-1.00143362749177	6.07318591937101	-1.83078667880182
O	-1.04875453405546	1.40028099287635	1.87411905334238
C	-1.34738715371798	-0.50538522259415	3.28799674779051
H	2.15287432038795	0.16710827346153	4.88660591784282
C	0.52441673425634	-1.23604682907494	4.71661888908302
O	2.44882580282338	-1.25260780519376	-0.23748042715284
N	0.22543685580593	0.19441288095409	-0.18019890775323
N	1.85827384889212	0.20113099786196	-2.23959911276780
C	7.00881369825333	-0.64380285730664	0.36052055696342
C	4.79025937777801	-0.92035417478159	2.62270052280609
C	3.09128792117703	-1.46862802785353	0.98783163197318
H	8.28588224455455	2.40645029866246	-1.69487495334429
C	8.80333861892652	0.53711525456678	-0.76675315281443
P	-1.12293065667354	0.77048707249004	0.38219973353752
C	-2.74449385662008	-0.64807785359727	2.80276888314728
C	-0.77509659411511	-1.48328870249147	4.16616950690018
C	1.10934413918826	-2.20494275274323	5.57478317941412

S	3.02280210481720	-0.36221332353365	-3.27784171642579
H	0.93543442022891	0.93637040872799	-2.86197069165206
C	8.32683181398822	-0.57860700462292	-0.04261152065243
H	6.65418206642601	-1.51377687791233	0.91107011194564
C	5.76842584538170	-0.05286686023067	3.17705771852270
C	4.35833241144016	-2.04093693923898	3.40451534533032
C	2.64903274749285	-2.59192224588261	1.74328593199318
H	9.84979965249220	0.58078180163522	-1.07187799783636
O	-1.97421183335148	-0.60831653641505	0.54248235995813
N	-1.87467903949619	1.77084369200973	-0.58001542637060
C	-3.03011315893842	-0.72314627284349	1.44726206591509
C	-3.82837471967079	-0.73171049137713	3.73860994741613
C	-1.41084842067578	-2.71774198639093	4.45622507271549
H	2.09544365675353	-1.99678370418835	5.99540784773697
C	0.46082776518092	-3.38980043407104	5.84648928009248
O	4.31631345574844	0.25422833850293	-3.03848734538983
O	2.40635382443126	-0.26725183710576	-4.59228675293267
H	9.00307779210087	-1.40163553172026	0.19269083521181
H	6.08446968173131	0.82111361146766	2.60906632172730
C	6.30765577164402	-0.29220129187800	4.42413249463131
C	4.94938803090350	-2.27273513390277	4.67481672538594
C	3.32202926021446	-2.86987512274777	2.91895435217597
C	1.51501165944122	-3.44946099597835	1.32738516206199
S	-2.52729264107136	3.21867395228730	-0.40074714635424
C	-4.31559130272256	-1.06648523530434	0.93649586809037
C	-5.14090452410470	-1.03247513167163	3.24740736211999
C	-3.66458616108282	-0.49065448049374	5.12700656837242
C	-0.80328513563143	-3.65134410427483	5.26928145775777
H	-2.37989057953124	-2.93520675763306	4.00901872500513
H	0.92416281498957	-4.13299929265914	6.49737420963758
H	7.04798252311833	0.39686911578253	4.83308109345692
C	5.90647601594501	-1.41869003674598	5.17787471122663
H	4.61684202087492	-3.13892032615675	5.25100231197252
H	2.99154144386481	-3.71453236961279	3.52583434988335
C	1.63928105295492	-4.84543029878090	1.30840257643110
C	0.25909389014818	-2.90178467582354	1.03677413572485
O	-1.61197819355455	4.19267834886081	0.18403558926426
O	-3.16844020307679	3.54077457539632	-1.68059490015773
C	-5.33380070743675	-1.23319870649027	1.86189402182169
C	-4.59488639448375	-1.29524140247435	-0.50001251443333
C	-6.22583563513467	-1.10020581500638	4.16065985697728
C	-4.74079182923777	-0.55447995711273	5.98747067637404
H	-2.67840889178684	-0.23004217891351	5.50853352287704
H	-1.30062907253768	-4.60274133037158	5.46350353087228
H	6.34746591507082	-1.60245294105063	6.15857618710148
C	0.53682639177873	-5.66000738272820	1.03716385379962
H	2.61279340975046	-5.29957915050232	1.50591539597542
C	-0.83449584345697	-3.71698343341733	0.77747664481764
H	0.12323427222572	-1.82525940265055	1.04921904135286
H	-6.33465867027030	-1.47713991203898	1.50147262379695

C	-4.21709400243970	-0.39593726450261	-1.50434396719885
C	-5.33567872726354	-2.42721375768130	-0.88479168008324
H	-7.22070677308079	-1.32436002013578	3.77081739518464
C	-6.03198700823931	-0.86719554643488	5.50493874623104
H	-4.59581184176186	-0.34653220897212	7.04841118265156
F	0.13013364220837	-5.06791728060766	-2.41615656214753
C	-0.73038681486874	-5.11608052082687	0.78473435247814
H	0.67911136088813	-6.74039307255259	1.03212872938323
H	-1.79731238632064	-3.24135299688614	0.58733497246487
C	-4.58788192795650	-0.59980536943383	-2.83346530594206
H	-3.62537620075128	0.48305712520576	-1.25601733592405
C	-5.68996505859048	-2.63288814188898	-2.21444368820561
H	-5.62362860473745	-3.16118425795316	-0.12930249796985
H	-6.87406870653224	-0.91273199367040	6.19699798621900
C	1.27756439693017	-5.33477023576713	-3.09788973153381
C	-1.98688669741269	-5.97084704522087	0.58759656274628
C	-5.33253164288156	-1.72037045890923	-3.22133000599223
H	-4.26893787017694	0.13832092696149	-3.56937026911612
H	-6.25948828612128	-3.52900514272633	-2.46784450568929
F	0.96212082456187	-5.31539160569118	-4.42362803306103
C	2.36064826412991	-4.33764412067445	-2.77162959895127
F	1.63524903554407	-6.60908202321021	-2.78940687492724
C	-1.65815236233422	-7.46546194754476	0.47248433428584
C	-2.74525330675082	-5.54264759187386	-0.68503121848570
C	-2.90400572352420	-5.75717688140331	1.81222913629925
C	-5.77266894956090	-1.95415344831420	-4.66938784806030
C	3.50426726054357	-4.74780448016761	-2.08704566739145
C	2.19708783791826	-3.00331584985309	-3.15531887136469
H	-0.98519511344875	-7.66460787090301	-0.37448376331852
H	-1.18704377864537	-7.85242900478927	1.38772524707256
H	-2.58345657446179	-8.03685246306438	0.31003622354769
H	-3.04866860705780	-4.48720304028627	-0.65415211725647
H	-2.12864090058362	-5.69662351681093	-1.58087519467153
H	-3.66086412141285	-6.14371705925971	-0.79160489244449
H	-2.39195257965946	-6.05172272898212	2.74001704578536
H	-3.19981113357000	-4.70284249250855	1.90937757250834
H	-3.81869780615099	-6.36173308289697	1.71388798022349
C	-5.13500065154109	-0.94343145306775	-5.63443591906879
C	-7.30727822215837	-1.81382185808688	-4.74565931879409
C	-5.36985550531395	-3.37122779237171	-5.12481336996946
C	4.51114182009217	-3.82198827484121	-1.80811246996756
H	3.61694158948967	-5.78770009799521	-1.78697631165641
C	3.20736279758533	-2.09495232677531	-2.84892030774020
H	1.29832953701789	-2.67586247803484	-3.67977333948959
H	-5.42709947922035	0.09039307848798	-5.40263604494450
H	-4.03739763758106	-1.00031864971696	-5.60613234585438
H	-5.46166905237122	-1.16330077601374	-6.66147003304144
H	-7.65497088928740	-1.98736363076065	-5.77561949444251
H	-7.80866210518546	-2.53940786635781	-4.08936050103071
H	-7.62303792941248	-0.80613069940909	-4.43934061814393

H	-5.68603333369558	-3.53369363017386	-6.16630186350054
H	-4.28015290061038	-3.50049942347504	-5.07159650953438
H	-5.83811362829190	-4.15179722193595	-4.50958540443785
C	4.37263445899391	-2.48875901283948	-2.19170723737738
C	5.73151363183406	-4.25615148329767	-1.03574798492670
H	5.14693386663908	-1.75246697706152	-1.97717320202433
F	6.83993411770088	-3.56422028896770	-1.40013350561906
F	5.99981371568581	-5.57844136773256	-1.20516502163369
F	5.56439572947512	-4.05895122285766	0.30789248084387
C	-5.08086229514306	2.47240747972743	0.38612003802109
C	-3.84776843705855	2.98081656076364	0.79474789777865
H	-5.26971993238783	2.27530094750490	-0.66854481947594
C	-6.05426136853947	2.20910510745117	1.35073490894577
C	-3.59241964558059	3.25838194779281	2.13474599355000
C	-5.80517965106158	2.45256850150067	2.70420358264826
C	-7.40654029187879	1.69848347421965	0.91980343497472
C	-4.57497341324778	2.98384648224989	3.08787973492554
H	-2.62990427364929	3.67840724220078	2.42193221236147
H	-6.55989987650967	2.21932255967239	3.45305408796522
F	-7.32834350987788	0.88930378869621	-0.16674276822972
F	-8.24106847853875	2.72677594444013	0.59016857787397
F	-8.02605228063523	0.99263224462903	1.90771308679166
C	-4.28191054168169	3.29892159520472	4.53290431862423
F	-4.23739064895364	4.64651898871257	4.74510740961904
F	-5.21507438812156	2.79283712814363	5.37674422404717
F	-3.07050587393623	2.80649477624649	4.91855954490634
H	-3.13097541287933	2.66143532237310	-3.43660580325180
H	-1.97112694172800	3.79590039084260	-4.88866937949377
H	-1.60546601511737	2.72013402084170	-7.20777158138468
H	0.12946582475985	1.10059568517408	-6.48749475188154

228
Minor3

C	1.92860181386002	-1.87080513388904	3.32480199712494
C	1.93788644937499	-2.89034810671592	2.22827136585414
C	2.23490763972932	-4.32116141358400	2.70075345423365
C	1.06901271212393	-4.93163262477516	3.47772314451700
O	0.62538058516553	-4.06222138680852	4.52354690271269
H	0.99295537156878	-2.85450858615400	1.67141262280549
H	3.14788259419318	-4.32297582378788	3.31581758174632
H	1.39095779088219	-5.88518761667464	3.93059098344853
C	3.12876493313949	-1.72450418198557	4.16359072907497
C	3.00001608510383	-1.43933973221889	5.53331819349864
C	4.41386215609368	-1.80364003800115	3.60170639581462
C	4.12878041704791	-1.23273444426478	6.32081764848036
H	2.00535670685579	-1.42207041450926	5.97976129098446
C	5.53904912209497	-1.56373558293202	4.38565913810940
H	4.53731945984391	-2.00725874926399	2.53809403193303

C	5.40181438132756	-1.28181315022166	5.74615281131939
H	4.01638791011868	-1.02894405553828	7.38673049885883
H	6.52808894271888	-1.59438476150462	3.92673261247648
H	6.28602065915612	-1.10320080249140	6.36015746582845
C	0.92356692793302	-0.93061145664320	3.41894204155308
H	-0.04553099337915	-1.11183649588539	2.94649988465002
H	0.96324860263186	-0.19694112866585	4.22569534301659
H	4.81497511909101	-3.44762026882352	-3.07876234053206
C	4.63779093581201	-3.93966289762391	-4.04422285084426
C	3.34037518333656	-4.77228005137644	-4.02311439375332
H	5.49627146735491	-4.59476406672595	-4.25547329805385
H	4.61379755111173	-3.16566460578992	-4.82310374172538
C	2.12119944955134	-3.88276754644425	-3.75549424272919
C	3.49132747824156	-5.88372239731676	-2.97493274032381
C	3.14828180478796	-5.43022220528202	-5.40745920461821
C	4.40720358527649	-2.64057248315607	-0.60123026602015
C	1.92367839694215	-2.71354713006513	-4.50884255777301
C	1.12000707375435	-4.21322562945421	-2.83094571998349
H	3.58917433885129	-5.47207494366229	-1.96118536581829
H	2.63844399296575	-6.57721930196494	-2.98590197327885
H	4.39735991028097	-6.46952981498713	-3.18742756513458
H	3.07736201027951	-4.67212033663879	-6.20016816471436
H	3.99974476904289	-6.08888802277862	-5.63802524603665
H	2.22839026990565	-6.03206174449104	-5.43170434837555
C	5.20605186094234	-1.48777368264325	-0.57692303989614
C	4.79102554688144	-3.79283417502172	0.08937562123924
H	3.49150883111152	-2.66105260718722	-1.19170102041668
C	0.77043078714618	-1.95165857235354	-4.38019470843431
H	2.68088612789480	-2.38727317173809	-5.22371608331455
C	-0.04348642500830	-3.45224136862386	-2.69426206120413
H	1.22449510502214	-5.09834629243598	-2.20391879467599
C	4.94249289547765	-0.31254172219150	-1.43638075168318
C	6.36358539975802	-1.52177152613673	0.22284829725514
C	5.97397945828029	-3.85331489524374	0.83481378412990
H	4.14692257681598	-4.66840151963131	0.02253829760261
C	-0.25569881250334	-2.31428334321572	-3.48816406165212
H	0.66989703864407	-1.04397173310218	-4.97417077324881
H	-0.79107581636785	-3.76698190389253	-1.96874677021148
C	3.63693039083516	0.18131435665118	-1.72191874120975
C	6.00122081088505	0.33848204544103	-2.04606410652046
C	6.73807348129620	-2.67495779937012	0.90260391945348
H	6.97020551072949	-0.62029604899386	0.31878164384642
C	6.45618543886924	-5.12897176303551	1.53285060910077
C	-1.49863011997803	-1.50705845690019	-3.48309172562249
O	2.56805498083801	-0.45066253741199	-1.08869072205072
C	3.38032561408303	1.18894418226879	-2.63956437460792
H	7.01483345070832	-0.02975234219425	-1.87923687300709
C	5.82313858535030	1.46459103370026	-2.88110744421106
H	7.65258023313840	-2.65427518972486	1.49875284042846
C	5.48914186230120	-6.30383388028562	1.33015260601411

C	6.60458717691160	-4.87441522394723	3.04556648431489
C	7.82765118260109	-5.52428459663546	0.94614577249394
C	-2.26764580995153	-1.22820765613199	-2.31669836672213
C	-1.95201752875631	-0.94747553904897	-4.66839760347886
P	1.48266681870193	0.29537888988616	-0.15718643683741
C	4.49596516595628	1.90831949981550	-3.19029793940108
C	1.99837166975367	1.53373330212545	-3.06412602197314
C	6.93365217553605	2.16076073605027	-3.42651636023185
H	5.36462706924489	-6.55127192014630	0.26559541277955
H	5.88596000339542	-7.19587038473857	1.83534420064487
H	4.49844621457952	-6.09351713346637	1.75656552187800
H	7.32664872118281	-4.07264014966908	3.25284267994104
H	5.64494773485931	-4.58495834814816	3.49548884635179
H	6.96059478732752	-5.78604565043442	3.54839800056164
H	7.74909584489171	-5.72195052000369	-0.13272416550022
H	8.57403351986209	-4.73106179122955	1.09098720633077
H	8.20111224617962	-6.43502058556518	1.43804399331698
O	-1.88805772717413	-1.85545861350092	-1.12697066561305
C	-3.38962313704886	-0.40404421397161	-2.29929152795826
H	-1.42075963143747	-1.16532067713838	-5.59576470621688
C	-3.06179277525494	-0.07817732806771	-4.72407802956695
O	1.32429695314655	1.78346978474057	-0.77788100486559
N	0.27993372383925	-0.68102439242139	-0.22139610512973
N	1.96414458831927	0.62027655793684	1.37053500790412
C	4.34346650893100	3.06238198138574	-4.00152504970578
C	1.62833954108260	1.49314905773312	-4.44989951596291
C	1.03607235556229	1.92366575489693	-2.14726160901467
H	7.93829316092911	1.80459665648637	-3.18919289845653
C	6.75101300968482	3.26990716344822	-4.22330965946294
P	-1.23153362424904	-0.99209760691623	0.07873251297097
C	-4.11164806584287	-0.13429783951861	-1.02628239464229
C	-3.78273433683900	0.23281832930293	-3.52479167616028
C	-3.44855387292244	0.53017167713439	-5.94861080418221
S	3.25388164029120	1.54112860924786	1.85423426941493
H	1.50691480299715	-0.15972419152084	2.35139980348600
C	5.44376832733778	3.72804356828121	-4.50101112555505
H	3.34333465955913	3.43706994393534	-4.21248726295677
C	2.47109574827481	0.94775013828154	-5.45320710975367
C	0.33007134760851	1.96624210602483	-4.83155324719026
C	-0.21640121907943	2.50201509221147	-2.50163335277565
H	7.61195468813917	3.80298933741908	-4.62898567672901
O	-2.01430224362618	0.41814497093356	-0.02599490258487
N	-1.57699021431445	-1.69773304676730	1.45394411318896
C	-3.40732707446060	0.30947751873430	0.08487734677734
C	-5.52855891646584	-0.31619793596627	-0.89511632600096
C	-4.82010193230216	1.20084476556182	-3.60230771863110
H	-2.89920059172816	0.26367836139422	-6.85390718231072
C	-4.47331680330539	1.44933379097450	-5.99158925059086
O	4.43041633142599	1.25155517085645	1.04997891087526
O	3.28334818994761	1.41770831569606	3.30163345741271

H	5.30164867678106	4.62335366482091	-5.10785318412088
H	3.44833387998416	0.55827521636481	-5.17112088123971
C	2.05522611451931	0.87920709472311	-6.76701793122205
C	-0.05944506014872	1.90058961629447	-6.19556747770718
C	-0.53311694839547	2.50523350453707	-3.85070750495635
C	-1.12597962375185	3.13904819151234	-1.52311626706217
S	-1.93693418231108	-3.22670875150991	1.73145204235281
C	-3.99964200632750	0.64624132543115	1.33365911863971
C	-6.16441669582317	0.08757419511221	0.32621445054242
C	-6.32479631963795	-0.91891385390011	-1.90249021401010
C	-5.15267671900618	1.79671460615318	-4.80128506081095
H	-5.35292882531801	1.48169067644780	-2.69545849690258
H	-4.75617590480230	1.91623248645755	-6.93614437241717
H	2.71272185418016	0.44057303728673	-7.51906256956029
C	0.78234110762020	1.36531574117205	-7.14606811895476
H	-1.04995408252795	2.26941290458851	-6.46850744326614
H	-1.47599215813212	2.95080641921284	-4.17208394082392
C	-0.65506292094858	3.93280948302023	-0.46268415265724
C	-2.51277223849080	3.04086644750821	-1.68720787287327
O	-1.21807520116348	-4.16159871273166	0.86452308359387
O	-1.85951237537944	-3.42687341806515	3.18268582239442
C	-5.38187590873807	0.57393880297580	1.39899227982580
C	-3.19395599014336	0.97386599514799	2.53052837197871
C	-7.57023151823756	-0.06468802124234	0.45749305430925
C	-7.68537047821520	-1.07509504112259	-1.73392739808307
H	-5.84605337224369	-1.27616202528482	-2.81279197845257
H	-5.94763613963496	2.54336039671456	-4.82943501514472
H	0.46933626267743	1.31110778100679	-8.18982973923081
C	-1.54196795293828	4.59558101367471	0.38002174696729
H	0.41578207632023	4.05197659835617	-0.31038428508017
C	-3.39506406594376	3.68815671553542	-0.82454371433322
H	-2.91376168121935	2.42198146337064	-2.48830189084729
H	-5.88707807216243	0.86243099505711	2.32197299348913
C	-2.00804897611485	1.72196418315946	2.48440496232527
C	-3.61181322079640	0.50252191466185	3.78790912956870
H	-8.04208353496853	0.24852492625969	1.39063781784161
C	-8.31879904678391	-0.63115918089749	-0.55137066974468
H	-8.27295393051298	-1.55872519037934	-2.51534038430212
F	1.78782112383808	6.58682949948079	4.13261367038361
C	-2.93519323149484	4.48659813526819	0.22996174774825
H	-1.13655665698356	5.21224170211934	1.18270769173857
H	-4.46444384394888	3.55092956110929	-0.98232963989737
C	-1.27076874211973	1.96898581674825	3.64130863880551
H	-1.66000405436776	2.13748886859763	1.54225196913931
C	-2.87031957231702	0.75554296899829	4.93588986531147
H	-4.51774811536224	-0.10133422842110	3.86165736228052
H	-9.39567159212782	-0.75670153609597	-0.43042351854925
C	0.97058256196817	6.09706536748770	3.16116083481145
C	-3.87737722212908	5.24262413529334	1.17172917069389
C	-1.67160885853646	1.48694842183895	4.89305531402677

H	-0.36099492389725	2.55973365501855	3.54814666158495
H	-3.22883492271483	0.35462754033922	5.88520165325923
F	0.33711525495728	7.16026247452589	2.59692270369880
C	1.74054299734910	5.29189784446838	2.14362562586958
F	0.01945626128413	5.34717492128709	3.78511774882631
C	-3.66608382031212	6.75718126008885	0.96328651708683
C	-3.57038277366105	4.88502684761137	2.63953368778501
C	-5.35121967037618	4.90900916224286	0.90026305166219
C	-0.88251156784885	1.74647659112741	6.17952638018093
C	2.18487363694264	5.90100105100698	0.96827987316621
C	2.04731857589118	3.95630179534369	2.41053493771153
H	-2.63374296670939	7.05492927327804	1.19528116857269
H	-3.87564235423406	7.04393941140779	-0.07759057726052
H	-4.33858458910363	7.32808699852119	1.62141062066561
H	-4.22265336210225	5.46807758297752	3.30710697158776
H	-3.74414897613319	3.81773760377221	2.83050962448840
H	-2.52952385059762	5.10581728629216	2.91059800691236
H	-5.55031412305586	3.83440975788386	1.02777811519206
H	-5.98698792207336	5.45519689149540	1.61176881825288
H	-5.66091131871543	5.20125067871725	-0.11387762477245
C	-0.64029360930640	0.41713417372994	6.92365349797449
C	-1.70790979748805	2.68934555627245	7.08032259425544
C	0.47927285391264	2.40379337353045	5.90832624489018
C	2.95230368504139	5.16855305936618	0.06144023816196
H	1.93912414907735	6.94138885310139	0.76287952494719
C	2.80527612563243	3.24058712320926	1.48637928284730
H	1.72456696987163	3.47753244707014	3.33467635653183
H	-1.58007877923050	-0.05325428677300	7.24217763378764
H	-0.10926462479705	-0.30256684509012	6.28521143770894
H	-0.03364593800407	0.59474875901902	7.82410109696988
H	-1.17469413412841	2.88131423809301	8.02396687453231
H	-1.88138870455982	3.65296347966048	6.57965027352985
H	-2.68706824309094	2.25343659320201	7.32324012955603
H	1.11573465131893	1.79241505077253	5.25136152516779
H	0.36529579387525	3.39950819947025	5.45675450891259
H	1.01874487834278	2.53592155062883	6.85719031725590
C	3.27427622678899	3.83376387206377	0.31600051075808
C	3.40303550209644	5.79846314739333	-1.23391214019666
H	3.88685003434653	3.25895518722400	-0.37854171934204
F	3.35230453059520	7.15524331814493	-1.18977553763512
F	2.60783953589093	5.40412812984542	-2.27230851274145
F	4.67423351097177	5.44635571239092	-1.54935140569300
C	-4.03868177207895	-3.87464326173710	0.06440942598445
C	-3.67721671122474	-3.37767680445063	1.31404474420589
H	-3.26450991489162	-4.17901107497376	-0.63840089728802
C	-5.39263194218134	-3.98405581945468	-0.25886217721476
C	-4.64347849802860	-3.00328385716334	2.24557675793837
C	-6.37599852970468	-3.61407413111118	0.65884897635885
C	-5.76434741211962	-4.55436391146963	-1.60387084181329
C	-5.99335237995412	-3.13009152859754	1.91119293593410

H	-4.33526834575744	-2.63505619723282	3.22411227999490
H	-7.42907101585662	-3.69749107568181	0.40012371545087
F	-5.08168261987449	-3.94615506230426	-2.61601658563747
F	-7.08885937887181	-4.42935787056410	-1.87754745404853
F	-5.46466484969262	-5.88279486230213	-1.67898769197184
C	-7.02990171754808	-2.78136747837702	2.94811993263711
F	-7.01014319613274	-3.66322352455476	3.98690396646824
F	-6.81139124664699	-1.54517082146775	3.49169089847651
F	-8.29188760870990	-2.77948789453021	2.44938557513915
H	-0.24965219422075	-3.72412821055992	4.23636220075169
H	0.24099974530875	-5.14788427495275	2.78506998858129
H	2.43591818123475	-4.95189839327938	1.82023395445710
H	2.73227065709848	-2.59259034309221	1.51928390191601

228

Minor2

C	-0.93034602940178	-4.20497057391540	0.22015079754393
C	-0.77301158331704	-4.04850940179910	1.70052888914358
C	-0.56489151320281	-5.40545663782164	2.41030627099062
C	0.89814343509980	-5.84855453015295	2.42855145837967
O	1.47238482511033	-5.91076060993098	1.12137398155973
H	0.05951155938540	-3.37061232915688	1.92610298776327
H	-1.18098496002313	-6.17853883779776	1.92662257359195
H	0.96644591691191	-6.85747455655204	2.86801817794187
C	-2.09046079825549	-4.97577657732767	-0.26481111574265
C	-1.99926643615342	-5.76035186263490	-1.42711840351649
C	-3.32085153450103	-4.92941271889474	0.41875012038641
C	-3.10460968563085	-6.45958912547114	-1.90281103619590
H	-1.04605473554308	-5.83311166780550	-1.94877311268725
C	-4.43209515204668	-5.61243027467210	-0.07015101414576
H	-3.41525795105052	-4.32651724927120	1.32229469079741
C	-4.32796621910712	-6.37904030836630	-1.23372667193701
H	-3.01234394660327	-7.06498332330010	-2.80557952081393
H	-5.38600637526775	-5.53930703725420	0.45337040406971
H	-5.19865039716185	-6.91489248693241	-1.61457641806119
C	-0.07769774502397	-3.58486236243214	-0.66121748555190
H	0.84968897228911	-3.13361831064808	-0.30470358843219
H	-0.15060663639405	-3.79530895388007	-1.72904097514289
H	-4.53732412737546	2.29750644098328	5.25712526128614
C	-3.73171529676417	2.38968030156323	4.51290059549793
C	-2.41163606705478	2.78387230771735	5.20645822716282
H	-4.03728957140244	3.14444871005668	3.77609899082156
H	-3.63382992826093	1.42686194231227	3.99380063738293
C	-1.30427320858887	2.92771152300858	4.15677057117073
C	-2.08502891617100	1.72182967365949	6.26534782713374
C	-2.59985149041845	4.14111893104547	5.91593700298998

C	-1.94635397730084	-0.83996546379222	3.31922601460632
C	-0.11224762623181	2.19210756699064	4.19408762381492
C	-1.45547075141593	3.84323922916517	3.10256413562253
H	-1.16615254641954	1.96440469470482	6.81936526978988
H	-2.90724543633246	1.66887402957123	6.99361105410064
H	-1.97111631737400	0.72411549643832	5.81913004227079
H	-3.40241875405459	4.06974936029664	6.66589771832659
H	-1.67554230973463	4.44668044432566	6.42729298233163
H	-2.86911572637658	4.93316799064522	5.20324179339107
C	-3.18985237514590	-1.24688491922652	2.79860830162023
C	-1.31333525536583	-1.56491464302254	4.32068421135100
H	-1.47254168597611	0.07334530176165	2.97065635388843
C	0.89021960570609	2.36884260191853	3.23955747861579
H	0.05545326255921	1.46414561447139	4.98744277589825
C	-0.46025795562730	4.02676714614525	2.14991307866421
H	-2.37257909142745	4.42616282583858	3.01013479449548
C	-3.96480930167317	-0.38883922742568	1.87050159910649
C	-3.73380752913740	-2.43887710325335	3.30862908939913
C	-1.87831446593142	-2.72364444469940	4.87299716255284
H	-0.34920634849452	-1.20055791497456	4.67674239911573
C	0.74318595610799	3.30319295124149	2.20414950611861
H	1.80072659267616	1.77782003665771	3.31804258757602
H	-0.62820514391163	4.72821766414320	1.33185447655332
C	-3.34972284524267	0.54163026191130	0.97930880830166
C	-5.34936964267765	-0.35584069471824	1.93697189356904
C	-3.09592234810782	-3.15468284410825	4.32432422388977
H	-4.67976373640909	-2.81513539397868	2.91344678093195
C	-1.20214612015652	-3.40605060633708	6.06576423436303
C	1.81669209236416	3.59790969697402	1.22873045611136
O	-1.96266072297690	0.47044717036941	0.84398360424221
C	-4.04063202856412	1.56459020399497	0.33741775257703
H	-5.86515058540601	-1.01906003795756	2.63225247832702
C	-6.12663537110053	0.53029969181689	1.15777395844723
H	-3.56775422822988	-4.06685907459450	4.68903505897648
C	0.28974359577945	-3.66270521487525	5.77511567001418
C	-1.86796000886158	-4.74017481237378	6.42877916745356
C	-1.31797275245582	-2.45190518890741	7.27591957425361
C	2.62639583124907	2.58851338910219	0.63695680848608
C	2.07933520548918	4.90624034681860	0.85466652099695
P	-1.25570461838989	0.20939086274847	-0.58654931952355
C	-5.47322027136790	1.52320486915406	0.35657945992914
C	-3.30581433705408	2.70219991667171	-0.27943223052507
C	-7.54549839731912	0.47994126657861	1.17571685197021
H	0.77565626808957	-4.08342767467077	6.66787837456894
H	0.41857540278319	-4.37650833644595	4.95103472830003
H	0.82502485928276	-2.74447414138791	5.50064242962024
H	-2.91554855290196	-4.60625151959974	6.73564157617891
H	-1.84269764850392	-5.44962485785442	5.58818477229950
H	-1.33344100203028	-5.20229548610168	7.27095474996656
H	-0.81165720463576	-1.49698777266902	7.07717271573756

H	-2.37112332519255	-2.23484517971337	7.50644696663832
H	-0.85438883878253	-2.90678274023133	8.16432937115522
O	2.36867986370410	1.26175852388897	0.99601593591146
C	3.66376070498283	2.84482425951456	-0.25095833354525
H	1.49981687333208	5.71260886989436	1.30822342442450
C	3.07527997705009	5.23754222332652	-0.09241865949508
O	-2.07645299352732	1.14898945757002	-1.59999649731158
N	0.20945557368865	0.65583268322459	-0.37757651159219
N	-1.60387252017023	-1.29596313411772	-1.15235585658507
C	-6.27221238888580	2.40421736482264	-0.41604423481664
C	-3.53786988082201	4.05437622781347	0.14738790476036
C	-2.30878390649874	2.47884055247988	-1.21352105211235
H	-8.03194074963154	-0.28044681934338	1.78966124139636
C	-8.29399069618222	1.35486712613455	0.41810811267007
P	1.70444823465258	0.25613451426277	-0.09313263806040
C	4.47586805249106	1.72583194088119	-0.79776022699786
C	3.88343831555845	4.19982761511517	-0.66529856144440
C	3.27491326139417	6.58168383618223	-0.50665640285188
S	-2.27090969876081	-1.61226850289642	-2.65044012019149
H	-0.91490476835692	-2.36736780086938	-0.75615959839744
C	-7.64903415464485	2.31959120122819	-0.38888332906304
H	-5.78402021610617	3.14024240975726	-1.05331567167876
C	-4.45294898280380	4.40111478986325	1.17544560909429
C	-2.76650623036293	5.10513382976451	-0.45458142067052
C	-1.50924519699980	3.49255694527776	-1.80958692179352
H	-9.38335135223008	1.29864175723115	0.43232600959681
O	2.44551355835926	0.67264074278808	-1.46877978377079
N	2.06657067525917	-1.23343594311827	0.30901014487026
C	3.84328930941880	0.66063076455741	-1.42209897292433
C	5.90354873719041	1.69210614263682	-0.68237685224686
C	4.82908504368648	4.55864802368757	-1.66185585462193
H	2.65919667970196	7.36148966250859	-0.05324755561070
C	4.20940531252788	6.89660513587617	-1.46850931778706
O	-2.20262998080313	-3.05665152766617	-2.79926255164108
O	-1.72606997890227	-0.75683292016754	-3.69053131434813
H	-8.24291521988560	2.99337404641489	-1.00752210537842
H	-5.02124685549320	3.61501804789759	1.66810299581477
C	-4.62424766993539	5.71335281045467	1.56632511960038
C	-2.97889127788403	6.44721354195680	-0.03973478451639
C	-1.78157549952407	4.79389561461443	-1.42051652356911
C	-0.42027677286131	3.19533312697271	-2.76504906030465
S	2.56645897985652	-1.80136596759395	1.71520974012201
C	4.52075828927271	-0.43844371949991	-2.01870829138122
C	6.61838103633332	0.62365365584572	-1.31994334919413
C	6.63747787204722	2.63949246030567	0.07645799255708
C	4.98567841030503	5.87153422832565	-2.05595884392005
H	5.43145717511422	3.77891285123874	-2.12509046273640
H	4.34698583087161	7.93184703268714	-1.78367120792287
H	-5.32916015349685	5.94971990660789	2.36489249218586
C	-3.88948542458284	6.75029879850202	0.94831551353210

H	-2.39134686998263	7.23547440110381	-0.51513841503920
H	-1.21199115663439	5.60952051630207	-1.87014023844702
C	-0.55506979856697	2.25492216475598	-3.79452946639258
C	0.80041742668304	3.88481914698022	-2.66475274337355
O	1.83844684375014	-1.25424268277314	2.85983796504799
O	2.63557278769609	-3.26001385859729	1.57788861426231
C	5.90585219557637	-0.39912183097772	-1.98740594881540
C	3.80057419679176	-1.60170717505072	-2.58351660180366
C	8.03463951567634	0.58591678526282	-1.22279565684362
C	8.01168300809023	2.55992611285612	0.17132327617598
H	6.10037909615158	3.42536455206887	0.60549349790304
H	5.71292562337087	6.11895248940420	-2.83076279532432
H	-4.03781305055334	7.78459618095241	1.26154059089433
C	0.48811071752488	2.01711851572592	-4.68896770053265
H	-1.47814291527406	1.69111922718260	-3.90683926336286
C	1.83223006341338	3.64797391153506	-3.56507135207681
H	0.95529522406362	4.58997977091172	-1.84665271372842
H	6.47231419834161	-1.20072091684529	-2.46340676605271
C	4.32630071737053	-2.88931829950767	-2.37746067674497
C	2.59864182173793	-1.50073206382408	-3.30242598065570
H	8.56825934153759	-0.22947797711202	-1.71472932363073
C	8.72004209538262	1.53379094318852	-0.49413449310704
H	8.55341792491117	3.28837752061416	0.77608670581313
F	-7.78348886008372	1.49870694847843	-3.60436243115243
C	1.70312130931117	2.70828771507060	-4.60138661907318
H	0.33420025142328	1.27055605286824	-5.46724654679152
H	2.76361788868469	4.20202781443106	-3.44119444621770
C	3.67830164648701	-4.01862421895066	-2.86182072305280
H	5.24421746340443	-3.00548734758619	-1.79927148001424
C	1.95250464094562	-2.64073059135686	-3.78328837162273
H	2.14833624320828	-0.52625013249989	-3.48644142653539
H	9.80691728247020	1.48343122605559	-0.41472670394587
C	-6.48303147595492	1.24711547020049	-3.89997949591659
C	2.85589742747971	2.49300185268871	-5.58764469765271
C	2.47041231038130	-3.92629197233385	-3.57246813491904
H	4.11904709967379	-4.99656095289844	-2.66254841529315
H	1.01409467095837	-2.50872984341432	-4.32214032626804
F	-5.79099891703333	2.40702431601599	-3.71869982806600
C	-5.90883388525710	0.11885735601587	-3.07932555749875
F	-6.42261579482442	0.96310444044729	-5.23196130961527
C	4.13489527676295	2.10727006695763	-4.81889335251741
C	3.10300555081301	3.81112966358332	-6.35076641885753
C	2.55386709756420	1.38614953882279	-6.60742685142662
C	1.79528624454026	-5.19526183919883	-4.10083597378651
C	-6.73281333425585	-0.69529315556069	-2.30327331345043
C	-4.53633215053510	-0.12797151832488	-3.15824796907268
H	4.97154883920069	1.96922003195767	-5.52026217239930
H	4.42196466650592	2.88481242321978	-4.09865657860419
H	3.99617245838235	1.16915718007473	-4.26533619179681
H	3.93600287294154	3.69088867344794	-7.06029080817363

H	2.20825551801154	4.11007332297863	-6.91586372236145
H	3.35693509118990	4.62978773508602	-5.66286850057347
H	2.37790209373360	0.41722450994716	-6.11761304910694
H	1.67623940522414	1.62690303246046	-7.22484440084376
H	3.41360181422927	1.26684348267785	-7.28235791076327
C	1.68133575872883	-6.24146220615064	-2.97250391290618
C	2.66687125799878	-5.77519054384358	-5.23492903137443
C	0.39188422167005	-4.91640498598428	-4.65956930956019
C	-6.17580774076685	-1.76755182950850	-1.60188488326431
H	-7.79955610859091	-0.49275750752984	-2.23852079677442
C	-4.00413487675488	-1.20247067885185	-2.45339139516418
H	-3.88311393359266	0.49241933247918	-3.77059001780969
H	1.12900275951646	-7.12245050166373	-3.33274177617428
H	2.66600373368905	-6.58790777606602	-2.63150466164016
H	1.15964842495244	-5.83784978516727	-2.09369256023994
H	2.21723485239887	-6.69968914780278	-5.62897941006797
H	2.76005396716622	-5.05705995543862	-6.06249885209024
H	3.67868213233898	-6.01328017656241	-4.87749573975550
H	-0.27543054415199	-4.45863307989971	-3.91574643727105
H	0.42570282872222	-4.24860457265030	-5.53242802243289
H	-0.06680684651508	-5.86130436494630	-4.98601403172419
C	-4.80757997854931	-2.03383279793704	-1.67317055012662
C	-7.05651845230879	-2.70029844588548	-0.80772941874286
H	-4.37407459008188	-2.88719986375966	-1.14939566948290
F	-7.16233567782180	-3.91770972470387	-1.41340038300817
F	-8.31686415363396	-2.22288769394859	-0.65321176722236
F	-6.55668810279324	-2.93247335738327	0.44131341251820
C	5.29376440416667	-2.01896476189521	1.31636150064404
C	4.27407010198950	-1.26455416366915	1.89333353512767
H	5.04660808751257	-2.92678792677531	0.76628964653093
C	6.61876203018121	-1.60880089352055	1.48104161572309
C	4.56114725299414	-0.12027715195495	2.63264251333970
C	6.92383442148361	-0.45006830689152	2.19751052716546
C	7.71847826551584	-2.47460339107058	0.92148794756588
C	5.88854932334954	0.28863520868113	2.77063388815781
H	3.75039966587606	0.43486415961212	3.10042654280558
H	7.95640935177036	-0.12527416876273	2.30421038116960
F	8.93509170049628	-1.87568848790305	0.97268076973687
F	7.49013083785754	-2.81140093836381	-0.38354393303851
F	7.82067169569754	-3.64851302409269	1.60687697477292
C	6.17550514055313	1.51815061294829	3.59368670155322
F	5.92861161626869	1.29648294357700	4.91762926193405
F	7.46581737856602	1.92747572464021	3.49291443898339
F	5.38560789707064	2.56732808362419	3.22482095554588
H	1.91791533611051	-5.05154798214448	0.98374754640188
H	1.48590644711688	-5.16443164407909	3.06381384309931
H	-0.92222009882495	-5.32334830538052	3.44879882642822
H	-1.68257594132602	-3.57670977769711	2.10292264116263

228

Minor4

C	0.86547433110944	1.64618439006809	-4.07147179717820
C	0.79101287203164	1.57125983328296	-5.56654488527000
C	2.18856280216397	1.54212105484972	-6.24723611515141
C	3.21226715013029	0.66026718105486	-5.54493894156296
O	3.64234413261930	1.32392345772650	-4.35746204741304
H	0.26099075095098	0.64293928675467	-5.82278968218517
H	2.60855082051266	2.55881350879352	-6.29323066540608
H	4.07356551235942	0.49704832968612	-6.21975790668206
C	0.96683718207222	2.93941915348914	-3.38948013208499
C	1.27401036607996	4.11334810780642	-4.10367575343851
C	0.73535678303615	3.04702367521969	-2.00117242305861
C	1.38508983061773	5.33919039955172	-3.45341603405518
H	1.45224890189439	4.06306540265060	-5.17688692444020
C	0.81580106308029	4.27740610112770	-1.35849140556552
H	0.47303253226184	2.16615995537313	-1.41621235976515
C	1.15582831864139	5.42584272635558	-2.07887350903978
H	1.64584016972606	6.23215211000513	-4.02268278641745
H	0.60257331933426	4.33850460113547	-0.29034911372015
H	1.22391342375366	6.38934844476851	-1.57215401554221
C	0.76288367739523	0.47022361535146	-3.35116344188544
H	0.75019204980595	-0.47814002054626	-3.89277303401977
H	1.19435175051554	0.42454863920313	-2.35332981466964
H	-4.42329807460327	-8.41069405861554	0.50182139056910
C	-3.93418300508970	-7.56571623923408	1.01011023689743
C	-3.14253143388587	-6.71587341144086	-0.00535888071522
H	-3.26852058912873	-7.96776150468124	1.78757779392665
H	-4.71320209011926	-6.97042751151548	1.50679922316467
C	-2.49660225485123	-5.54250396467039	0.73805839171816
C	-4.10583619260602	-6.18597074439244	-1.08740733041392
C	-2.10177863457874	-7.61072991665449	-0.69238565977415
C	-2.72140284181131	-2.67826530183844	-4.18598730084454
C	-3.29838157555718	-4.54982011533040	1.32503791224165
C	-1.10976596939124	-5.40414826339874	0.88505862316026
H	-3.56930973935417	-5.57580992881940	-1.82693179082981
H	-4.57679209908462	-7.03067335896952	-1.61232765538262
H	-4.90808045524864	-5.56973058280327	-0.65894574242830
H	-1.50345384216212	-7.04700925002237	-1.42341380221039
H	-1.41757969812335	-8.07833315508611	0.03063252333153
H	-2.61387243136746	-8.41980985523411	-1.23296139122846
C	-2.70314091451514	-2.89590569346878	-2.79836632688891
C	-1.66968026416874	-3.11037702881057	-4.98787006628821
H	-3.54941277859854	-2.12061272258036	-4.62637063170536
C	-2.74477160312856	-3.46938968002111	2.00055452582225
H	-4.38541142342556	-4.60848124215650	1.24366635165412
C	-0.54662396016105	-4.31809766909358	1.55547245276775
H	-0.43952224177383	-6.15171318655424	0.46195429952873
C	-3.82795714395052	-2.40277899728005	-1.97472326534449
C	-1.59028057222045	-3.54967443460264	-2.25056780286127
C	-0.55970578657625	-3.78464037377415	-4.45112740271698

H	-1.71496969344927	-2.90412305511267	-6.05840220035403
C	-1.35395846574158	-3.31733916421501	2.11785038929186
H	-3.40507929009814	-2.71176816126352	2.41953390464334
H	0.53591256558796	-4.26353597484378	1.65605328570774
C	-3.60513174689404	-1.64419230630581	-0.79652819298415
C	-5.14371125675917	-2.56972710431574	-2.36484913290263
C	-0.54571301516518	-3.98691100209795	-3.06394721872139
H	-1.53924153426186	-3.73101753174003	-1.17834696047593
C	0.59136886124067	-4.22746148740441	-5.36105613614264
C	-0.78630665215462	-2.17687309904567	2.87005060308622
O	-2.26757346300577	-1.46630715388848	-0.40324682331914
C	-4.59953988095919	-1.04979886211609	-0.03525655375042
H	-5.36288464239799	-3.16685347308671	-3.25203676521381
C	-6.21206044850125	-1.93757058929344	-1.68686257944845
H	0.30350649176253	-4.48086963048581	-2.59348992423262
C	1.38458279214427	-2.97511563722871	-5.79232921695338
C	0.03599899931762	-4.93609903815894	-6.61204931812703
C	1.55267440262990	-5.19123946175401	-4.64937729820530
C	0.45542359859881	-1.57112673388270	2.52222997412182
C	-1.41711227415543	-1.67949846284522	4.00036391719571
P	-1.62291076995159	-0.02211850678169	-0.67930453139681
C	-5.94795830247222	-1.13415650161395	-0.52669417118058
C	-4.27129656105538	-0.33302538911618	1.22497335838831
C	-7.54447150313359	-2.04357071454856	-2.16586842369875
H	2.18157063034963	-3.25312792989708	-6.49922470558931
H	0.73013168220675	-2.24081840725214	-6.28584599346685
H	1.85638984594209	-2.50507358263925	-4.91844400256239
H	-0.59273707936038	-4.27237686584664	-7.22096835739729
H	0.86780576787803	-5.27745474596256	-7.24611029121060
H	-0.56584197464311	-5.81354663788166	-6.33382178687179
H	2.32466838246364	-5.52553547612212	-5.35788652833935
H	2.06739692839225	-4.70999272761355	-3.80726314796874
H	1.02351232409981	-6.08388001338992	-4.28340222992948
O	1.04892470719659	-1.93368447842657	1.30650031137288
C	1.12836326396660	-0.66808163650498	3.32645465522761
H	-2.36405034468794	-2.11942001764383	4.31792195502519
C	-0.87004477168553	-0.62696347644346	4.77073314505470
O	-2.58945019945722	0.99697235663585	0.11359474854280
N	-0.16420832295739	-0.02517363809172	-0.14815811762647
N	-1.69655038869966	0.52612588167099	-2.21166575790380
C	-7.02456806333290	-0.41226603538665	0.05067811345026
C	-4.95960240200667	-0.64729504347019	2.44891036022583
C	-3.29175327123302	0.65261460878888	1.27449729178440
H	-7.73215178372389	-2.66881206354555	-3.04115710605987
C	-8.57263512932104	-1.35182488547433	-1.56363516244815
P	1.18318024730268	-0.79667955561033	0.15186263214741
C	2.54897367189838	-0.31923431689057	3.05947212805045
C	0.43291487035072	-0.11957379563901	4.45321330547417
C	-1.57794554736564	-0.07186644584944	5.86916697678372
S	-2.91377454396070	1.23005919890219	-3.08982588866808

H	-0.53526205054913	0.54706078743835	-2.86781141294437
C	-8.30307178874079	-0.51460543901345	-0.45815260589337
H	-6.83251086086308	0.24325385488919	0.89824317803619
C	-5.82813450790296	-1.76410121193109	2.57382885779807
C	-4.72692577643590	0.15995957146848	3.61048396214900
C	-3.03943539404122	1.46196135178232	2.41682700570604
H	-9.58947643054439	-1.43180411183912	-1.95051442844972
O	2.04101512675699	0.40743196046628	0.83077299301568
N	1.80827336918373	-1.57169524431834	-1.06946563321301
C	2.96550023252729	0.25053063905383	1.86373728023618
C	3.53591171120791	-0.61062111966820	4.06020907978230
C	0.95484388912222	0.94692630855512	5.22857640508213
H	-2.56422751817777	-0.47613269538030	6.10649779025334
C	-1.04346692113217	0.96426452549361	6.60369762295808
O	-4.07289810407564	0.36141579701351	-3.18374131334235
O	-2.24990221388222	1.70886370476663	-4.29348537649377
H	-9.10960213908276	0.06459411816536	-0.00644384981179
H	-5.99052436452088	-2.40631680457016	1.70964296099266
C	-6.45266701649587	-2.05202610609885	3.76968722033089
C	-5.40287220056630	-0.14917034451561	4.82047568107130
C	-3.79581141036038	1.22124296680287	3.54762570663123
C	-2.01400504601715	2.53116081216326	2.42478899068371
S	3.26275627703107	-1.70488194179892	-1.69938644706118
C	4.28075100984364	0.77232008933090	1.65800546935154
C	4.88026698315833	-0.16366126458386	3.85069351691613
C	3.25198813918370	-1.37284470645548	5.22300629684835
C	0.22915456853681	1.48329008067542	6.27161115603511
H	1.93252741725065	1.35527780179109	4.97433018148854
H	-1.60190990818518	1.39064639403646	7.43853400387962
H	-7.10474725021746	-2.92365110603676	3.84119990707123
C	-6.25116452461946	-1.23157969699795	4.90275024546966
H	-5.22468616112682	0.48597807880683	5.69093015458421
H	-3.61952024255098	1.82541960752889	4.43910149403118
C	-2.34294066668128	3.83937350638011	2.81394671081814
C	-0.67689139615950	2.25177371543934	2.12904002100429
O	3.21385258357267	-2.74944008201619	-2.71582965031441
O	3.82895175070465	-0.40870913740112	-2.09641758847964
C	5.19324566011846	0.56201735571761	2.68125505315170
C	4.69595492109715	1.51885555700069	0.44900089802561
C	5.87332839046423	-0.46172491245960	4.82089218423166
C	4.24318445479267	-1.66383209166592	6.13764122173095
H	2.24300831168188	-1.75186209363276	5.37819061371670
H	0.63943552073996	2.31721878787492	6.84302114263849
H	-6.75799173341369	-1.46404511746303	5.84031502271249
C	-1.35867417192717	4.81999977877694	2.91073791886894
H	-3.38343517111137	4.08711241077419	3.03475940707024
C	0.30480989917492	3.23413568924133	2.24069394514820
H	-0.38872196654458	1.24607706990421	1.83592840454246
H	6.20105585066655	0.96620747862178	2.58144678687959
C	3.83858546602910	2.36355830578582	-0.27132615182852

C	6.03028201247142	1.43802492463083	0.00935616503494
H	6.89270020765914	-0.11189130263897	4.64544701622972
C	5.56378002093241	-1.19883547341761	5.94304444286707
H	4.00654105776433	-2.26852840603130	7.01422973716962
F	-3.37614190233149	7.35002518636199	-1.59502924083323
C	-0.00837726983025	4.54017178711721	2.63767098820767
H	-1.65192342750366	5.82673730303550	3.21391044653920
H	1.33617465066840	2.95604712757071	2.02652608242454
C	4.29120999465521	3.08003688050109	-1.37865339093319
H	2.79882572406076	2.47079764312273	0.02983963957381
C	6.47428696788207	2.15160066064422	-1.09576785711883
H	6.73349683279329	0.79279618525934	0.53463876678878
H	6.33658428805553	-1.43462401995299	6.67606098313618
C	-2.50284548114969	6.30702797492802	-1.65992565780973
C	1.05850682027562	5.62624974516811	2.81339913550611
C	5.61552213344982	2.99057917699472	-1.82423850217720
H	3.57971891944958	3.71286037026892	-1.90608502234622
H	7.51707864443759	2.04269514383241	-1.39936616887888
F	-1.61557807655184	6.48218782917844	-0.63586151254224
C	-3.21301027463056	4.98017936269882	-1.55831999121999
F	-1.81052560717565	6.43102757360749	-2.81863861662476
C	1.10709110631556	6.02884771421369	4.30220468967469
C	0.69392670282470	6.86016089935890	1.96402982570680
C	2.45432103527481	5.14336003172966	2.39316860771797
C	6.14900331304523	3.77489032513274	-3.02554842620402
C	-4.33708915646752	4.88463585117852	-0.73498848637165
C	-2.75161994092516	3.86954889748903	-2.26674870536566
H	1.87167936575781	6.80380521623418	4.46410256333646
H	0.14076906007761	6.42745761780074	4.64151271600450
H	1.35450622039402	5.16130759689380	4.93125929624656
H	0.65055925850320	6.60377069718912	0.89632723497800
H	-0.28250160644452	7.27603288542598	2.24673842410103
H	1.44958330143126	7.64856996170967	2.09822160177138
H	2.48650067698374	4.84272692789594	1.33583670728092
H	3.18085386430480	5.95733099596940	2.52818528840598
H	2.79503464072918	4.29138585306517	2.99936514371191
C	6.80816177115095	2.80618225942031	-4.02920295051066
C	7.19786392137148	4.78976526248173	-2.52466603034677
C	5.03619026207655	4.53581724969541	-3.75842181547293
C	-5.02765382876917	3.67728531720653	-0.64843696254263
H	-4.69023747240254	5.75743373001390	-0.18847619807712
C	-3.44993724081062	2.66691285013253	-2.15366606205629
H	-1.87857191506417	3.93917700739538	-2.91407167578223
H	7.64642877826630	2.25734801654789	-3.57798693672377
H	6.07051653933342	2.07907494045815	-4.39601275971424
H	7.20248709701648	3.37139307746993	-4.88762340906816
H	8.03282448707318	4.28952416716935	-2.01390067757029
H	7.60931862460946	5.35949529168076	-3.37195025211722
H	6.74653364589347	5.50132480351204	-1.81767217121658
H	4.26892508147545	3.84403318569614	-4.13172418824563

H 4.55225477489330 5.28023188153664 -3.10936121790249
H 5.46611287229232 5.07210713651265 -4.61748166529509
C -4.59649207342811 2.56138010390334 -1.36884396085462
C -6.24472607201892 3.55764411195076 0.23479915499808
H -5.14384092471258 1.61946769890718 -1.32967505218257
F -7.27441327001204 2.94371722354580 -0.40453521433584
F -6.69337524244049 4.76684772779484 0.66175727847499
F -5.97720199252330 2.81797572494285 1.35252380927976
C 3.81681399564453 -3.17051456867089 0.58381336914658
C 4.32874520022797 -2.30419197002282 -0.37740228958385
H 2.77474511189338 -3.48192544540095 0.53735781128531
C 4.64243745550230 -3.59950297600538 1.62606151382035
C 5.66233469182031 -1.89502029449182 -0.34168176045924
C 5.97294785106637 -3.19151192202615 1.68809567338165
C 4.07775113342239 -4.53140402865640 2.66820954526664
C 6.48040730109375 -2.35330973967508 0.69095416928551
H 6.03682368749365 -1.20994452689286 -1.10042714678925
H 6.60897849698499 -3.52071263559298 2.50878633856410
F 4.83859613368666 -4.57452990234130 3.79010812725082
F 3.98544575868180 -5.80893237840110 2.19552894846916
F 2.81915542517259 -4.16547758001294 3.04272029872237
C 7.92049419013076 -1.91926967874998 0.78292563332140
F 8.10786827277056 -1.00709691134418 1.78937192812051
F 8.74579515670520 -2.96520605361213 1.05764350899599
F 8.36320809516313 -1.33926428244912 -0.36015390989648
H 3.73867899660548 0.66848957692728 -3.63004521474842
H 2.78755991222208 -0.33176158917555 -5.31483471768831
H 2.05137106136342 1.20299813513958 -7.28581373194457
H 0.20059000402438 2.40421706958593 -5.97336457277871

228
Major0 0 CyH
C 0.94473141428316 -4.19981807836452 -0.23309679206668
C -0.22832611741739 -4.26243603611512 0.69081128640455
C -0.93883652614500 -5.62184431542995 0.73168275958922
C -1.70777913437315 -5.88334445626153 -0.55801015000613
O -0.86161880311072 -5.70816588251891 -1.69617404048352
H 0.15451959045097 -4.02696068419854 1.69426847293756
H -1.63962525939611 -5.62768063644424 1.57511651717559

H	-2.56985033135430	-5.20685260300768	-0.62131262625955
C	2.00077710361023	-5.21290789880284	-0.13497349174325
C	2.67996247388174	-5.61265479740019	-1.29433575742025
C	2.35163686898838	-5.80466711971737	1.08739615566105
C	3.68520882127887	-6.56695933490926	-1.23540853660169
H	2.38308571388029	-5.19356301590802	-2.24855709939175
C	3.37079396720829	-6.74378848918099	1.14728463607460
H	1.85914900592775	-5.48955499255120	1.99894773313214
C	4.03920220607186	-7.13032505874318	-0.01245867836361
H	4.18842861068999	-6.87819779442971	-2.14552660247833
H	3.64807131763876	-7.17480682655078	2.10374096689060
H	4.82968138724641	-7.87230349531818	0.03724330954781
C	1.11348581917116	-3.11082966958497	-1.07281556289307
H	1.94508675267619	-3.11352054556095	-1.77255495925619
H	0.21695590249900	-2.58168930980064	-1.38970398434178
H	2.83306590232814	5.25598936229937	-5.12035799279830
C	2.62792107296798	4.47057890863017	-5.85418499454459
C	2.53100496190256	3.08624245904893	-5.18224994396839
H	3.43702273752318	4.47477604986849	-6.59309844834535
H	1.69180252320727	4.71996183953351	-6.36382722986957
C	1.37119233883054	3.11651857780355	-4.18410452157945
C	3.85526360308219	2.78720218591777	-4.45705872114775
C	2.33329108351096	2.02909931580866	-6.27497998403446
C	2.41973397836720	-0.71499361630103	-3.41176601691540
C	1.41402019767534	3.99150020378980	-3.09096956279277
C	0.21928041739179	2.33936069392050	-4.33044077121331
H	4.05932263622349	3.51896884738449	-3.67068531429816
H	3.83739355890344	1.79346461637490	-4.00051874140690
H	4.68677345028296	2.81767028625007	-5.16980754236350
H	1.43716540220699	2.22641243483299	-6.87191599434677
H	3.19246394355405	2.04258480132734	-6.95305913224547
H	2.25944551175765	1.02121090086001	-5.85305221428203
C	3.61818663074934	-0.95301001940210	-2.71958426996538
C	1.87481309656929	-1.67856221354440	-4.24484445347267
H	1.90212883376461	0.23018597038921	-3.30748419404854
C	0.35063243669491	4.10560430099949	-2.21002060474829
H	2.29222197489326	4.60568882086168	-2.91865429240395
C	-0.85311713636446	2.44930319495559	-3.45094127773984
H	0.13328260032130	1.63498867713612	-5.14976243577560
C	4.26074328127540	0.06888316084723	-1.86643841509103
C	4.23622985233661	-2.19388348009271	-2.91238825559487
C	2.49534604898155	-2.91708018141373	-4.45178497930431
H	0.92981371742328	-1.45285135670446	-4.72862393333120
C	-0.81564507968977	3.34922608913812	-2.38276547307314
H	0.43310771301319	4.77674811788659	-1.36042040125040
H	-1.72758791694832	1.83249254560834	-3.61465974906684
C	3.51907419813508	1.00851890505800	-1.10342534450156
C	5.63622598857395	0.18256440233254	-1.81338302042667
C	3.69305624294963	-3.14661921841524	-3.76899419718821
H	5.14547092275436	-2.43375212170591	-2.36949825327060

C	1.83811002393443	-3.95025444743284	-5.36544304302445
C	-1.95925936654254	3.55949530439503	-1.47127470193878
O	2.13136806368605	0.91395982790256	-1.14316162868261
C	4.08519982229637	2.04189737773859	-0.37808515171841
H	6.24779823849459	-0.47922603783636	-2.41734191206644
C	6.28348822067201	1.13419700388415	-0.99972324732515
H	4.21412953220193	-4.09071380870265	-3.87921825791349
C	1.64314831302401	-3.33823425678905	-6.76594215376384
C	0.46639540575529	-4.33638472301392	-4.77739038411372
C	2.68327770704712	-5.22077635744212	-5.51335962570430
C	-2.72488738344493	2.48843829380800	-0.94754071088171
C	-2.31984481055882	4.82997794698681	-1.06937681591978
P	1.34619647920958	0.44915582505006	0.18836247511752
C	5.50756232465210	2.07956933361669	-0.25935173788411
C	3.21697381007869	3.08046765691620	0.22967384795067
C	7.69544934861615	1.16767023092419	-0.89342074616146
H	2.60449326956154	-3.03859805918036	-7.19601356928510
H	1.17999993353003	-4.07313216596832	-7.43299996394020
H	0.99469043931275	-2.45855572479056	-6.73666933889261
H	-0.18600664166314	-3.46610788225238	-4.66101702008628
H	-0.03604240292936	-5.05467734777250	-5.43415551706703
H	0.58151967098649	-4.80443111265305	-3.79432868247690
H	2.81566377655584	-5.73733111321493	-4.55664693322839
H	2.17706901399400	-5.91599347976701	-6.18972273825971
H	3.67140543549708	-5.00403275273444	-5.93292771482550
O	-2.39170041085279	1.19848231184393	-1.34867251261203
C	-3.77666359117181	2.63611213866935	-0.06350851463998
H	-1.78076943240813	5.68331590989322	-1.47052497010582
C	-3.36683821801707	5.06009915574867	-0.15178918831571
O	2.01953425537904	1.35527153762876	1.34095926736563
N	-0.13532075744765	0.81318192675486	-0.08949274221705
N	1.77670823231602	-1.07898323011916	0.59650212708552
C	6.17933389093814	2.99037619181603	0.58930674406552
C	3.35854674479205	4.46455372179446	-0.10183736323884
C	2.18316926584744	2.71673906656376	1.06846728771326
H	8.27645041642654	0.44951422971686	-1.46445712105835
C	8.31803662420227	2.07104377521621	-0.06766316284719
P	-1.59027317958586	0.25410686045581	-0.30358798789920
C	-4.46191135753738	1.43587266774945	0.47774937103118
C	-4.10671479925118	3.95533702644133	0.37735351840524
C	-3.67652149700935	6.37180376494156	0.28521410291808
S	2.34769703731246	-1.51603600252403	2.09486039006859
H	1.47241075163449	-2.14833472024017	-0.18707300104945
C	7.55073966635088	2.98297380378563	0.68533799701396
H	5.59804068245309	3.69007146750133	1.17816310428453
C	4.30755063980114	4.94215976152998	-1.03778930501454
C	2.45923028112883	5.40572788582902	0.49210312762709
C	1.27314626860053	3.62111652365977	1.66824603353287
H	9.40008920047036	2.07912641616870	0.01407780239574
O	-2.33113211148365	0.58182685573916	1.09663534537635

N	-1.82890388989425	-1.26528544146862	-0.72566047381693
C	-3.71660024502043	0.44886344354602	1.09843811654612
C	-5.87525164216764	1.25772291910945	0.38940165313379
C	-5.09307509115806	4.21816554494823	1.35879073264331
H	-3.11518896446313	7.20215979288498	-0.13369892524455
C	-4.65122442679064	6.59241030397706	1.22584254597339
O	2.41659889609070	-2.98406427058181	2.06026830293492
O	1.62519783812377	-0.85125010548768	3.18669391990829
H	8.04550126628870	3.67966168008488	1.35396140139283
H	4.97686257779785	4.23984850482008	-1.51924317024898
C	4.38457217813802	6.27884036079242	-1.34936895872485
C	2.57685806415023	6.77792425582298	0.15981898406713
C	1.45159053139543	4.95740472400652	1.37211893981354
C	0.18159626782998	3.18138305410281	2.56010919735780
S	-2.30612246035358	-1.74994278175090	-2.19447757840347
C	-4.27170063852415	-0.67639493203237	1.76054782563602
C	-6.46686444172250	0.13872715893533	1.05452739466091
C	-6.71013755337373	2.11783747834053	-0.36184237708401
C	-5.35765332130351	5.50215838709850	1.77247808238580
H	-5.64258980472463	3.39066048076891	1.79043180714127
H	-4.87407303048088	7.60156915573309	1.55622914505625
H	5.11649045564798	6.61908843403191	-2.07476883744558
C	3.51889272351471	7.20927753507423	-0.74017838549707
H	1.89432186435182	7.48287443510097	0.62575123729413
H	0.79396105656842	5.69013959688524	1.83060682757090
C	0.37418826539457	2.23390773480906	3.56760722754535
C	-1.08762424799608	3.76116056275666	2.44302883285372
O	-1.67557338780633	-1.00740633414158	-3.30326541277950
O	-2.20513433612289	-3.22598295440587	-2.21075104968629
C	-5.64960200597771	-0.77617137473306	1.74901766441930
C	-3.45061688691402	-1.67694839803078	2.47510051938149
C	-7.87004436711188	-0.03997511978039	0.98591226446812
C	-8.06657404582849	1.90279877884870	-0.42634203744742
H	-6.26610819706907	2.94560315321610	-0.90251689514206
H	-6.11535674622508	5.67643032056974	2.52966120973405
H	3.59384929577135	8.26218164168845	-0.99100494062822
C	-0.65490787619640	1.90139722819621	4.44186161264055
H	1.33585350791902	1.75147266128042	3.68634932313222
C	-2.10726800998290	3.42758654948678	3.32029099534358
H	-1.28310114474483	4.46568920570161	1.63992779248986
H	-6.12840827547601	-1.58627065064350	2.28886220761035
C	-2.28600013349482	-1.33903280846378	3.18161399729306
C	-3.87143886230293	-3.01023255425249	2.53129560502830
H	-8.31072336920451	-0.88870110473134	1.50053559502095
C	-8.65538154198750	0.82240947234316	0.26141932926401
H	-8.68691457488739	2.56503444865994	-1.02112637264055
F	5.26768314143604	2.74022827536658	3.74981311631571
C	-1.91482764193415	2.49764460797150	4.35031639190820
H	-0.44996380560856	1.16505360968350	5.21023279940526
H	-3.07297751973132	3.90503239158476	3.18888499842281

C	-1.61189491297524	-2.28592731193400	3.93756483862852
H	-1.90943519309301	-0.32404834928544	3.16531690947812
C	-3.19987739162011	-3.94916656180005	3.30755069960133
H	-4.74038218743494	-3.32355961394868	1.96036680318892
H	-9.72785252766031	0.66654301384042	0.20574873551882
C	6.05362123013692	1.66319599090942	3.99000323959923
C	-3.04480538244551	2.21165197097413	5.34149088441183
C	-2.06507987025750	-3.60672561975382	4.04725844262357
H	-0.71510509180662	-1.97025686710049	4.46133187536210
H	-3.58592932314001	-4.96170767050027	3.33396391044171
F	5.85569006417244	1.32049905201724	5.28670418185287
C	5.71635634823263	0.52074188687972	3.07158856046969
F	7.33737631537074	2.06035389384111	3.87295361497317
C	-2.68366901307520	1.10257970608961	6.33725076852817
C	-4.31668124644437	1.78423716108985	4.58823146082782
C	-3.33429789902534	3.50351967192314	6.13107201842137
C	-1.34202360102780	-4.58683070171866	4.97360566028675
C	6.70998740671080	-0.16609205869205	2.38480183082719
C	4.38185580808743	0.14018562255783	2.96628022758502
H	-3.53003172501768	0.93069538136379	7.00971370408486
H	-2.46678577024355	0.15706204431823	5.82826834226570
H	-1.82180013223770	1.37489107653438	6.95506247530247
H	-4.14595676628776	0.86232611822524	4.02522290699754
H	-5.13063516787429	1.60416655090847	5.29896033529321
H	-4.64789944774300	2.55455392967858	3.88658333184816
H	-3.64737551279891	4.31447981169860	5.46638886581337
H	-4.13529205139788	3.33045830029386	6.85856195506977
H	-2.44180069361146	3.83528515135101	6.67143752229946
C	0.09436641546857	-4.81950136630438	4.47216662934895
C	-2.05060357335187	-5.94430171810755	5.05192742828316
C	-1.29460616672505	-3.98765972032521	6.39316292255856
C	6.35563494155831	-1.24097072184188	1.57473057399753
H	7.74523072921368	0.13708773810251	2.47057598477667
C	4.05734238022303	-0.93314451469321	2.15185808100614
H	3.60525478611979	0.65718827768458	3.51724967530567
H	0.64138631480994	-3.88199017130482	4.33409273448247
H	0.64850061092117	-5.43797035027482	5.18721210972649
H	0.07554511884330	-5.34838502455537	3.51373054156044
H	-1.50587968190409	-6.59994270546521	5.73838331605890
H	-3.07494757448954	-5.84475703317039	5.42568109209267
H	-2.08207150467926	-6.44254483326261	4.07720958393861
H	-0.81933441510820	-4.69426437699092	7.08221470059748
H	-0.72212010341941	-3.05670790453089	6.42032994687932
H	-2.30486804393200	-3.77571036750620	6.75771547640737
C	5.02694877217850	-1.63428705089627	1.44656791639154
C	7.41263903970410	-2.05116848806823	0.87509640012380
H	4.75158360397412	-2.48537496007311	0.83156104487651
F	8.62754634696396	-1.46764942805201	0.92280435571505
F	7.11083206158451	-2.25271250145575	-0.43253861130791
F	7.53471273836861	-3.27906294326307	1.43476931391086

C -4.96097635436490 -2.26146407316092 -1.62431263168233
C -4.08174910667920 -1.40459014656679 -2.27287842952869
H -4.58168770989626 -3.11476881489245 -1.07212656142680
C -6.32742808655201 -2.01603218887304 -1.71863662505146
C -4.53779227927152 -0.31595286363262 -2.99968380178713
C -6.81137090702779 -0.92516686673755 -2.43395740951753
C -7.27344241735315 -2.99475790679036 -1.08169176949432
C -5.90783638553298 -0.07813206366378 -3.06501572934600
H -3.83152416591025 0.32697489536738 -3.51148188158534
H -7.87481464984246 -0.73225775823864 -2.48771804049774
F -6.92451541239199 -3.27158635414219 0.20188415076399
F -7.26972492624672 -4.17853272866563 -1.74243631484709
F -8.54748651267646 -2.55228615205224 -1.06444416516462
C -6.38288410109187 1.09751045134415 -3.87213580763189
F -7.69469510094696 1.35664664207976 -3.68818156025356
F -5.69640560989905 2.22325826353189 -3.55788257701097
F -6.19820446916003 0.88974344354379 -5.19958104687099
H -1.11438211874385 -4.86288011170205 -2.09756379076284
H -2.08262327140596 -6.91511689437711 -0.56696813086279
H -0.21313156578607 -6.42537294489174 0.89822113355453
H -0.93599069058512 -3.46652329933060 0.43549053217309

246
Major0 1 CyH
C -1.04032901060432 -3.72454181973521 1.49251992993373
C -0.03822948680493 -4.15973502182131 0.47290007261522
C 0.51895471977307 -5.57398072198482 0.68736692030620
C 1.46699779757978 -5.62224309520005 1.87888858060192
O 0.83846370325561 -5.08958031055276 3.04609402221825
H -0.54691455120755 -4.11839639291374 -0.50069613247506
H 1.06054702369090 -5.87975654993030 -0.21591638707990
H 2.38055033442204 -5.05837557519654 1.65056653154161
C -2.21031404073254 -4.56711946454926 1.76193060956049
C -2.77908386035434 -4.57453010058336 3.04335862399347
C -2.78521848530249 -5.37413072414541 0.76858086468350
C -3.89480133093069 -5.35131565589241 3.32078295998770
H -2.31560561089881 -3.99046663588144 3.82960982131198
C -3.91058101797389 -6.13651049220240 1.04245381942673
H -2.37828784824432 -5.36359047318071 -0.23480458028606
C -4.46942774831857 -6.12881686146584 2.31880998204168
H -4.31272818140651 -5.35727164539925 4.32261939756424
H -4.35676986067371 -6.73607331921238 0.25563535167298
H -5.34600679369786 -6.73203527792233 2.53228219821887
C -0.94866772035362 -2.46798244467702 2.06805398166839
H -1.65588300122124 -2.19560947492472 2.84707760143792
H 0.04061403152092 -2.02037422887386 2.13272873589282

H	-1.20706528236818	6.77638846068827	4.10296687027788
C	-0.97717764833598	6.16404502013940	4.98017563914690
C	-1.11120804982853	4.66063487003724	4.66624621435674
H	-1.66760637541775	6.44523451470978	5.78327289412404
H	0.04207003054535	6.40212079801038	5.30084929297302
C	-0.12391924936237	4.31224673123318	3.54981058033414
C	-2.55263178472028	4.36696408368141	4.21462048042561
C	-0.84150253646628	3.87103569180867	5.95278349963167
C	-1.63933369058699	0.56961741861013	3.90418393842780
C	-0.25958866231208	4.90651704734497	2.28853479903765
C	0.95915065631570	3.44869690013195	3.73207029275044
H	-2.81612822989814	4.92424037834691	3.31130068584753
H	-2.68770814092774	3.30154922286096	4.00793868157787
H	-3.25791378733495	4.65168038030250	5.00322664900680
H	0.15342943797434	4.08370289430304	6.35652325094478
H	-1.57539441968348	4.15534317237795	6.71336539665053
H	-0.93164839218225	2.79157423521701	5.79132391450120
C	-2.95182767008124	0.33975368843034	3.46005976845632
C	-1.05862630241380	-0.24537850908689	4.86208471051210
H	-1.05836614446756	1.39038082222892	3.50337702138792
C	0.65463764756878	4.67249017793856	1.27407868810984
H	-1.09413072567375	5.57043854632727	2.08580917828268
C	1.88300576761477	3.21119979315300	2.71896733909236
H	1.10866099829470	2.94898074788769	4.68212982289157
C	-3.62445044329985	1.21182641799369	2.47352392950148
C	-3.64506060864289	-0.73298612729621	4.03318186214422
C	-1.75097956695377	-1.31391126357008	5.44622673640767
H	-0.02925866146935	-0.04498486231632	5.14128827962161
C	1.75840366418223	3.83348821654289	1.47429827466609
H	0.49986593084034	5.12996797846940	0.30164799932584
H	2.71065606347794	2.54105929876661	2.91416556317388
C	-2.93004076487864	1.84928929243108	1.41205388987502
C	-4.97582023326961	1.48073843376208	2.57240651908552
C	-3.06227403385028	-1.53005740842462	5.01370790779235
H	-4.64941187779703	-0.96756521606853	3.69374831374527
C	-1.04897453020885	-2.19617473928480	6.47691751562313
C	2.75361461058786	3.67449423453767	0.39366356882124
O	-1.56670366389316	1.59598631992063	1.29168773639129
C	-3.50910014620172	2.74450226050498	0.53058199343241
H	-5.54182568633959	1.06073538447231	3.39683779577039
C	-5.65577921618312	2.28439335855211	1.63499415634590
H	-3.64656080251474	-2.34615666120784	5.42321344274632
C	-0.53721306591552	-1.31860608527891	7.63517677313441
C	0.14265685136214	-2.89994983231253	5.79873983296830
C	-1.98036214699087	-3.26490135429969	7.06067256891127
C	3.32100183055270	2.42104809444178	0.05487050822135
C	3.15838678745561	4.75822700566688	-0.35976045999858
P	-1.04785959089705	0.73664180677477	0.02804298780563
C	-4.92391723338777	2.92557815862352	0.58783020483067
C	-2.65812656202640	3.49705971600257	-0.42389027479309

C	-7.05931247907910	2.46282142804754	1.70297586752783
H	-1.36429864254907	-0.78184839132323	8.11120621674195
H	-0.05124565393702	-1.94504946375948	8.39073879491418
H	0.19449569593772	-0.58209791761524	7.29274834521612
H	0.83835676627445	-2.18479553924133	5.34969673104077
H	0.69362511556673	-3.49934415597642	6.53159412850930
H	-0.20370277898318	-3.57434392708623	5.00884598738695
H	-2.33969280087980	-3.95712293564716	6.29157351867171
H	-1.43350504259459	-3.85820319688693	7.79971031309729
H	-2.84613491711752	-2.81954104711715	7.56248024187395
O	2.94365967510020	1.31401382613333	0.80846637151472
C	4.22020202846660	2.22068030006009	-0.97471727081059
H	2.77273270200669	5.74386770615624	-0.11595782761180
C	4.05720098566894	4.63042640922125	-1.43991131846690
O	-1.81000804714310	1.42501401481395	-1.21541667428414
N	0.48584928172686	0.96119948941198	0.00921417948945
N	-1.67426615563945	-0.77809353720910	0.07137277632840
C	-5.63581081360076	3.68322852745849	-0.37183444490751
C	-2.62136700054282	4.92660191615500	-0.43157871946600
C	-1.80533183807714	2.82195545807470	-1.27290722281423
H	-7.60644437270749	1.97679940198059	2.50542526898435
C	-7.71986823149722	3.21420745950193	0.76211808791397
P	1.89195830430248	0.26966505429607	0.15334272892222
C	4.69069149113097	0.84791114085285	-1.28831340111940
C	4.59510172944857	3.34652805225077	-1.77157778553568
C	4.41166591229804	5.75254763027156	-2.22905444050680
S	-2.51716110029400	-1.46220041912417	-1.18605245321297
H	-1.34554216456137	-1.67646952660382	1.03423494099195
C	-7.00096490758789	3.82087545371744	-0.28794536317781
H	-5.09192062610189	4.14454117520294	-1.18778572844790
C	-3.37057514362380	5.72302389376200	0.46778966863720
C	-1.74107140965153	5.58351312288638	-1.34841415882098
C	-0.92164463374831	3.44004370907879	-2.19077361712165
H	-8.79682715399300	3.33455175395283	0.81915498730807
O	2.41669960445941	0.15858815609659	-1.37333887619382
N	2.04575816036719	-1.12948341924277	0.90265136058757
C	3.76414410258826	-0.15124920948970	-1.53163015969842
C	6.07630781288582	0.51211301604230	-1.35334502130717
C	5.42970877666767	3.24338494836965	-2.91085276709241
H	4.00544858968317	6.72328883485067	-1.95931877259800
C	5.23716061463482	5.61978196204098	-3.31745900204888
O	-2.71492262348181	-2.86510728840758	-0.79181043741119
O	-1.92399535738125	-1.16098939300458	-2.49509839953295
H	-7.53020275607023	4.39317015943198	-1.04257427695382
H	-4.02129971135298	5.23981757021756	1.18607168743963
C	-3.27695722265739	7.09428778814967	0.44646600613967
C	-1.68063071063076	6.99898284308808	-1.35319004253652
C	-0.92698317467359	4.82012862430661	-2.21210889365950
C	-0.03395297202732	2.66725599945954	-3.08258611716623
S	2.71337706696808	-1.32661212491483	2.36426625773570

C	4.09521998730593	-1.46196879136172	-1.96222359287947
C	6.44334673214431	-0.79673868502442	-1.79601409639213
C	7.09994783040965	1.40555452361093	-0.95903317526664
C	5.74190558671095	4.35019641308565	-3.66386590004779
H	5.82284610106750	2.27410099221572	-3.19157622615007
H	5.49591983488381	6.48662487748847	-3.91647658961711
H	-3.85656371788229	7.68269697701861	1.15038204656371
C	-2.43165648218146	7.74173023360384	-0.47684303382701
H	-1.01565898858672	7.48566927637249	-2.06091487699794
H	-0.28355833164857	5.33768454383698	-2.91769152358777
C	-0.47771388135410	1.54391368562299	-3.78383004068290
C	1.28618004983525	3.08606649460044	-3.28907845127897
O	2.34599884002402	-0.27603696013320	3.33391321686725
O	2.48091024098888	-2.73473633974527	2.75381348631314
C	5.44072104049267	-1.73294662299240	-2.12097909115556
C	3.08020830422276	-2.48969463500865	-2.28083193077405
C	7.81589025246992	-1.13625393053736	-1.87834266366204
C	8.42229842672755	1.03595898321738	-1.02858739929291
H	6.82973862125220	2.38587341425558	-0.58405565190503
H	6.38056846721129	4.24389726762498	-4.53486921569326
H	-2.37071304708696	8.82492822871371	-0.48663777184875
C	0.35807486070574	0.88178361669566	-4.67600075591416
H	-1.48765642050984	1.17972258714322	-3.64670790163146
C	2.11195572376875	2.42375684227308	-4.18376718916785
H	1.67696490015543	3.92807748560573	-2.72547469025562
H	5.74595376455339	-2.70072026988277	-2.50444141630744
C	1.84529866926396	-2.18459380885741	-2.87457951511643
C	3.37065731710801	-3.84135844953321	-2.06341331596716
H	8.08454049676275	-2.13074830062384	-2.22199522148512
C	8.78668852286316	-0.24051030763132	-1.50308296626268
H	9.19106657936291	1.73027573160693	-0.70582025281344
F	-5.30815291967034	2.56465760431397	-3.49825226708533
C	1.66683359134148	1.31095061152797	-4.90913816473442
H	-0.03820428399254	0.01978816061181	-5.19982055049362
H	3.12557403492840	2.78907461936118	-4.31275884145987
C	0.97839383208015	-3.19187300283497	-3.27101939154859
H	1.56149398779863	-1.15540211383914	-3.05494034791498
C	2.50362101026308	-4.84563765481023	-2.48160303994376
H	4.29280429809705	-4.11770726271245	-1.56091876067659
H	9.83437121808655	-0.51797407562880	-1.55749970485043
C	-6.21514532053388	1.57660598831236	-3.31058893374585
C	2.58560486549809	0.65288655241838	-5.94085614665238
C	1.29564822576890	-4.54755726117530	-3.11733773303659
H	0.03560767246190	-2.90023313919747	-3.72380729730878
H	2.79315333697641	-5.87632995648098	-2.31146260090644
F	-6.28657109583068	0.88747893493925	-4.47636932950267
C	-5.81871717277852	0.67250550831125	-2.17634333870516
F	-7.41739065310344	2.15630191583731	-3.11532886764541
C	1.96510521537456	-0.60690931029370	-6.55642219163063
C	3.92368699705557	0.25909064169744	-5.29097941192471

C	2.85224479357411	1.67076654783168	-7.06749228889787
C	0.35533351044396	-5.61860105768878	-3.67481275292438
C	-6.74586371520519	0.27628422253384	-1.21975103402561
C	-4.51557402445676	0.18493949278243	-2.15326921540590
H	2.66907879452919	-1.04549282765161	-7.27058215266392
H	1.74950761301746	-1.36383157993840	-5.79444877465922
H	1.04135473908089	-0.38243402520376	-7.09952019813259
H	3.77096295699403	-0.46256704512165	-4.48372244795996
H	4.58135907690611	-0.19820079410490	-6.03825520815939
H	4.44261528494387	1.12771602172323	-4.87628189218211
H	3.34470765085339	2.56832079514538	-6.68104171423140
H	3.50026097340699	1.22811233059044	-7.83233747891574
H	1.91470065886288	1.97660137021361	-7.54289452246582
C	-1.00460018477535	-5.55745679543665	-2.95734020188177
C	0.92119953047619	-7.03421504709745	-3.51141591333025
C	0.14500657303953	-5.35703679153610	-5.17977467870076
C	-6.35346832463772	-0.60873945387350	-0.22030048673314
H	-7.75905597611305	0.65514544993220	-1.24917419322875
C	-4.15361564255857	-0.69866271106346	-1.14887539955404
H	-3.79705998528728	0.46281224124163	-2.91529165119569
H	-1.44206241317327	-4.55462807574729	-2.98134439127883
H	-1.71163785362807	-6.24698037312319	-3.43258652668618
H	-0.89386542650361	-5.85999860675324	-1.91103014009851
H	0.21914771887561	-7.75728268667557	-3.93825212377606
H	1.87806390824612	-7.15003971375468	-4.03077152515093
H	1.06247911169444	-7.29675932025375	-2.45767935517244
H	-0.49445200466059	-6.13573578455782	-5.60964059170384
H	-0.33495955298872	-4.39117653705344	-5.35937943871498
H	1.10292487119568	-5.36353635438839	-5.70953762425151
C	-5.05377306832371	-1.10359946345023	-0.17190226795750
C	-7.34208331820011	-1.10595843429677	0.79871176047428
H	-4.75355431469357	-1.81341779194173	0.59296063118183
F	-8.52986590849845	-0.47347710301345	0.71849978927728
F	-6.88168370229809	-0.94421412587573	2.06556843650560
F	-7.57772925399262	-2.43100097522657	0.64404727241853
C	5.18615724109777	-2.28616108789610	1.58056201463030
C	4.50190173771698	-1.20015156305272	2.10972947485760
H	4.64935416088377	-3.19034146698795	1.31342077509683
C	6.56659661792740	-2.19724143953290	1.42650844825030
C	5.16290492575684	-0.03775931788216	2.47513469622257
C	7.25281967703982	-1.03830514032975	1.77569977958488
C	7.30822227970504	-3.40661180389609	0.92973304041174
C	6.54060724612623	0.03755484347191	2.29251114665788
H	4.60658969221756	0.78745329903900	2.90340788235669
H	8.32390498830665	-0.97208390884414	1.63689859535178
F	6.76202644607198	-3.90109593568500	-0.21223384219200
F	7.26723092145081	-4.41247539216886	1.83748387964113
F	8.60759907547233	-3.15144264076357	0.67401119715429
C	7.23943671043623	1.30134781658875	2.70933387166183
F	8.53270195699221	1.33008131715610	2.32386290968673

F	6.63465646415346	2.39731468380907	2.18855316284482
F	7.21931525052874	1.45266924788596	4.05679222173236
H	1.23049263206873	-4.21626825908574	3.19623949195031
H	1.75106208600676	-6.66206991636415	2.08766964092704
H	-0.30030086159616	-6.28507781309174	0.84020765428650
H	0.77803343843814	-3.43116344884482	0.42523395901513
C	-4.35924406818328	-4.08161921962303	-3.90439607240981
C	-5.55345643934106	-3.49640486124671	-4.66261327985038
C	-4.81213862418399	-5.07390475555359	-2.82985414534585
C	-5.82871559475293	-4.44122950802444	-1.87634503451614
C	-7.01967472043117	-3.85625277077249	-2.63949187255012
C	-6.55535614438659	-2.85541842081153	-3.69967403706855
H	-3.66756860259650	-4.56915536190521	-4.60263545157841
H	-3.79831656579321	-3.26544617254070	-3.42852067416928
H	-5.21156847925092	-2.76096390151076	-5.40035019647979
H	-6.05686658422128	-4.29792732000342	-5.22220832136518
H	-5.26537793385949	-5.94986226137661	-3.31675786969400
H	-3.94340627923487	-5.43424578688230	-2.26540498739265
H	-6.17296556047022	-5.17701446934687	-1.13968525012258
H	-5.32746439453512	-3.64485018171315	-1.31034767852454
H	-7.57059730557480	-4.67143675776689	-3.13047930117386
H	-7.71859061577121	-3.37832781425060	-1.94312995029505
H	-7.41331672912600	-2.45302434356030	-4.25155210337238
H	-6.07407864760123	-2.00320655795274	-3.19992826163878

264
Major0 2 CyH

C	-0.34146229559983	-4.22783447059007	-1.48982839415645
C	0.56808959018825	-3.61614529644781	-2.50525561907089
C	1.36507335183127	-4.63144935974193	-3.33654879832404
C	2.44825952984165	-5.29872747885812	-2.50005413263704
O	1.88377587696123	-5.88051889282008	-1.32338442685504
H	-0.06525720468913	-3.01826347055966	-3.17583513814286
H	1.83369984814060	-4.10617416615978	-4.17647681956962
H	3.21273762480385	-4.55954800458707	-2.22745197457732
C	-1.32708786839346	-5.22607550662353	-1.91522942088661
C	-1.70850172085755	-6.24358306472269	-1.02915611524182
C	-1.90221136675928	-5.20039665842593	-3.19481735439265
C	-2.64133727435752	-7.19974816254374	-1.40314845711684
H	-1.23706309972983	-6.29740681184897	-0.05525961585104
C	-2.84852299261708	-6.14609217133787	-3.56056035687288
H	-1.64412423415093	-4.40705475164886	-3.88555947263120
C	-3.22013756963319	-7.14973193017461	-2.66840771430105
H	-2.91148964553312	-7.99043098471733	-0.71020967731521
H	-3.30238005855165	-6.09860932150857	-4.54501523024082
H	-3.95463623980612	-7.89306964098195	-2.96136986444909
C	-0.35700633765391	-3.74490502520706	-0.19120374491528
H	-0.98349625347331	-4.23715459062883	0.54814565718239

H	0.55757571913093	-3.28419301801203	0.17730956172686
H	-1.58548616910306	1.12506337583460	7.81210728454488
C	-1.17091528763560	0.12743068420404	7.98587605817273
C	-1.16719786548098	-0.70868577951451	6.69047188250014
H	-1.77847620593093	-0.36609802298898	8.75278413941222
H	-0.15383132082833	0.24633719276855	8.37281058353035
C	-0.29063616819340	0.00869566200933	5.66128487024876
C	-2.61106400000661	-0.83484654921478	6.17209636051131
C	-0.65186198796865	-2.11450660594577	7.02043162503935
C	-1.26085392351978	-3.03001947859903	3.25055410271310
C	-0.66261184319810	1.27478889535165	5.19145042175422
C	0.91686545040763	-0.51273353376491	5.19058941003588
H	-3.04907701983868	0.14299442153406	5.95214029319341
H	-2.65024103054813	-1.43716574231174	5.26026667843832
H	-3.23824163156912	-1.32035823596731	6.92780208300941
H	0.34877517424824	-2.08620275669726	7.46341259141285
H	-1.32187015423629	-2.58458796625739	7.74713540066920
H	-0.62433261422073	-2.75472445053049	6.13221874583020
C	-2.56832175367620	-3.07992046087164	2.74116224674589
C	-0.49405107404348	-4.17872163632413	3.35661089140256
H	-0.83141226412539	-2.08990535262459	3.57292701410613
C	0.14193244178150	1.99216661304440	4.32133628680010
H	-1.59886881596129	1.72160559430190	5.50940121367405
C	1.73141962261743	0.20283653503111	4.31825803595192
H	1.25284175449127	-1.49352931820735	5.50704739880961
C	-3.43883850736841	-1.88714480805154	2.66820270104778
C	-3.06083775828979	-4.33110389456925	2.35285766290180
C	-0.98639087374479	-5.43610646075209	2.98385869327878
H	0.52097333912098	-4.07841935504688	3.72738813304946
C	1.36791253698516	1.47870999214108	3.87979261679592
H	-0.19320365417082	2.96077065788347	3.96349120815379
H	2.66195625702203	-0.24288495373776	3.99144187879335
C	-2.93768862297776	-0.59019926816854	2.38441414833227
C	-4.79309412832869	-1.98264585434727	2.91979623521694
C	-2.29102858395314	-5.48326282828232	2.48446122687411
H	-4.05423153246442	-4.40659832238749	1.92064319367510
C	-0.08515542358910	-6.66444990570461	3.10105993730417
C	2.23426912365912	2.31035076632368	3.01892250641576
O	-1.57468154358837	-0.47558903751715	2.12716964346806
C	-3.70355834087005	0.56237154286575	2.39617558803086
H	-5.21638662561153	-2.94421108722582	3.19032526594997
C	-5.65364618419297	-0.86902022587497	2.84109405956931
H	-2.72360477848415	-6.42574668174268	2.16881760616646
C	0.41060431550825	-6.79547849477291	4.55411523155359
C	1.12158529520284	-6.48610478970079	2.15912747807706
C	-0.81402169881520	-7.96078906609292	2.72810545081994
C	2.93935232521713	1.78621777674952	1.90693632069617
C	2.37864781441216	3.66279090350338	3.25734577019022
P	-1.10534658597600	-0.13174763070625	0.62279012114000
C	-5.11530038763357	0.42746000471460	2.56843330142688

C	-3.05084416899325	1.88598573695045	2.23601151071756
C	-7.05164335568446	-1.01169772564448	3.01787441249637
H	-0.43385131073791	-6.89300506307493	5.24421404492574
H	1.04335647432586	-7.68376340865693	4.65424490926530
H	1.00225630956079	-5.92886245154714	4.86044062842051
H	1.68271503283411	-5.57376212665157	2.38255044775925
H	1.80373358907472	-7.33810904153752	2.25263633942159
H	0.79726724209560	-6.43074743451449	1.11482134068931
H	-1.14971268929567	-7.95708766186080	1.68563766037070
H	-0.13128985664200	-8.80766036360669	2.84526958425133
H	-1.68139384540146	-8.13636858057798	3.37331032117379
O	2.82931450834520	0.42300584042537	1.65172324869859
C	3.72815538612020	2.53536309927263	1.05583237611263
H	1.88262092145114	4.10256969825586	4.11766175768789
C	3.14844277722318	4.49750465530245	2.41975291055771
O	-2.07124841574034	1.09584806297946	0.22076380513993
N	0.38222981838183	0.27598449273440	0.76365090444795
N	-1.56070961322445	-1.32397303620315	-0.40637596388443
C	-6.00860569941442	1.51697889880353	2.43888702947558
C	-3.17659547972696	2.91629750197011	3.21993702611491
C	-2.22650826123094	2.12342352978352	1.15448551970119
H	-7.44959662558773	-1.99960732516398	3.23027917112513
C	-7.89035240021576	0.06925991039892	2.90105655487682
P	1.88242935501435	-0.04926646900085	0.42423658511624
C	4.37315828502487	1.88756316549663	-0.11336783303756
C	3.82748188257387	3.94149736524953	1.28947973076588
C	3.23134629333942	5.89177358571843	2.65767651824562
S	-2.41775694904020	-1.05428658509468	-1.80474255659866
H	-0.99475986670691	-2.56304211987992	-0.36466777933269
C	-7.36249204286373	1.34112479682018	2.60031306619383
H	-5.61433361821103	2.49679726625880	2.19628381438448
C	-3.89814246365296	2.74836562136227	4.42644853140800
C	-2.48903301773592	4.15378615127701	3.00844685355266
C	-1.52317437429147	3.32980819861767	0.92153222657969
H	-8.96070379445653	-0.05490197118577	3.02946060630026
O	2.24593901530187	1.02181536307058	-0.73353648924143
N	2.33023772311991	-1.51829965352155	-0.00820884100002
C	3.59990736486419	1.17916217588884	-1.01630476277292
C	5.78052254124175	1.95896144317536	-0.34059210925908
C	4.51813771609477	4.82158652573701	0.42147600627933
H	2.72075944431842	6.29926011069439	3.52561496322793
C	3.92568703612511	6.71433332860381	1.80614626324158
O	-2.42165276011533	-2.34590860922206	-2.50678523098338
O	-1.96469266107275	0.14988464738544	-2.51425282167278
H	-8.03071926265172	2.18780434933661	2.48351504234586
H	-4.39451800320206	1.80494287732725	4.61846262079826
C	-3.96855454790897	3.75801004312382	5.35667923348110
C	-2.59848639386414	5.17962830489023	3.97944569238880
C	-1.68948576581955	4.32818159152188	1.85948492752612
C	-0.66622931634553	3.53419473155932	-0.26198519307681

S	3.15354097858858	-2.54348236485029	0.93829552717905
C	4.09362517386603	0.61846964816788	-2.22214283333457
C	6.30815811805528	1.40864734308345	-1.54988176231680
C	6.68037670921620	2.50959954157243	0.60211943858357
C	4.56575374734611	6.17219538711056	0.67330649435941
H	5.01146631278001	4.42022206035152	-0.45571585269009
H	3.97628412299217	7.78127452714580	1.99661697339287
H	-4.52277559959697	3.60213343421110	6.27655464064918
C	-3.32353524601474	4.99030685401384	5.12917655817239
H	-2.08258819584918	6.11840878917368	3.79917481215051
H	-1.19359511700688	5.28210261214501	1.70586344789743
C	-1.05770780893457	3.13400149152541	-1.54088097844995
C	0.55325893494283	4.21005116600614	-0.13575370219427
O	2.71988031195208	-2.53299016289049	2.34912482250295
O	3.18370452805412	-3.84641746548849	0.23843179748668
C	5.44213008367788	0.78535600225245	-2.47121657131323
C	3.22971050906610	-0.07028957605562	-3.20420576436065
C	7.70191379906584	1.48205194552468	-1.78944207459105
C	8.03137011905478	2.54257820452113	0.34943638556157
H	6.29366696363764	2.89242414889975	1.53916451781499
H	5.09823005871971	6.82698569911706	-0.00898214272161
H	-3.39338759681727	5.78164264627403	5.86808908676691
C	-0.27668164785456	3.42301349494912	-2.65393325304955
H	-1.98934646932249	2.60280335187657	-1.68257808677237
C	1.32496508142715	4.49927062197768	-1.24943761523615
H	0.90741165346201	4.50002389277797	0.84863056294695
H	5.85835478375395	0.42394499851917	-3.40569548014290
C	1.91750244465356	0.34293300608042	-3.48160988668807
C	3.74479207996702	-1.12726661464518	-3.96265596945541
H	8.09184137052121	1.06762644037671	-2.71439774805420
C	8.54779855548632	2.03613798074534	-0.86053099307807
H	8.70640674435964	2.95364231257475	1.09284727860920
F	-5.74415373702994	3.00817015036341	-0.51336721962080
C	0.92533748050385	4.12520400530996	-2.53941251952231
H	-0.63430711920191	3.09461390210529	-3.62363864355698
H	2.25909110607607	5.03099855215802	-1.10120964526463
C	1.19029824541909	-0.24368401638177	-4.50566405907394
H	1.46339738276068	1.14559378681064	-2.91369946657734
C	3.01366290223110	-1.69835145464587	-4.99926151339440
H	4.73706691582299	-1.50996800741637	-3.74342723516918
H	9.61572443370321	2.07292333730947	-1.04967613651333
C	-6.55517612606580	2.09513923189433	-1.09908819266825
C	1.77297860128172	4.52869893839312	-3.74692338296390
C	1.72647418565533	-1.25496994529283	-5.31314673245200
H	0.18166672140383	0.11730930760884	-4.68289382959244
H	3.47157945720593	-2.49847386219602	-5.56971324219809
F	-6.69073044220490	2.47181123412852	-2.39532003708567
C	-5.98527818004283	0.70690146026542	-0.99325332747887
F	-7.77134204914666	2.20067115114424	-0.52591543652563
C	1.21303376089468	3.97157830891317	-5.06139200722600

C	3.21481861273845	4.01685090643300	-3.57932529398428
C	1.79006773984381	6.06743394547667	-3.83520246036321
C	0.92991645899191	-1.77507388233927	-6.51119551861446
C	-6.78060950814651	-0.36453773346726	-0.60440449274422
C	-4.65066813206064	0.51768991307981	-1.33915048231492
H	1.85410006651924	4.28337030126156	-5.89188086126119
H	1.18508681477094	2.87675968740251	-5.05552317125386
H	0.20345651686595	4.34549451325371	-5.26254439231272
H	3.24039957361425	2.92414011807789	-3.54206315875861
H	3.82789634249192	4.34225773847958	-4.42692169798315
H	3.67708936361763	4.39847843223545	-2.66422171670622
H	2.22577395852376	6.51502371766013	-2.93683206254069
H	2.38208450018052	6.38963260274537	-4.69913201395388
H	0.77393157283275	6.45859221293667	-3.94698612458123
C	-0.43132086318541	-2.32516317312457	-6.04956387481947
C	1.66620996854058	-2.88765185922744	-7.26646089649906
C	0.70473736277841	-0.60099844121860	-7.48478394000905
C	-6.22445573983322	-1.63902325895884	-0.55482757737266
H	-7.81642194296321	-0.20780239086810	-0.33291416288471
C	-4.12115429240685	-0.76221313279485	-1.27614204816017
H	-4.03517343224905	1.34662831822108	-1.66835547445180
H	-1.00532475768609	-1.58922370981407	-5.47920256122377
H	-1.03086807009012	-2.62391370801551	-6.91663368840504
H	-0.28655333537648	-3.20819059951251	-5.41908838010501
H	1.05288196889567	-3.22085198767744	-8.10952420804735
H	2.62289051349952	-2.54034607348988	-7.66960643761183
H	1.85182840469141	-3.75856251283832	-6.62887170232877
H	0.14689346947516	-0.94136374909228	-8.36448509867577
H	0.13765176023059	0.20697411137676	-7.01475313730688
H	1.66274579162012	-0.19051043331545	-7.81981346417285
C	-4.88964024238537	-1.85142728427147	-0.88665883445369
C	-7.06826613006740	-2.83127353983333	-0.19545925824331
H	-4.45907463424834	-2.84778075720281	-0.86625074389896
F	-8.29745620951647	-2.48404205212449	0.23605489010835
F	-6.49202761132911	-3.57801408030516	0.78086387326143
F	-7.23128386255038	-3.65222026923913	-1.26059185032667
C	5.65290207405227	-2.22094601578273	-0.20179286183237
C	4.86628991887018	-1.95556967186308	0.91121889225759
H	5.24117498646567	-2.77285405836653	-1.04017210922310
C	6.97517103160895	-1.78694078778849	-0.20679035779869
C	5.37039216786056	-1.26816807616736	2.00410030767527
C	7.50178481761690	-1.08294940212663	0.87201798454317
C	7.84192991113268	-2.14882224776570	-1.38022404642124
C	6.68929799452021	-0.82460815313299	1.96945188557773
H	4.74160934794655	-1.09133900318412	2.86846627322807
H	8.52566135939123	-0.73333063674238	0.85244296181269
F	7.23401919866591	-1.86624600715149	-2.56205374283863
F	8.11281942729043	-3.47689423411006	-1.39793634745265
F	9.02493632404657	-1.50087662506516	-1.37297930212800
C	7.21516599700089	-0.08662734567925	3.16852251637242

F	8.44791112641323	0.42362314206722	2.96509532351377
F	6.40130775822001	0.94165374654735	3.51364443121495
F	7.28983149616484	-0.89909955967525	4.25155818235744
H	2.17900676565739	-5.33630811475746	-0.57772761040489
H	2.93289437785541	-6.09424584424733	-3.08189167371483
H	0.69699426786462	-5.39442651067447	-3.75129976929756
H	1.24772933878288	-2.91303878689025	-2.01181273188357
C	-2.42396830898369	6.99670050011520	-2.93524095453741
C	-3.41242601797011	5.84865175948321	-3.15086371020490
C	-1.88791936794714	6.99956642259726	-1.50107753780720
C	-3.03481919459474	7.05142576010735	-0.48670735075610
C	-4.01890913786599	5.89897180177941	-0.70461196850135
C	-4.55784635982044	5.90373082048959	-2.13732989375294
H	-1.59445920962073	6.91628746010527	-3.64807873621126
H	-2.92727220946210	7.95288999257795	-3.13904195184570
H	-3.80487330152684	5.86734713287907	-4.17498042597979
H	-2.87730849705819	4.89673164983251	-3.03127088348055
H	-1.30222675860286	6.08520124742857	-1.33726573280954
H	-1.20612803088011	7.84537731802298	-1.35054575802256
H	-2.64104709833125	7.02567190941381	0.53701330334389
H	-3.57013928112529	8.00590768725417	-0.59439380651368
H	-3.50252994437919	4.94697649007518	-0.51682274353898
H	-4.84350605498049	5.95867646145932	0.01582589499317
H	-5.24180216718719	5.06078812415058	-2.28916143293019
H	-5.14186853285735	6.82090963573538	-2.30077680141778
C	-3.03941544194069	3.02113474434066	-5.69661826128379
C	-2.15953548429271	1.93761222465532	-6.32483634991310
C	-3.87747967171580	2.44427013215501	-4.55290776877375
C	-4.72062961535235	1.25727079281549	-5.02557447930191
C	-3.84687857670942	0.17808101473168	-5.66998897644845
C	-3.00564233834763	0.75782507042707	-6.80993081581212
H	-2.42359370361866	3.85282865411686	-5.33311349601955
H	-3.70627503159157	3.43635970455733	-6.46583157912387
H	-1.56659748211285	2.35290835554254	-7.14898340011637
H	-1.44602325746478	1.57981657290607	-5.56711648952509
H	-3.19396289609097	2.10231381508706	-3.76295011326477
H	-4.51755356135807	3.21965938612991	-4.11639198533791
H	-5.28606147073740	0.83475339771509	-4.18651859755508
H	-5.46187839767894	1.61142306711183	-5.75624492028808
H	-3.17892220142059	-0.24232998171920	-4.90566208362316
H	-4.46888385652048	-0.64659333832092	-6.03791182332033
H	-2.36667754754520	-0.02025340635720	-7.24438300169106
H	-3.67207311861226	1.10337953761870	-7.61334848916637

282

Major0 3 CyH

C -1.65904178101607 -3.06194016714723 2.51718790288928

C	-0.90874033261658	-3.98433583100368	1.61167289671529
C	-0.52875754207387	-5.32788622981706	2.24824833106049
C	0.57741482617759	-5.15327360154630	3.28235900566859
O	0.22769476028012	-4.14223732578953	4.23037467776067
H	-1.55983574702845	-4.16611249701875	0.74493208840836
H	-0.18145018940263	-6.00717169093777	1.46048644970109
H	1.51742495760943	-4.89060997914495	2.78048286328040
C	-2.86868566298937	-3.54020199596534	3.19502142193541
C	-3.19155556122343	-3.03928551910119	4.46363636842296
C	-3.71975132080102	-4.49070948013008	2.61162055128049
C	-4.33718772959487	-3.46060366267522	5.12321306211389
H	-2.51352108560071	-2.34191735483019	4.94112361329687
C	-4.87411229627306	-4.89702999169382	3.26406295198571
H	-3.50167233729802	-4.87273345902163	1.62202786186813
C	-5.18732842122762	-4.38455178189843	4.52131192295403
H	-4.56411979229722	-3.07403898814893	6.11200075013068
H	-5.53506869168423	-5.61386525796175	2.78761697014092
H	-6.08776983735945	-4.71023434522348	5.03220660277041
C	-1.31054715228713	-1.72370171081419	2.59660194040314
H	-1.83613478107763	-1.08492763425704	3.30161037874526
H	-0.27489664198881	-1.46316954537288	2.38838807747374
H	0.16126655258704	7.48850847208113	1.40289343589718
C	0.48665652649696	7.13772109382719	2.38706078168390
C	0.07058299025563	5.67290310458882	2.62978046942987
H	0.03950339783002	7.78921422773463	3.14614118003659
H	1.57526556866331	7.24264682491525	2.43574445855341
C	0.75744589573384	4.80192028987339	1.57659114027518
C	-1.45932528983234	5.55615086960148	2.50788941367032
C	0.47445976893970	5.28435963011130	4.05725570429731
C	-1.32873444305903	1.78477137997778	3.36691376370159
C	0.48598997729054	5.00677804765674	0.21749435987155
C	1.70570802894274	3.82721029345322	1.89567728460635
H	-1.80637481926793	5.81655533716070	1.50369186433036
H	-1.79380003409530	4.53810469010478	2.72784784014935
H	-1.94471135405769	6.23317715626850	3.21946109393655
H	1.55123921310437	5.40284151758009	4.21786844224023
H	-0.04224110809348	5.93242464967963	4.77199353922431
H	0.20007526823056	4.24956476279749	4.28852992296710
C	-2.70459329902388	1.66907591858869	3.11247313243592
C	-0.77181545543928	1.24514634609856	4.51486220422212
H	-0.68215773706649	2.30522319528219	2.67126719476019
C	1.15116393659527	4.29926533338967	-0.77053297607250
H	-0.25625077193401	5.73842821009482	-0.08562890339747
C	2.37987632795560	3.11658932712954	0.90871742739369
H	1.94933307679535	3.61321951449044	2.93054925526164
C	-3.35801964771637	2.30158533160154	1.94757516072322
C	-3.47861988265171	0.98060313094633	4.05391398887497
C	-1.54500877503329	0.57544826151889	5.47220172659764
H	0.29968810631177	1.34699631819926	4.65463681431770
C	2.12881823830184	3.35060268799024	-0.44571879734619

H	0.89812784567258	4.47419126220857	-1.81156217727251
H	3.11937522875112	2.38543407403646	1.20797800505135
C	-2.73350631390825	2.42078801452157	0.67908220516802
C	-4.62009513083589	2.85001199689882	2.06739159434071
C	-2.91229283088083	0.45681585253026	5.21173584039300
H	-4.53910333430379	0.83380955623200	3.87414046817681
C	-0.87659891646244	0.01215006945658	6.72531091623863
C	2.88019455102789	2.67728364194453	-1.52376455134073
O	-1.46037074737564	1.87471315725667	0.53325995360758
C	-3.28948757252545	3.08719785179260	-0.39853582990906
H	-5.11571148716297	2.83114934377571	3.03178866284636
C	-5.29664740069750	3.43217300612646	0.97732535507978
H	-3.55876739251070	-0.06676900772091	5.90676646605521
C	-0.25147655276042	1.17799230073414	7.51565573968331
C	0.22253589963418	-0.98481041243799	6.31030370811256
C	-1.86676274335392	-0.71034137419012	7.64606560169093
C	3.20854901648629	1.30034353699620	-1.47712423860785
C	3.29055430133251	3.36954215687449	-2.64505962308775
P	-1.28352287798765	0.57541724934882	-0.40884767822291
C	-4.63442829569360	3.55286948932385	-0.28340675003821
C	-2.48959156765469	3.30460590971409	-1.62827679193773
C	-6.63142137210717	3.88960503337756	1.10204673954029
H	-1.02194259881782	1.89374450389730	7.82063653752038
H	0.24490976729994	0.79898981220230	8.41564128426634
H	0.49171053144273	1.71545490148877	6.92098877902384
H	0.97179182293521	-0.52126359883418	5.66241439394426
H	0.73671451279657	-1.37045317896514	7.19731608485608
H	-0.20557117966657	-1.83709053281495	5.77170860753737
H	-2.33059714940705	-1.57234963602447	7.15464716682783
H	-1.33369758561424	-1.08543630732923	8.52489103266202
H	-2.65853546303844	-0.04071818130557	7.99834214413591
O	2.82596296009750	0.58009340182864	-0.35050021913847
C	3.87923552458135	0.61840831839061	-2.47311914324844
H	3.08420654245975	4.43339962507690	-2.71342774024421
C	3.97571979241140	2.74322599520840	-3.70733600834728
O	-2.12391210289430	0.96151926983643	-1.72987641232955
N	0.24327398978582	0.47962071737436	-0.65805013070628
N	-2.11070805714227	-0.69426802481823	0.21770498619212
C	-5.35295879573608	4.09184966292208	-1.37677835976322
C	-2.22825652982202	4.61606923250986	-2.13301773579867
C	-1.90855666773016	2.23237707697392	-2.27274463827585
H	-7.12443032269058	3.79746486076496	2.06542662416895
C	-7.29827792654901	4.41667147166928	0.02348023490871
P	1.53777440418171	-0.39402307235416	-0.47026413865136
C	4.09209118561880	-0.84496791759509	-2.34276713572189
C	4.26849084984168	1.34355725680359	-3.64143499430166
C	4.35155735804393	3.47389681085477	-4.86153626671911
S	-3.23962867713576	-1.57187023647289	-0.62618291532736
H	-1.76495621274861	-1.26581743467755	1.39672736834776
C	-6.65375757041955	4.51033387306022	-1.22680524804268

H	-4.86985123591113	4.15960009233244	-2.34447060285708
C	-2.67595716212242	5.79409761010671	-1.48787143444738
C	-1.42486988918811	4.74776960476516	-3.30945845871652
C	-1.10870495607556	2.32635740410702	-3.43752393355945
H	-8.32427555079721	4.75413851353252	0.12856060312210
O	1.76211289671157	-1.08260738931548	-1.91692739375112
N	1.63480125113374	-1.48982701023544	0.68634103772976
C	3.00862404148067	-1.67528455832923	-2.11102600968909
C	5.38755985308360	-1.43756796439386	-2.43418515036743
C	4.88058964128294	0.73327854973322	-4.76257661150519
H	4.13627266762373	4.53829836361413	-4.89209655455316
C	4.96204352718958	2.85403902252982	-5.92301399810947
O	-3.56798112360503	-2.70775985209350	0.24830219304500
O	-2.83580157700137	-1.82712939156762	-2.01459184905992
H	-7.19178534427967	4.90638307708141	-2.08157374289950
H	-3.26060850112638	5.71051971408800	-0.57980093079139
C	-2.37039553433213	7.03605386870612	-1.99174214255075
C	-1.14228515184245	6.04285870729182	-3.80961629261092
C	-0.90240345826223	3.59689965705293	-3.93709780605682
C	-0.51825967179627	1.14443743328868	-4.09648038865790
S	2.47734359952707	-1.28598056483537	2.05352841208807
C	3.07965990103394	-3.09189542387331	-2.07376753588923
C	5.49179269611759	-2.86305053061580	-2.41032070572450
C	6.57607350645180	-0.67237978788179	-2.49860659318723
C	5.21823447249420	1.46884655605945	-5.87369082606882
H	5.08223525655131	-0.33074129146636	-4.73990637657211
H	5.24070303324906	3.42429075197143	-6.80298510629952
H	-2.71878383858367	7.92521015978946	-1.47627049534073
C	-1.60404582398100	7.16472496585012	-3.16766483068037
H	-0.53982236813784	6.13018113707481	-4.70925840157697
H	-0.31722902277449	3.71647414332659	-4.84414857165361
C	-1.23555401582189	-0.03735545058848	-4.29517516225792
C	0.78797061268475	1.20592964578470	-4.59711250492036
O	2.37698672367841	0.07815450491114	2.61111689960147
O	2.14174669708796	-2.41793518780239	2.94472494092390
C	4.33000797030137	-3.64799341122546	-2.26437951254015
C	1.89276095556825	-3.95504757032759	-1.89093025012760
C	6.77144670095179	-3.46136954349190	-2.51175648426253
C	7.80469934662010	-1.28489819236305	-2.57262046934086
H	6.50915961994687	0.40907758192070	-2.47340424318356
H	5.68373540974893	0.97685116394105	-6.72161649874967
H	-1.37393442797631	8.15080298263968	-3.55731525852772
C	-0.67629638088496	-1.10549623628533	-4.98763839496993
H	-2.24562321575091	-0.13302002534263	-3.91892716837400
C	1.33712810557592	0.13905163024323	-5.29029743086600
H	1.38794298163342	2.09396090817379	-4.42318216617324
H	4.43169087659331	-4.72754462189224	-2.30043812087422
C	0.63126740345494	-3.63623375832885	-2.41709424885066
C	2.02177511243177	-5.18510783118834	-1.23609438908355
H	6.83988824377517	-4.54512943750957	-2.49976506273260

C	7.90510417459364	-2.69082893064999	-2.59174083236532
H	8.70478635307247	-0.67986565514452	-2.60574517559564
F	-5.74447032262240	1.94277655707616	-3.88777323147901
C	0.61653496162502	-1.04140640980007	-5.51292178368771
H	-1.27891127395388	-1.99649864985817	-5.11959175711265
H	2.35264039794401	0.23735722931142	-5.66017089366195
C	-0.42403502758483	-4.53170905462586	-2.32956146983750
H	0.46979088409248	-2.69222108286180	-2.92185581800255
C	0.96406381229601	-6.08620870364802	-1.17200108831376
H	2.96650357070826	-5.44752164350761	-0.76908221687810
H	8.88053296437333	-3.16214302410572	-2.65483518578193
C	-6.74879478407408	1.28134575618912	-3.26578943857554
C	1.23325882590586	-2.16796616705813	-6.34458234472953
C	-0.27963546318697	-5.79308012542121	-1.73795479853498
H	-1.37852601115670	-4.23185121824787	-2.75137387979044
H	1.13056513276636	-7.03593981462268	-0.67676917592552
F	-7.13358524600854	0.28339687669478	-4.10000798358434
C	-6.30878487613822	0.72909890633090	-1.93804648795952
F	-7.79101714614625	2.13043665762058	-3.15374339656742
C	0.33823237389349	-3.41217135885653	-6.39621066256479
C	2.59507343229179	-2.58132984346959	-5.75839460834895
C	1.43791706849719	-1.65124918818198	-7.78238714434862
C	-1.44182486731827	-6.78851253619646	-1.76842606111353
C	-7.12206362700817	0.83551576981378	-0.81611378908382
C	-5.10308167995482	0.03687791533976	-1.88268518862129
H	0.83252019753178	-4.19051758982571	-6.98602891002492
H	0.15469207432989	-3.81776150306695	-5.39543031566891
H	-0.62499188298282	-3.19996329396527	-6.87119650749704
H	2.48338171228819	-2.96889210948040	-4.74176782286065
H	3.04334562262123	-3.36723860824075	-6.37596983979683
H	3.29422465773089	-1.74114564337354	-5.72672362619065
H	2.11082840131968	-0.78853218380068	-7.80336711004280
H	1.87251187854895	-2.43869207111750	-8.40835511735245
H	0.48341333015457	-1.34608184643794	-8.22325504509145
C	-2.62663846909889	-6.24371395686114	-0.95119257126592
C	-1.05146329956739	-8.15671502522789	-1.19707443391659
C	-1.88762188874180	-6.98922966071766	-3.23067450158827
C	-6.71236246169231	0.24910349031373	0.37740206413044
H	-8.06268567407036	1.36762374257065	-0.87046307554359
C	-4.72019440568519	-0.53853536390598	-0.68127892394157
H	-4.48233232063948	-0.08104525075254	-2.76289666186481
H	-2.91635099716259	-5.23655410959015	-1.26616758646161
H	-3.49721101905526	-6.89925434448446	-1.06764014360995
H	-2.36911691794455	-6.21293934866630	0.11254676020285
H	-1.90817486984624	-8.83499062959421	-1.26031394531188
H	-0.22523473642103	-8.60721130127726	-1.75671984129481
H	-0.76313954335247	-8.09046681605499	-0.14262249130831
H	-2.69172171443873	-7.73162262925674	-3.27730226444912
H	-2.26281140878078	-6.06143887317394	-3.67097478668028
H	-1.05333799314346	-7.34363236522177	-3.84457605989328

C	-5.50573431507775	-0.43975027029320	0.45982324913612
C	-7.58779935933855	0.30278142059860	1.59975162382754
H	-5.19502568276488	-0.91760872485687	1.38427197779546
F	-8.72263224403715	0.99949162682074	1.38982629374531
F	-6.94980654300020	0.87968452663594	2.65091842215297
F	-7.94214760212773	-0.93956109551009	2.00455787282702
C	4.66767640198320	-2.84622641457980	1.42396161255783
C	4.20933764675925	-1.54704738678900	1.59547259785474
H	3.99530788757331	-3.68500765157869	1.56921397466205
C	6.00265882253388	-3.04675576852346	1.08582894200374
C	5.05066462574980	-0.45708397822019	1.43423751570465
C	6.86492926205285	-1.96978435579246	0.90498944165936
C	6.50405034007586	-4.45947580923840	0.97564798605118
C	6.37716670013579	-0.67953288818581	1.07636525421983
H	4.67514952611887	0.54675399022755	1.59439200448257
H	7.89672916014127	-2.13479393672878	0.62355889442413
F	5.70014640801126	-5.21896724416903	0.18621597279394
F	6.53025105524291	-5.06515620285981	2.18824257210914
F	7.75083919303185	-4.53210688091498	0.46687740904840
C	7.27310389934739	0.51633703974214	0.91519751661971
F	8.47190234297920	0.19878150407597	0.38312830304633
F	6.70799183330895	1.45379804645297	0.11519327528594
F	7.51077923479396	1.11825563955292	2.10781448719273
H	0.76134333134816	-3.36412140960221	4.00905090367503
H	0.73194491213906	-6.09368353475730	3.82723116546353
H	-1.40553797010802	-5.78510738472657	2.71973320096786
H	-0.01809264904166	-3.47576786434265	1.22777386063518
C	-7.04668160399753	-4.03103905881677	-2.76828729711681
C	-7.72378588520718	-2.89194014230597	-2.00327703877263
C	-5.88242900308098	-4.62003896359320	-1.96714144980920
C	-6.33493685236254	-5.07355419831312	-0.57641223507281
C	-7.02243572274421	-3.93691660091090	0.18390261533604
C	-8.18697918335206	-3.35428773974148	-0.62025677011608
H	-7.78730067879971	-4.81963357472107	-2.96505294042605
H	-6.69410265648236	-3.67664393579688	-3.74403266230499
H	-7.00772497017591	-2.06723484670230	-1.88352129879868
H	-8.56809267738313	-2.49389544379169	-2.57893215072318
H	-5.43411140692788	-5.45926221927928	-2.51317608665797
H	-5.09503773483953	-3.86152522035259	-1.85779214561792
H	-7.03406180645761	-5.91609070606347	-0.68081980079362
H	-5.47357036899395	-5.44026249917715	-0.00424964294787
H	-7.37407083958733	-4.28708747492692	1.16190002396324
H	-6.28203672237100	-3.15003330727337	0.38067754815783
H	-8.96552522168790	-4.12167174008693	-0.73882822453344
H	-8.64578077778369	-2.52233026311574	-0.07343960637671
C	4.83212894435969	6.69782062297641	1.80693097789052
C	4.17472852258480	6.56516050928535	0.43067219586026
C	5.47124835841654	5.37511889198257	2.23593799197982
C	6.48397937801482	4.88746714946619	1.19761370254247
C	5.82983984536289	4.75094884759892	-0.17912632040293

C	5.17900908322706	6.06739798261135	-0.61316906356754
H	5.60511940441551	7.47901496821380	1.76970997210625
H	4.09356047917289	7.01942563477688	2.55187972525681
H	3.34297002879499	5.85190701881455	0.50127310045014
H	3.74435519600646	7.52467130095637	0.11835783986739
H	5.94811817834369	5.47882531849147	3.21825215132284
H	4.68064240101251	4.61939586417427	2.34381564919182
H	7.31319956372096	5.60694068135030	1.13502725466327
H	6.91783820366130	3.92983698819284	1.50809428691199
H	6.56591143625047	4.42505560424924	-0.92378052439780
H	5.05969994006741	3.96887464440369	-0.13463682670250
H	5.96165648017903	6.82688340891891	-0.75527026274920
H	4.68466324596009	5.93961251670197	-1.58401921318599
C	4.78186146167353	2.20863557773893	4.16683637159877
C	4.28198652117753	3.23788589597568	5.18331580535153
C	5.25938433685663	0.93234909595383	4.86443008363479
C	4.15841929391384	0.34184842505032	5.74877767776392
C	3.64757201336596	1.37091227196463	6.75987024406417
C	3.17339032986704	2.64639420105520	6.05780366465275
H	3.95570444643110	1.95061496115447	3.48902004881027
H	5.58701408337162	2.63451212684480	3.55664562220293
H	5.11871371542511	3.55270796925924	5.82332563319831
H	3.92417645656219	4.13924175337517	4.67085596476136
H	5.58227438458930	0.19214242052999	4.12294546227331
H	6.14008445263706	1.16544978598230	5.48015395832512
H	3.32766405420605	0.01985464627224	5.10684839373960
H	4.52474801278482	-0.55162504583682	6.26808924870242
H	2.83752048122725	0.94076732361092	7.36091517879350
H	4.45691127532832	1.63014623446766	7.45746293943560
H	2.31169111305404	2.40680229281471	5.41626858838647
H	2.82668800165299	3.38444670924066	6.79156701302058

300 Major0 4 CyH

C	-1.52475644515674	-3.65440794038616	1.62126439566657
C	-0.82548049645891	-4.28055805322075	0.45848642270870
C	-0.35196355468387	-5.72158931036355	0.69706603733775
C	0.82930388778907	-5.77296276931512	1.65937384353904
O	0.53090616016997	-5.06692096016020	2.86624762078320
H	-1.54675057422327	-4.27398285108786	-0.37147625794839
H	-0.05160775498826	-6.15801410627798	-0.26368474453635
H	1.72062249603953	-5.34592304633718	1.18300785552453
C	-2.65775394186400	-4.35133083656488	2.24023786190229
C	-2.89425880803739	-4.20279734271267	3.61391527332221
C	-3.51935789391036	-5.17011943610082	1.49528972831062
C	-3.96721149947425	-4.83810585410600	4.22234404522982
H	-2.20457582429725	-3.61260803691352	4.20571969543498
C	-4.60221462276450	-5.78947315613797	2.10117516017139
H	-3.36780476122892	-5.28097172003918	0.42873556018134

C	-4.83038458028711	-5.62645499547553	3.46597083559875
H	-4.12636892533386	-4.72559352386381	5.29029810486841
H	-5.27330089430226	-6.39923163504477	1.50505976663123
H	-5.67424833243261	-6.11844749106786	3.93883904437208
C	-1.21498026631056	-2.36342235607581	2.01584620670752
H	-1.70007351700584	-1.95369953492644	2.89821957311696
H	-0.21032291922743	-2.00536845464226	1.80419813853097
H	-0.23555380739548	7.03051115981621	3.15604705390579
C	0.07727480503154	6.43650892461553	4.02019573041994
C	-0.31919437945525	4.95566561267084	3.86010208337660
H	-0.39230091872664	6.86006747450458	4.91510795703203
H	1.16344425584440	6.53247369697701	4.12130915108580
C	0.35955702354001	4.41329565807434	2.60069928320650
C	-1.84851657539836	4.84863477318427	3.72597127358723
C	0.10712462203099	4.20387848209895	5.12662495180838
C	-1.29096428946498	0.88893817766094	3.68353508208091
C	0.03876159349244	4.94861370325267	1.34661024984815
C	1.34026137644007	3.41984617731319	2.63305705891607
H	-2.21784779705652	5.40579471948841	2.86072450725056
H	-2.15862085416968	3.80514557161477	3.61564172380368
H	-2.33317881919110	5.25496963489374	4.62046258867055
H	1.18201765422507	4.29837152383962	5.31052526365336
H	-0.41466986522193	4.62539850723274	5.99158712362629
H	-0.14760280541134	3.14036250237884	5.07226889544679
C	-2.67716990752483	0.72360816987138	3.53457828518746
C	-0.57425879047707	0.11755382253854	4.58398799764852
H	-0.76158781514539	1.62980475091531	3.09761341450018
C	0.68842967191142	4.53402679062241	0.19572503726951
H	-0.73290101886976	5.70653017336610	1.25601698143608
C	2.00487746034326	3.00402904680943	1.48296539690007
H	1.61606758445496	2.95999765340109	3.57479051210185
C	-3.48438217440707	1.56764033534475	2.62889450600377
C	-3.30074454553913	-0.24302032588112	4.33132188069273
C	-1.19481936243053	-0.84319827013272	5.39332347948041
H	0.49980403923207	0.26847570811893	4.64040677055100
C	1.70433002537143	3.56946623227594	0.24027833622322
H	0.39401884998048	4.95640161830270	-0.75990908534793
H	2.77232192969468	2.24416173850833	1.57040210451979
C	-2.99408719191951	2.04766879198774	1.38657954002615
C	-4.75843036219008	1.96563874548588	2.98423902286616
C	-2.57601195010690	-0.99821712780210	5.24841185961077
H	-4.36527801913533	-0.42625562499229	4.22125857895375
C	-0.35366504605802	-1.68006266712900	6.35648678547870
C	2.43540108570449	3.24048248601533	-1.00028875737820
O	-1.71281598156000	1.65632222910441	1.00720665462522
C	-3.68525445418293	2.92053806679409	0.56540279260686
H	-5.15710799728092	1.66728554524056	3.94782808877145
C	-5.56825016260995	2.74746136465079	2.13612347233086
H	-3.10862337499281	-1.73289736531966	5.84137395196164
C	0.40904811003279	-0.74157583943310	7.31050821189083

C	0.65261841670751	-2.51774786381316	5.54336174588228
C	-1.20720166289361	-2.63132240908146	7.20313438161613
C	2.82780867019477	1.92203821277404	-1.33975984480556
C	2.75433698165650	4.22920233647556	-1.91060902853552
P	-1.54992065535216	0.66178102630963	-0.25376694718835
C	-5.03720510672971	3.23251458048853	0.90081102836009
C	-3.00999361881141	3.51641284086806	-0.61313857250363
C	-6.90733197892034	3.05695742252328	2.47876334763488
H	-0.28777804367545	-0.12884440275899	7.89177132231117
H	1.01773508657432	-1.32899263314828	8.00633890351389
H	1.07925702576369	-0.06999699592074	6.76826532115059
H	1.28800016097860	-1.89298380704605	4.90801532787379
H	1.29967486573326	-3.08646202731946	6.21986774411421
H	0.13055327543722	-3.23205590997256	4.89779940893631
H	-1.73899626798209	-3.36320244993193	6.58558885141136
H	-0.55803481904778	-3.19168922964042	7.88257675470672
H	-1.93883833593521	-2.08830423530320	7.81088457221295
O	2.54528599903397	0.90321205510715	-0.43459491364722
C	3.46058748438796	1.57150441074322	-2.51654318752665
H	2.50521519485913	5.26034474079825	-1.67943146320522
C	3.39833351493332	3.94988676787736	-3.13355316841470
O	-2.51775613591672	1.31895572553922	-1.36412218095168
N	-0.04676531540419	0.74887050005524	-0.62397208994414
N	-2.25272599306285	-0.78499410348320	0.06700910689228
C	-5.88478183341631	3.97044129001840	0.04137805608079
C	-2.86880097001699	4.93151457723199	-0.76173494200355
C	-2.41532688568127	2.70147829759279	-1.55407205234063
H	-7.30048583548079	2.69010822903047	3.42239237274838
C	-7.70125038578456	3.78560353398707	1.62735642629877
P	1.29028691958910	-0.06818100183146	-0.76174011558738
C	3.72805640394292	0.14190498856552	-2.81215358865887
C	3.74659669744828	2.60240254688532	-3.46443179672792
C	3.67430573359049	4.98381561681756	-4.06216029980098
S	-3.40662944273126	-1.48517409509527	-0.90061612729042
H	-1.78343026110991	-1.63081285502636	1.01706752176821
C	-7.18607639493842	4.23706682462174	0.39509237110740
H	-5.49974003592199	4.31176421778462	-0.91249535513865
C	-3.34861863660475	5.86068318808265	0.19238823503090
C	-2.15737963758642	5.43131407445877	-1.89792043776433
C	-1.70598888705135	3.16248990921882	-2.68983339643457
H	-8.72884595475239	4.00741616655938	1.89646674429158
O	1.43943457606663	-0.31113634326037	-2.35369325875192
N	1.49857009266172	-1.43779662627982	0.02872540251517
C	2.68580889461660	-0.76869422657059	-2.77483765584084
C	5.03044409556706	-0.33609945092974	-3.14352291581914
C	4.30613451371870	2.34905171088794	-4.74017658148960
H	3.41695409286553	6.00400625704109	-3.79137344804525
C	4.23869704605637	4.70584140947207	-5.28190295666455
O	-3.60654168850850	-2.82989034707778	-0.33917701299492
O	-3.10780765051722	-1.33938126514247	-2.33096825376366

H	-7.82452784778925	4.79106075793697	-0.28499906387167
H	-3.86863617091322	5.49749465467446	1.07011030339584
C	-3.15785426322307	7.21156596314932	0.02351582855538
C	-1.99058627429886	6.83021657267971	-2.04732327379573
C	-1.60940485453936	4.53173113677506	-2.83661876989129
C	-1.09533218191036	2.24867840926613	-3.67562245760236
S	2.45925726638079	-1.61275853323276	1.31587103517644
C	2.79987496043818	-2.13078716490952	-3.15601138648584
C	5.17310360085544	-1.69465143196868	-3.56460733679335
C	6.19056085643946	0.46646687525758	-3.03593166997499
C	4.54568163367278	3.37363119580916	-5.62499944132509
H	4.54570785419084	1.33043719351269	-5.01937887596143
H	4.44037204869031	5.50595274569170	-5.98640032255732
H	-3.53022589391336	7.90381638727209	0.77174672176770
C	-2.47936473923908	7.70463131090207	-1.10914441307703
H	-1.45642793772938	7.19727034337765	-2.91905120318568
H	-1.09589300041025	4.93216234762101	-3.70585973845150
C	-1.76104473449803	1.11913756560996	-4.15707928402474
C	0.16911168501610	2.53612184034942	-4.20414983508199
O	2.41174918661288	-0.47626046065887	2.25728851840044
O	2.19972697612265	-2.96333421491388	1.86122055168840
C	4.04652407962769	-2.54155336066552	-3.58944711445063
C	1.66267996418970	-3.07671960399705	-3.14139102924143
C	6.45696689103761	-2.17178772838010	-3.92492147521862
C	7.42605791195993	-0.03597155735403	-3.36919430699603
H	6.09706043280337	1.48428990074707	-2.67332623666677
H	4.97331414089625	3.15327334155611	-6.59782988659815
H	-2.33922272016184	8.77338044086232	-1.23261411493655
C	-1.19596627619288	0.32569738683581	-5.14892988341326
H	-2.73605188430661	0.85433422162592	-3.76883534342397
C	0.72377282518813	1.74329412747786	-5.19635107070675
H	0.73282765336406	3.37962787881216	-3.81680140960786
H	4.17400969604882	-3.55506257494485	-3.95455521884467
C	0.34684386283556	-2.69943842869322	-3.45042930731610
C	1.89450132089677	-4.43173822982196	-2.87711817414872
H	6.55403849562324	-3.20241557220794	-4.25306504230128
C	7.56115238216486	-1.36095615849295	-3.83124153121307
H	8.30587277698793	0.59038674707847	-3.26570304446854
F	-6.37858274717796	2.57324686156989	-2.92461694331412
C	0.05150946236807	0.62341556986668	-5.70306764322320
H	-1.75772069484155	-0.53492587045262	-5.49293538296993
H	1.70473211701060	2.00876053189073	-5.57717825585323
C	-0.66366448895882	-3.64569679933039	-3.53854021747473
H	0.10573463152373	-1.66389623295313	-3.65337434826670
C	0.87975561584778	-5.37648908349621	-2.98750510502528
H	2.88703008801515	-4.75795218941756	-2.58157436115705
H	8.54147327363376	-1.74292539496919	-4.09696368826342
C	-7.27870351671220	1.67862059066599	-2.45151820128770
C	0.66093210066089	-0.18056861888762	-6.85356585659686
C	-0.42073918644391	-5.01171554200738	-3.34636501428756

H	-1.66321383022722	-3.29663530273711	-3.77787117372910
H	1.12522646909120	-6.41530683222855	-2.79896104076031
F	-7.64711112751498	0.90525729196318	-3.50281514163126
C	-6.69609259464509	0.84101114087009	-1.34697716151220
F	-8.37083921161595	2.36626765581179	-2.05969020445897
C	-0.14418568403005	-1.44627459205637	-7.17242331506100
C	2.10052503176557	-0.60249269556200	-6.51053312877485
C	0.68426953055170	0.71668898081436	-8.10704840720360
C	-1.54555553415029	-6.02770564938442	-3.56105177792412
C	-7.40745110947678	0.60505917895424	-0.17691108736800
C	-5.45615543996075	0.24665243823578	-1.56087198797821
H	0.34839624073360	-1.99366678831111	-7.98213037799969
H	-0.20368734269025	-2.11366030186974	-6.30612690249450
H	-1.15999710827439	-1.21061670349172	-7.50537014081357
H	2.12458141635143	-1.23557114312460	-5.61915307782144
H	2.53132401184848	-1.16852888175461	-7.34361801914629
H	2.74327931821408	0.26343707713256	-6.32933193707787
H	1.29029109312431	1.61260887334278	-7.94085836012359
H	1.10859936366206	0.16899179356165	-8.95608962300629
H	-0.32824264426979	1.03719492782042	-8.37260442880124
C	-2.63618648939873	-5.83841583790862	-2.49178855036405
C	-1.04309299627174	-7.47437552359230	-3.48629296795266
C	-2.16514263562076	-5.80597739001294	-4.95496822296873
C	-6.85904008487296	-0.22630699158557	0.79513962909141
H	-8.37530942937341	1.06338542562871	-0.02185978049461
C	-4.93466701709811	-0.57793258032103	-0.57676650608243
H	-4.91215985714351	0.39660616249916	-2.48601465301222
H	-3.00336864225015	-4.80792314161298	-2.45822889431447
H	-3.48752651824986	-6.49633333570739	-2.70117594675787
H	-2.24484663589583	-6.09979989783199	-1.50312693524515
H	-1.87709517585020	-8.15832307858973	-3.67160039504655
H	-0.27143711342996	-7.67326404618367	-4.23729768029200
H	-0.63719273838430	-7.71377786423581	-2.49790211152177
H	-2.93525394931315	-6.56129402103300	-5.14561805263761
H	-2.63619864216430	-4.82279444551376	-5.03583951420867
H	-1.40242240431032	-5.88358840089631	-5.73637422134491
C	-5.61588469762839	-0.82296540620760	0.60888734324762
C	-7.62689140006523	-0.55293901083760	2.04722885984988
H	-5.19761702543040	-1.49018343608262	1.35679878419046
F	-8.77782369248571	0.14270679019344	2.14201169817572
F	-6.90337105463574	-0.29017928825294	3.16555883885725
F	-7.94283280105742	-1.86895608498593	2.09678894959463
C	4.59409619537649	-2.85509889460628	0.07256411047677
C	4.15009954658882	-1.68570205223338	0.67492916670379
H	3.92464855031915	-3.70246372758708	-0.03285468815855
C	5.91271136749943	-2.92111444421789	-0.36820072264603
C	4.98507922154129	-0.59023802257793	0.83530552508191
C	6.77014241604112	-1.83545004593044	-0.22310619112405
C	6.41488187964956	-4.21792107425827	-0.93852906377063
C	6.29455601225877	-0.67247841182610	0.37234563615614

H	4.61451752357302	0.30523013993733	1.32043028980688
H	7.79057751459156	-1.89155642875756	-0.57957806792243
F	5.55902899073215	-4.73039985163910	-1.86029568944303
F	6.54276014894046	-5.15971588366969	0.02807204076836
F	7.61884699651763	-4.09259370613336	-1.53289133462221
C	7.20688867023888	0.50234201528896	0.58896078232571
F	8.28771053933488	0.47319329124307	-0.22045912149091
F	6.56821743353534	1.67662732676992	0.37440955833064
F	7.66880149377801	0.53359188180345	1.86435444901620
H	1.00677876084041	-4.22437148185211	2.81353858928001
H	1.05039112201103	-6.81547195117847	1.92219023739514
H	-1.17328842218222	-6.32869940188159	1.09330476441141
H	0.01078442389658	-3.64788740785491	0.14280105917072
C	-8.33071161362526	-3.60337574987172	-1.04048164794616
C	-7.03759248723540	-4.22016761487598	-0.50283270671797
C	-8.04847398754916	-2.70987064258882	-2.25062524167949
C	-7.31840632513659	-3.48410925425113	-3.35033469025410
C	-6.02476804644696	-4.10546163714877	-2.81827411433114
C	-6.29020037346411	-4.98836218780822	-1.59563156810597
H	-9.02054633665098	-4.40608226111504	-1.33832929290390
H	-8.83342158220866	-3.03075858443803	-0.25225313084289
H	-6.38384479849973	-3.42250422249505	-0.12503318401144
H	-7.25355099885668	-4.87666226279119	0.34857244864706
H	-8.97977489461780	-2.27937508426306	-2.63771653242117
H	-7.41919080848358	-1.86721471728885	-1.93129987758688
H	-7.97630434014688	-4.28133873436638	-3.72519261202984
H	-7.10188563028093	-2.82529607003153	-4.19954801252161
H	-5.53027123899456	-4.68729075997425	-3.60602774076205
H	-5.32743248388364	-3.30470712888798	-2.53747905330165
H	-6.88856374094437	-5.85890658278452	-1.90187891491594
H	-5.34165885775190	-5.37253672091643	-1.20147867843814
C	5.57776394335524	5.03956547264986	0.59461041797701
C	5.08046950808636	6.31970830809155	-0.08169317727014
C	5.83479849896611	5.27024575009778	2.08601361611748
C	4.58254453018400	5.81188334931821	2.78113697514084
C	4.09155564738904	7.09695196734902	2.10955453510346
C	3.83373357792272	6.86900385788403	0.61769025391777
H	4.81955843068832	4.25164633298647	0.47789882978517
H	6.48691521163822	4.67648625563375	0.10141857601520
H	5.87610363965062	7.07765115174124	-0.04404029624628
H	4.87468412245537	6.12923119412649	-1.14225874974376
H	6.16317800924600	4.33861542415482	2.56087473345376
H	6.65542883104712	5.99266478347267	2.20474336989525
H	3.78608467345483	5.05619474741189	2.72458570276602
H	4.78040481060159	5.98867683317283	3.84554194630454
H	3.17854307143029	7.45703687313011	2.59880846058788
H	4.85046015223135	7.88327254587568	2.23202634554993
H	3.00883557702869	6.15025146449019	0.51356025898619
H	3.50776550623195	7.80154248798343	0.14021715994188
C	4.00146304171310	3.24921725863837	5.74499433859650

C 3.30614754485683 1.94122867017666 6.13494105556343
C 5.46822461619653 3.01149549661314 5.37994631971140
C 5.59361363082859 1.96894152745251 4.26665563588001
C 4.91540042945999 0.65751931320978 4.66650956007009
C 3.44913066217808 0.88785346382075 5.03381840018179
H 3.49347489581964 3.68616623165201 4.87339079645863
H 3.92130486459188 3.98193381994273 6.55723571435571
H 3.75331015546621 1.55815748823324 7.06352469178949
H 2.24601740203887 2.12760720012021 6.35176899839585
H 5.94167269271859 3.95325689556731 5.07804369615392
H 6.00943241417351 2.65368233693671 6.26747352722233
H 5.11295881728626 2.35846196253481 3.35554759067242
H 6.64695840073766 1.79819644935790 4.01910258290705
H 4.98408704295507 -0.07798996281316 3.85668601560718
H 5.44299100183976 0.22638331698819 5.52949920133785
H 2.91233015330834 1.21558115471741 4.13412632360404
H 2.98823812265405 -0.06009136998190 5.33717687738522
C 6.00745859341403 -3.06867963733392 4.74505826925545
C 4.47728481036799 -3.02292593872001 4.72685948860863
C 6.55074718796987 -3.83438623041879 3.53592196641621
C 5.96225489323727 -5.24613381757523 3.47177192088433
C 4.43309301625785 -5.19737385015807 3.44018583496926
C 3.87802410119272 -4.42914840168965 4.64216318100056
H 6.41901820141605 -2.05289063665717 4.76784435822107
H 6.34267553762507 -3.56905269740197 5.66510896906818
H 4.10276138908639 -2.50066183279088 5.61634668993262
H 4.13826006142223 -2.44133851143930 3.85840083837084
H 6.28168423550130 -3.28951581619405 2.61858738932716
H 7.64613171751879 -3.87586871780865 3.56939864193328
H 6.34724943100255 -5.78158417828493 2.59706120800272
H 6.28834712025479 -5.80903801381140 4.35844330501609
H 4.10615480813107 -4.69217085219117 2.52094971975258
H 4.01511869829571 -6.21091559842261 3.41307676100892
H 2.78592422155814 -4.37503281686169 4.57639375601397
H 4.11530991535861 -4.98149608308435 5.56340418664482

228
Minor0 0 CyH
C 0.18126139203219 -4.44332527703723 -0.85186183441309
C 0.54140134064334 -5.69407607277398 -1.58259795356866
C -0.72674750326627 -6.52687479958938 -1.89426516074479
C -1.80123250405266 -5.73516085066067 -2.62642532943857
O -2.39860767822295 -4.80667727432902 -1.72231536365781
H 1.03872422756958 -5.42891529476699 -2.52023250093638
H -1.15749077270254 -6.91139016398161 -0.96220268303648
H -2.56572030908423 -6.42889022154961 -3.00871738696111
C -0.24866306364128 -4.52413602587702 0.54182344698439
C 0.08400530276015 -5.63092554184971 1.34272734185257

C	-0.92562608478701	-3.45327138671491	1.14269284418145
C	-0.21678103118669	-5.64159908291136	2.69524728609177
H	0.62681087158958	-6.46774459060861	0.92115073496564
C	-1.22354917597873	-3.46247646910362	2.49406323476636
H	-1.25142994121089	-2.61706414224265	0.53956091570585
C	-0.86022665898158	-4.55230766900524	3.27785953284371
H	0.06971923997668	-6.49367490896888	3.30266678647576
H	-1.74046477656068	-2.61469936230137	2.92783758111114
H	-1.07864691531160	-4.55840257293323	4.34127041767412
C	0.34587562625941	-3.22205467373784	-1.48899646918998
H	0.64860945606116	-3.23030101319216	-2.53332613377268
H	-0.33435047904716	-2.41120749181209	-1.22738662742078
H	5.15445771121675	1.98400910375538	-5.89316938738438
C	4.49733082855838	2.04002005760359	-5.01853987980182
C	3.26512525621705	2.90187946237158	-5.35218722063215
H	5.07950495263434	2.45668531588333	-4.19188211166073
H	4.19809746442361	1.02380409306783	-4.74517045669238
C	2.34614515236404	3.02072475157975	-4.13448280410492
C	2.55440904276119	2.26360495003340	-6.55172806383355
C	3.72909626189872	4.31777741691518	-5.74660590335845
C	4.08747063303288	-1.88560422441004	-3.29069591025593
C	0.97238319897717	2.77278551803651	-4.19342977298444
C	2.86339607828872	3.42809072721689	-2.89780317875495
H	1.71478690494394	2.87255431308489	-6.90136317554839
H	3.25951866532762	2.16724939549717	-7.38338001741314
H	2.18439362442594	1.26219048482988	-6.30645350217186
H	2.87150324927442	4.95268290540641	-5.99120319804814
H	4.28072724574259	4.79478191323508	-4.93070631389434
H	4.38670732720287	4.27045003046568	-6.62186069421593
C	3.47892306536413	-0.73538558517559	-2.77614244770949
C	3.49645785663767	-2.62111398824140	-4.31317904432626
H	5.03665847389545	-2.21842039622089	-2.88147440910777
C	0.15513702015140	2.91742348458067	-3.07757576163185
H	0.51312996727732	2.44635046773575	-5.11875053822645
C	2.05236260194740	3.58271842639171	-1.78493325916544
H	3.92611281468822	3.62395066688434	-2.79128944487576
C	4.18370155973515	0.06525665521688	-1.75450799396435
C	2.23335865375733	-0.36992719682619	-3.30635981901805
C	2.27089504551408	-2.24070112670681	-4.86580663369187
H	4.01283877956573	-3.50205790301400	-4.67604876722187
C	0.67771901410468	3.32886352010600	-1.84853360455314
H	-0.90433318499912	2.71912975871134	-3.17660784894773
H	2.49480545316637	3.88497015515901	-0.84275610167851
C	3.51528859302081	0.71422983171592	-0.68621575320134
C	5.55646398653703	0.20957172195720	-1.79718455292780
C	1.65330642165078	-1.10481767506159	-4.32769491422112
H	1.72423317168718	0.51575992883353	-2.95287751606680
C	1.59436220333205	-2.99472976028438	-6.01010864868348
C	-0.18724637025692	3.59766070147934	-0.68111197616709
O	2.12387788282436	0.64031937671788	-0.65617925311156

C	4.14726231167222	1.42794897077727	0.31689484628926
H	6.10846478334279	-0.21553780075632	-2.62907278395982
C	6.27579546006385	0.87270938591473	-0.78235271977641
H	0.68814211213105	-0.77634662225675	-4.69763819416950
C	2.44144400064101	-4.16707843543697	-6.51706064877925
C	1.35943634208299	-2.02256601025882	-7.18276725672458
C	0.23953441628925	-3.54385155585181	-5.52644210994106
C	-1.32752945988026	2.81086010789874	-0.37017703611917
C	0.03313193502339	4.70193757824498	0.11744916086796
P	1.47389886098011	-0.35744622065196	0.43172638723906
C	5.57603098555203	1.46156554754993	0.31725348271745
C	3.33950158641119	2.12838580734719	1.34608320028276
C	7.69070215294439	0.93240303079042	-0.81102678841096
H	2.61773396534241	-4.90992184203807	-5.73179088307153
H	1.91613316860922	-4.67029002324389	-7.33480088524366
H	3.41047163692779	-3.83036643282181	-6.90024889935469
H	0.91088469628225	-2.55570574799944	-8.02801487506388
H	0.68329223091996	-1.21047416121047	-6.90222766339051
H	2.30488562718735	-1.58113546049285	-7.51485111369831
H	0.39292468571109	-4.28571649571968	-4.73533275232956
H	-0.40746909415623	-2.75564753349647	-5.12970501663687
H	-0.28751750970540	-4.03463390886264	-6.35222708278338
O	-1.50856302287896	1.62300105563721	-1.07945752973892
C	-2.28856289621399	3.16582785802394	0.55411999979085
H	0.88218525566280	5.34653172935547	-0.09019862274683
C	-0.82533626558886	5.04023176491969	1.18451859474862
O	2.20661213243116	0.07871760394873	1.79975414897754
N	-0.05397923296263	-0.08028356255391	0.45529613562968
N	2.00156097336134	-1.88044042993492	0.16064734745360
C	6.33092740724701	2.02497065835720	1.37293037653325
C	3.48610564067424	3.53070292008944	1.59363363508266
C	2.36249009365026	1.44634051436573	2.04941839581249
H	8.21127219927034	0.48973723600990	-1.65507055569894
C	8.39266271468558	1.51028878202572	0.21787055137865
P	-1.41228291863369	0.21402010848346	-0.28276192498475
C	-3.57265023872187	2.42841584946975	0.64353123346380
C	-2.01823739199539	4.28424767258898	1.40365571625711
C	-0.53977823514424	6.13851680363895	2.03124135151051
S	2.76855925613686	-2.86431097255596	1.26861503755408
H	1.28811044813094	-2.62761308113274	-0.73244655701235
C	7.70469734908906	2.04775798431922	1.32422309064591
H	5.81338865507501	2.43280283272639	2.23294636552982
C	4.35563158350319	4.36243025001407	0.84532403678557
C	2.67652522164121	4.13764961120163	2.60354788140168
C	1.50571765125526	2.03307093528218	3.01239720389458
H	9.47704232027912	1.53942646198612	0.18913295147327
O	-2.40937115598449	0.37828674728466	0.98801141885589
N	-1.92053066028869	-0.92290962318592	-1.27042765636133
C	-3.61369331932229	1.06542092537922	0.88113649455327
C	-4.80786204723886	3.13394667812302	0.48347026963638

C	-2.84957504693262	4.63514962846439	2.49439149885261
H	0.36085547569908	6.71461889367718	1.84144864073705
C	-1.36913645605208	6.45680297369757	3.07795615654404
O	2.90316190999451	-4.15610532573438	0.57982135549402
O	2.14928792103546	-2.79037659449891	2.59712606764558
H	8.26308658671389	2.47427659066920	2.15091506272878
H	4.94246738770576	3.92984927719701	0.04382717107727
C	4.45729748742991	5.70652538662329	1.11401427046907
C	2.83046251811531	5.51920504825990	2.87759910866952
C	1.71268332702664	3.37025751200619	3.28667043007241
C	0.40982994688524	1.29659431478343	3.67630048181776
S	-2.39749722315370	-0.87200524695195	-2.81031266018964
C	-4.81923787008657	0.36200732159735	1.16406322288063
C	-6.03086359707536	2.44574699215914	0.74693650480715
C	-4.87376217688497	4.47564652596095	0.03821539383900
C	-2.52784713655764	5.68947869386042	3.31573246693413
H	-3.74006938150605	4.04808067418967	2.68747797708665
H	-1.13578501602881	7.29594701053057	3.72540686909665
H	5.12528171617281	6.32281495959036	0.52125697790233
C	3.70340496166002	6.29047305953913	2.15231759477041
H	2.22591613499878	5.95722743007135	3.66643322634879
H	1.10137194103854	3.85515008966286	4.04171356712694
C	0.54463403758724	-0.00008640032627	4.18751712992383
C	-0.82232298457541	1.93465571903609	3.83791013878319
O	-1.46728031700295	-0.14519619696840	-3.69317236233547
O	-2.76158892584777	-2.25968438793560	-3.17656029374565
C	-5.99307916468228	1.08757796835778	1.11685105123839
C	-4.87680719964039	-1.07137464519289	1.52190451866372
C	-7.26091257186550	3.12960797561112	0.58937350897311
C	-6.08403958318352	5.10763375927924	-0.11844120617462
H	-3.95695927301171	4.99935497564192	-0.20413388880388
H	-3.17144241868548	5.93205036370470	4.15503786402307
H	3.80783520595089	7.34908672530363	2.36692026289368
C	-0.50611840618004	-0.60354101824619	4.86350745247014
H	1.47516550485417	-0.54267329072569	4.07353530261968
C	-1.87389591016783	1.31698579237777	4.50200098347053
H	-0.97056607149275	2.92401696047810	3.41878749803646
H	-6.93088433117767	0.57625514395122	1.31099707292607
C	-5.71062993133693	-1.49585077564677	2.56627246762910
C	-4.19275870265690	-2.05296235869170	0.80537210398112
H	-8.18210943665642	2.59165657040265	0.79223657256863
C	-7.28933407690258	4.43579335125627	0.16812817139072
H	-6.11146180113577	6.12932205627543	-0.48259723386803
F	6.38271530261373	-1.19089362098617	5.15105625000547
C	-1.73798727913546	0.03865367903578	5.04806540676795
H	-0.34559414711240	-1.59980183752376	5.26438281205972
H	-2.80996808068368	1.85620843254276	4.58833847976378
C	-5.82284813700041	-2.84038544190905	2.88769486520606
H	-6.25418974836097	-0.75995410136124	3.15163075871671
C	-4.31585588096277	-3.39999283416606	1.12524527686215

H	-3.55810159134793	-1.77527148259964	-0.02477541429408
H	-8.23703862630397	4.94864842285474	0.04080224557975
C	6.42211026617879	-0.37227681499582	4.07118684083757
C	-2.84232019187247	-0.62686277209320	5.87229701417682
C	-5.11730444113020	-3.82610454551714	2.18431020077706
H	-6.46284207868191	-3.12046718273727	3.71896783635751
H	-3.75642225140432	-4.11241307142398	0.53020494460787
F	7.65482751237943	0.17652547740555	4.03354592294668
C	6.08617480060939	-1.11837261880007	2.80913913928924
F	5.53669880197451	0.62521440211478	4.30692304954758
C	-3.13251595503729	-2.04331218068414	5.34827003063865
C	-4.15319245466953	0.16810792386732	5.83033340329806
C	-2.36623332410684	-0.71482269977729	7.33556216270006
C	-5.22169771203977	-5.29411495899650	2.59649725114323
C	7.07885763265774	-1.46738236622998	1.90014064689638
C	4.76404727636574	-1.49783242225867	2.60301579920143
H	-2.24832483509806	-2.68516693969400	5.39720006253737
H	-3.91310406276259	-2.51387142601674	5.95636798255255
H	-3.48738212866816	-2.01360823363952	4.31390278408733
H	-4.04477332251305	1.15862915472391	6.28364032350613
H	-4.51057613630559	0.28980154112793	4.80228476636549
H	-4.92297690926790	-0.37017326316819	6.39241038414915
H	-2.14599425529814	0.28160395631482	7.73185464410793
H	-3.14209538871489	-1.17279991431835	7.95946048273706
H	-1.45849813364613	-1.31964646292408	7.42027223726514
C	-4.77393903166374	-5.43643898337157	4.06373916117033
C	-4.33094571642977	-6.19798752204609	1.73458502941477
C	-6.68166667553958	-5.76255468019356	2.45583165522177
C	6.73281037214431	-2.19041244810333	0.76359167395748
H	8.10588842704474	-1.17108327300495	2.06942400754662
C	4.44633524885648	-2.21723977128491	1.46023081503790
H	3.99228469463616	-1.25979284748787	3.32513721663060
H	-5.40549334266824	-4.85234348465982	4.73934094658370
H	-4.82712260834691	-6.48590112972935	4.37380627004070
H	-3.74116777669945	-5.09237104544852	4.18296889562585
H	-4.42509123747488	-7.23319266654257	2.07816252207414
H	-4.62027319740697	-6.16686382178670	0.67941016974341
H	-3.27596658230884	-5.91302206473243	1.80937913545439
H	-7.35687203573975	-5.17601040413582	3.08599872484772
H	-7.01794480320035	-5.66552846432615	1.41872993520640
H	-6.77185933505429	-6.81377769280849	2.75149182168214
C	5.41369881000464	-2.56987424556028	0.52966555671657
C	7.78904810721829	-2.64310615642487	-0.20589795818369
H	5.14189617400211	-3.15526070651983	-0.34222279671629
F	7.43588578758726	-2.38445329178422	-1.49159732474462
F	7.98504720718672	-3.98160341578026	-0.12448229675525
F	8.98087869706825	-2.05076276461663	0.01029452173002
C	-5.10040536441763	-0.51047171608702	-2.32291923795146
C	-3.93496918192822	0.07878078774797	-2.79794128641955
H	-5.10350219291453	-1.54833087345955	-2.00842097250306

C	-6.25340064414908	0.26230603387781	-2.24890786294505
C	-3.90941793494703	1.39829386681124	-3.21874961111084
C	-6.25129138422524	1.59692889612559	-2.64957597240954
C	-7.52962384868739	-0.37291359652970	-1.76892700059796
C	-5.07598238688793	2.15375861895318	-3.13670295790354
H	-2.99016573736842	1.82319620529441	-3.60347277099510
H	-7.15001767784103	2.19557055427449	-2.56802605489956
F	-8.18821717318928	-0.98357102457053	-2.78545349502383
F	-8.38148729860987	0.53870856945573	-1.24113332195781
F	-7.30417594667830	-1.31063860710996	-0.82492090392219
C	-5.02822223431914	3.57762906008928	-3.61615379355142
F	-3.97242938601151	4.24197530821110	-3.08536879492222
F	-4.88233401000941	3.63473620823132	-4.96406796978429
F	-6.14383214570220	4.26727972803876	-3.30580691993612
H	-2.54057546335919	-3.96434458563133	-2.18566803534391
H	-1.36822476670003	-5.21049817882159	-3.49037840862655
H	-0.42865497204223	-7.38958283162974	-2.50168864521294
H	1.24212741248715	-6.30446872826364	-1.00367931466510

246

Minor0 1 CyH

C	0.55624912157660	3.69608836759815	2.58262558195502
C	1.02890788013748	4.43409620801228	3.79251210544798
C	-0.15313719376460	5.12672157810874	4.51485414639602
C	-1.29350730455674	4.17451402266599	4.84479856091819
O	-1.97668017152086	3.82685486857738	3.64091578664226
H	1.50080942991658	3.72375316052950	4.47824923556571
H	-0.55296995805846	5.93624000608307	3.89322995380518
H	-1.99091782735780	4.66731840693900	5.53922461189717
C	0.17513161792521	4.44510061986815	1.38785545313912
C	0.50802017214922	5.80391056768927	1.23884841607252
C	-0.43349024079586	3.79525135224746	0.30420596604783
C	0.27051661955567	6.46506131174496	0.04456682604208
H	0.99383674026931	6.34044687891639	2.04415234961538
C	-0.66200530167488	4.45178722909941	-0.89185330955205
H	-0.74407183145230	2.76490506923352	0.40039367263042
C	-0.30276629185263	5.78761761301789	-1.02982934504282
H	0.55119204674235	7.50822664882671	-0.05715162379848
H	-1.11610898225191	3.91044366687151	-1.71284364324977
H	-0.46625898057672	6.30335102895479	-1.97107454194774
C	0.59131163028271	2.30853766691897	2.59444039033290
H	0.88319559225452	1.81992525896106	3.52118743595140
H	-0.15967436333594	1.76751356757112	2.01821470416304
H	4.77127358964151	-4.81576018877370	3.77676211231241
C	4.12039670989982	-4.39644497897205	3.00177025678907
C	2.81001935441360	-5.20276804453229	2.92856226004696
H	4.66990659063537	-4.41619851005719	2.05647709516890

H	3.91355959323837	-3.35207916945850	3.25262530857783
C	1.91999568295684	-4.66064145200973	1.80891765880050
C	2.13042490460704	-5.12078445589515	4.30009172684473
C	3.13492882644415	-6.67770191203079	2.62323950946527
C	3.87633235098883	0.17102032108615	3.69130387996604
C	0.59049374411593	-4.27935305050177	2.00731330331653
C	2.42223147946194	-4.54978719905511	0.50542625821103
H	1.22548983952754	-5.73464623710079	4.34567575885023
H	2.81739925332435	-5.48750683986592	5.06931226560070
H	1.86533947425819	-4.08825317666501	4.55238497412062
H	2.21709737064777	-7.26963850705784	2.54786876375350
H	3.67969426655550	-6.77727289149708	1.67948069346114
H	3.75574053326365	-7.10059957633305	3.42095032330904
C	3.34181314021088	-0.66996061417883	2.70983344403023
C	3.19355489158038	0.41605028494836	4.87879857871490
H	4.83413987652304	0.65415224258101	3.52058582179453
C	-0.19766217738012	-3.80250124539545	0.96488889754104
H	0.14481693339140	-4.33182644386816	2.99438115413383
C	1.64036866183808	-4.08279978100939	-0.53795127867718
H	3.45003587149165	-4.82845048785204	0.29387234844496
C	4.11691014684157	-0.96823940657506	1.48856067425866
C	2.08906269902016	-1.24501152153047	2.95872100734483
C	1.95442213863269	-0.17616487957705	5.14162860052387
H	3.64542233971303	1.08617460473260	5.60099110825572
C	0.31110257880076	-3.69494071570755	-0.33308295080914
H	-1.22085042041956	-3.52048738977866	1.17620095453362
H	2.07561890826602	-3.99810542758576	-1.52636075153397
C	3.51618163372837	-1.06242201044493	0.20834247897496
C	5.47938183549208	-1.17788578331532	1.55012750176550
C	1.42373171278838	-1.01110296690930	4.15129959684193
H	1.63620812142459	-1.90601822056867	2.23211178500349
C	1.20178757250891	0.01816993522386	6.45842801035660
C	-0.52379920952223	-3.28423508727514	-1.48053867140002
O	2.13275559175868	-0.89300920163850	0.13264278824166
C	4.20849301684587	-1.31108183941294	-0.96431597261976
H	5.97288302379581	-1.16958119466440	2.51679508601333
C	6.25857658795508	-1.39400398485630	0.39528705000837
H	0.45754977154640	-1.48269194635913	4.28849716703190
C	1.73283284507044	1.21545271001579	7.25723432445501
C	1.37877543607742	-1.26051421182914	7.30072922126556
C	-0.29624122275663	0.24447357603990	6.19142204934209
C	-1.55838046508622	-2.31739863869281	-1.37502269845306
C	-0.37574676928183	-3.88725628321299	-2.71374241929682
P	1.61847432349582	0.52471107024380	-0.44343486578727
C	5.63118817697704	-1.42726676070807	-0.88990169128007
C	3.46901327138958	-1.43638973975378	-2.24534176723836
C	7.66400412421533	-1.54613694613168	0.48092307936763
H	1.66703874386221	2.14246017598662	6.67580897067177
H	1.13281057038039	1.34329342852672	8.16330956107746
H	2.77204294178934	1.07587724273072	7.57028362795294

H	0.83717667116180	-1.17002208487477	8.24933615013538
H	0.99140093285309	-2.13436257454877	6.76758350176785
H	2.43679083040442	-1.43808552457234	7.52013753676188
H	-0.45401631337518	1.09432735712217	5.52109767168917
H	-0.77587171015191	-0.61980244273148	5.72735490244452
H	-0.81790713110977	0.44548272458894	7.13331900166451
O	-1.65977642176289	-1.60061857720864	-0.18349923315794
C	-2.49559924960656	-2.06873898006038	-2.35605152840001
H	0.39029191389951	-4.64498552130361	-2.84735398856363
C	-1.19921389368983	-3.56631415908485	-3.81279276502301
O	2.45436531798773	0.67766606513274	-1.81267281685462
N	0.08924015344669	0.40257956514786	-0.69531813017878
N	2.18777829745015	1.73261324298102	0.50067677219963
C	6.45194537950535	-1.54116834386676	-2.03708069890961
C	3.57832039709109	-2.59756769358500	-3.07488333194193
C	2.58015039246018	-0.44933310618282	-2.63122077326925
H	8.12839584911565	-1.52991967038204	1.46242551225635
C	8.42867048190559	-1.68430219444012	-0.65125691627501
P	-1.34673549533062	-0.01099408845588	-0.19824460466977
C	-3.67693048235737	-1.21585872259513	-2.08057576767340
C	-2.29881616433626	-2.66964578448202	-3.63838099896508
C	-0.96151010487000	-4.12747199724152	-5.09125928979482
S	3.09458399751453	3.03552222934487	-0.01422350660067
H	1.50094521587646	2.03640154799992	1.63266595076583
C	7.81596954297550	-1.66703112116865	-1.92023397046356
H	5.99379008710562	-1.51910166989272	-3.01848315754759
C	4.35311647581441	-3.72991785423352	-2.72166659438065
C	2.81302893618948	-2.65345186927122	-4.28188700612217
C	1.78006767142655	-0.49401408431216	-3.79851344429236
H	9.50571315582436	-1.79031765080054	-0.57189752949929
O	-2.26618772868063	0.58274216573827	-1.39912832113450
N	-1.79220187092485	0.56811984247088	1.21430924478374
C	-3.54618337793072	0.08937526688311	-1.63626817788449
C	-4.99110822364753	-1.74702795118485	-2.27736940769093
C	-3.09695674486623	-2.35389083544759	-4.76367345692014
H	-0.12856682699757	-4.81556378730813	-5.20456055436656
C	-1.75250458510561	-3.79789584832179	-6.16398278670989
O	3.23355921773556	3.87821825247947	1.18254611207826
O	2.58508301674930	3.60141447475737	-1.26870658916653
H	8.42573085096387	-1.74262292383333	-2.81443920433594
H	4.90128699103837	-3.72458361019145	-1.78731627801247
C	4.40359305571316	-4.83666873327637	-3.53493326485437
C	2.90796164882951	-3.79973403998300	-5.10980174044552
C	1.94396670076607	-1.59533278410179	-4.61431977818270
C	0.75747204320920	0.52629545689297	-4.10919858185923
S	-2.40238685760954	-0.17109214203959	2.51240444342374
C	-4.64622979586445	0.98906040268895	-1.54196831956060
C	-6.10991884460153	-0.86687399587333	-2.17656192841979
C	-5.23494631724083	-3.11782277080554	-2.53235161987908
C	-2.82476965061146	-2.89782041533969	-5.99653696232132

H	-3.91935292108828	-1.65736454432556	-4.64881117767402
H	-1.55480542776214	-4.22556343146282	-7.14150154055868
H	4.99767696627583	-5.69441190552931	-3.23694169552516
C	3.68685755202594	-4.87000122491866	-4.74866721687432
H	2.33698304422296	-3.81583926484231	-6.03380159065418
H	1.36727607384085	-1.66346183378419	-5.53180213165034
C	0.95822370309168	1.90498962512065	-3.96973074409503
C	-0.48702295577545	0.09448962578182	-4.57300623006628
O	-1.65046232436977	-1.37905457906438	2.90379768124414
O	-2.60852503286253	0.88114555210024	3.53266667215235
C	-5.89579301915630	0.48439689524947	-1.84587963780287
C	-4.53624603893988	2.41360679363523	-1.15969060278056
C	-7.41688020600452	-1.37768462604577	-2.36735342780953
C	-6.51572261795936	-3.58495872558090	-2.70509130980111
H	-4.40032579570586	-3.80748733615395	-2.57179833342274
H	-3.43895306640747	-2.62886839353873	-6.84970731896133
H	3.74617723649849	-5.74507767904129	-5.38748904091462
C	-0.04920027074125	2.80019644735292	-4.30214374195221
H	1.90311628901165	2.29084844808605	-3.60671777093632
C	-1.49636051307674	0.99522840949732	-4.88215715253278
H	-0.68181463220486	-0.96789445835484	-4.66305937313054
H	-6.75683384430023	1.14058168793170	-1.76432386403861
C	-5.27349047651950	3.37632334887996	-1.86467974468133
C	-3.80544486941654	2.84561131508143	-0.05336494222643
H	-8.25728786487250	-0.69460915994802	-2.28567006575366
C	-7.61766722976691	-2.70944307881020	-2.63236466612709
H	-6.68097802555357	-4.64302597968137	-2.87941426651484
F	6.91618402411878	2.96361684898961	-4.04479421022967
C	-1.30218498721658	2.37296512634733	-4.76233898022918
H	0.15954628872843	3.86015369650955	-4.19772786716673
H	-2.44829956076849	0.59647815727287	-5.21189568249757
C	-5.26374899792138	4.70708522772106	-1.47708965065544
H	-5.83764690406183	3.07990754173310	-2.74408369948144
C	-3.81002729608183	4.17938573202213	0.33921051768294
H	-3.23213037036548	2.13758843339923	0.52918308390226
H	-8.62288700493592	-3.09430016748989	-2.76956931076197
C	6.85787401217048	1.75840006772802	-3.42654758880542
C	-2.37124809118035	3.38723520881402	-5.17477427044024
C	-4.53019968546792	5.14384923408767	-0.36558868007561
H	-5.83401583843692	5.42036564743014	-2.06431475823589
H	-3.22766656796650	4.45012649784635	1.21228757654206
F	8.07016345723043	1.18131006234976	-3.56639166817389
C	6.45327115991527	1.90656060900497	-1.98557297548532
F	5.96603653726137	1.00950777115438	-4.11819043582415
C	-2.53831850147606	4.47633762261589	-4.10211062438834
C	-3.73866992550280	2.72360771451060	-5.38636745741462
C	-1.92701292029374	4.04333593140525	-6.49690487058527
C	-4.52495001918469	6.62228086170456	0.02097215201530
C	7.38878872088683	1.77837728417737	-0.96482810041770
C	5.13165155361765	2.23764593903542	-1.70616053469835

H	-1.61627786177538	5.04359512722633	-3.94656801846163
H	-3.31152755374472	5.18787235160503	-4.41185804939446
H	-2.85021661390437	4.04128856649997	-3.14879587576935
H	-3.72660255785115	2.01906540474369	-6.22386569275092
H	-4.06450708503640	2.19119831251952	-4.48569675278748
H	-4.48520357103337	3.49128405886948	-5.61392152813435
H	-1.79679450994186	3.28964737163425	-7.28011758636436
H	-2.67834861617447	4.76714053472794	-6.83268165075126
H	-0.97551388465763	4.56970057180117	-6.37370715601654
C	-3.97222804731785	7.44710504241054	-1.15710903363807
C	-3.64873674988870	6.89340920446136	1.25047309367782
C	-5.96320234994222	7.07423784404299	0.33558679852744
C	6.98396843531817	1.96715381967867	0.35199077437390
H	8.41627978679907	1.52530395890669	-1.19131575426575
C	4.75414919237363	2.41758662176385	-0.38312734939524
H	4.40839079604129	2.37702886146146	-2.50061247846030
H	-4.58612108813701	7.33270968348214	-2.05526820189036
H	-3.95069334740296	8.51091268093646	-0.89559803409260
H	-2.95288526417469	7.12891304881014	-1.39855065418378
H	-3.67297210217508	7.96283500304717	1.48354277125281
H	-4.00516470956179	6.34911548687839	2.13075780083328
H	-2.60564288111175	6.61191895667980	1.07199263730670
H	-6.62548134299893	6.94791169180190	-0.52592692653597
H	-6.37579811618323	6.49347145538687	1.16659751973321
H	-5.97279518747340	8.13339920756580	0.61572012386941
C	5.66338592119765	2.28523768506408	0.65654386612394
C	7.97850092908641	1.90008522126187	1.47749901583099
H	5.34973779741955	2.46393915211059	1.67929545951692
F	7.53124565278742	1.12252148838839	2.49741237807527
F	8.20410524498015	3.12825827719715	2.00391761142213
F	9.17087463845443	1.40617655166639	1.08732054905923
C	-5.08483936410447	0.18733185664974	1.92588628839143
C	-4.05121363511766	-0.73615403810715	2.02540998034860
H	-4.91450564681105	1.23205286278926	2.16148018022133
C	-6.33370024864734	-0.25499902942506	1.50424648441074
C	-4.24750943446551	-2.07804248923581	1.73775810001515
C	-6.55917948743831	-1.59848600794709	1.21459440366108
C	-7.46360027091472	0.73214087526323	1.39612740828677
C	-5.51174750674298	-2.50249091812271	1.33841452725556
H	-3.42730303061075	-2.77863754256518	1.83401835050847
H	-7.53344937733161	-1.93138782083369	0.87870460886555
F	-8.09498603730790	0.89573718027853	2.58492170201134
F	-8.40120881784623	0.32724498180565	0.50501985230656
F	-7.03332345357811	1.94980406189345	1.00507622889739
C	-5.73643971030666	-3.97113254030177	1.10734014868715
F	-4.69665474451272	-4.54124456371209	0.45556144022362
F	-5.86721055690086	-4.63280083388735	2.28641883060383
F	-6.85443387095531	-4.21706036486879	0.39420173547072
H	-2.18318111175129	2.87837775801937	3.64591895694593
H	-0.90294569540137	3.27929082898040	5.35011868742802

H	0.22664092880343	5.57730490348411	5.43956882608112
H	1.78459426871747	5.17966579594602	3.52561524895270
C	-2.93700003402656	-4.53253636071892	4.24418453523120
C	-1.87511048143638	-5.17849922439896	5.13692432489109
C	-3.93829376985204	-3.72710197688348	5.07717372231707
C	-3.22650364360128	-2.67480424015298	5.93168496465911
C	-2.14501923683056	-3.31093694575794	6.80976081473774
C	-1.15173530198598	-4.11385956450056	5.96498380713783
H	-2.43098768329965	-3.85270464992573	3.54410727621490
H	-3.45604205994235	-5.29175279478652	3.64767303627507
H	-2.35547580134047	-5.89990298046841	5.81308254962840
H	-1.16154401345349	-5.74565393924204	4.52695573109356
H	-4.67643803062782	-3.24531496049037	4.42511659588900
H	-4.49539437482393	-4.41414249405698	5.73010636212725
H	-2.76065560992199	-1.93464819944035	5.26772642578214
H	-3.95115052950304	-2.13534013534400	6.55297650951228
H	-1.61952482929487	-2.53812703822484	7.38414533218163
H	-2.61675601460834	-3.98235812996354	7.54158041757870
H	-0.63195243220829	-3.42774359678645	5.27928529195525
H	-0.38441699759139	-4.57245994865998	6.60091877584319

264

Minor0 2 CyH

C	0.76323743001555	4.10940536176378	-1.16654411227313
C	1.29389141110648	5.49709530784328	-1.01035230228617
C	0.19940523818865	6.54274085354128	-1.33465750114933
C	-1.07065259054945	6.35335668591808	-0.51697142232395
O	-1.75755194774400	5.18789715757230	-0.97174161365965
H	1.62901353948216	5.63469822857782	0.02250784797295
H	-0.06424951577097	6.49168141343841	-2.39761810092102
H	-1.71702949665665	7.23557528327368	-0.63977420534993
C	0.51250408928654	3.58241164203460	-2.50663273572368
C	1.10111358149742	4.16643701417519	-3.64313909234477
C	-0.23482196512548	2.40884268743524	-2.68261540547253
C	0.97181718515039	3.57692803638863	-4.89045370579376
H	1.70655486879738	5.05941366476768	-3.54891389597819
C	-0.36031622762161	1.81744368996749	-3.92719926840027
H	-0.74392061048040	1.96483025705221	-1.83857895915483
C	0.25081637615586	2.39364995431035	-5.03493501744675
H	1.45524807031375	4.02884454228970	-5.75017009869773
H	-0.93393680131222	0.90345092020618	-4.02295752264859
H	0.16704178598026	1.92514956061656	-6.01067953204487
C	0.61347414992523	3.32002470523463	-0.03633662669794
H	0.81125620372065	3.77416801239761	0.93128679568281
H	-0.18274862246903	2.57551502323506	-0.02948955990950
H	3.92427579114692	-0.22150113409108	6.74938401769493
C	3.33038739565956	-0.58018464584770	5.90160861585811

C	1.91905675838669	-0.96717341298020	6.38028714275427
H	3.86206888940264	-1.42642924950195	5.45710429248754
H	3.27850109424822	0.21782596813430	5.15537241157445
C	1.08487874069989	-1.47995826848891	5.20516116750551
C	1.29003652007638	0.26434771484024	7.04212144706571
C	2.02635871880186	-2.09106187550718	7.42925780974947
C	3.65165615965700	2.73134873477500	2.62173626306617
C	-0.18457801632015	-0.98358400540852	4.89727155328691
C	1.57295635549450	-2.51350772871925	4.39435908473045
H	0.30896925863013	0.03938061951947	7.47157114787506
H	1.93453839141065	0.60993589008155	7.85657574512337
H	1.17847714390887	1.08890350704394	6.32974008049343
H	1.03326386434951	-2.38654604513618	7.78268431993145
H	2.51525556264897	-2.97752007669938	7.01388324034203
H	2.61278097549731	-1.74941147085092	8.28949859286147
C	3.01657762050311	1.48656251323933	2.68038083512290
C	3.01510526160360	3.88645315251455	3.06586780156751
H	4.65478684744687	2.80263501779101	2.21129913749633
C	-0.92814834770695	-1.48348963035370	3.83324293369431
H	-0.61601685039690	-0.17802580542269	5.48023990847437
C	0.83486358077693	-3.02035314148891	3.33777857554152
H	2.55571525968884	-2.93439874288931	4.58389732123586
C	3.74281386146766	0.26738837017814	2.27209110373572
C	1.70929106266114	1.44719749512421	3.18345441080640
C	1.72264849664792	3.84661288448555	3.59775185425088
H	3.54761083516552	4.82738243915577	2.98949946473023
C	-0.43435361012256	-2.51601482141147	3.03006244869301
H	-1.90630205828654	-1.06208984902018	3.64155656016209
H	1.26085241223402	-3.80708114027590	2.72627122360508
C	3.12342664888885	-0.78621147911200	1.55476620767753
C	5.07850349203320	0.10366546053550	2.58103961433087
C	1.08922518314548	2.59864756459753	3.64052966335192
H	1.17558407918872	0.50858861379443	3.25154417874030
C	1.01191740111026	5.07743451890102	4.16092699441972
C	-1.23238550836493	-3.13765166168427	1.95356110334439
O	1.76392960715750	-0.65823257878274	1.26096815524036
C	3.77660138981342	-1.92693772512124	1.12274693230858
H	5.58082382331978	0.86092234819594	3.17483886784973
C	5.81986787936528	-1.01242587697203	2.14263536044832
H	0.07852486243034	2.50683138167212	4.02243596475512
C	1.73810197001782	6.38073344654979	3.80672790536244
C	0.96362893313750	4.94128190140745	5.69552206555911
C	-0.42426851364813	5.15864911293058	3.61517645357010
C	-2.13576605905841	-2.40688018170524	1.13778903151406
C	-1.17595927915822	-4.49957722685322	1.73349063709828
P	1.40618273374638	-0.25782230142009	-0.26153137184198
C	5.17990663800867	-2.03162196511337	1.36942087957594
C	3.01601949823617	-2.98551390910134	0.41382047404592
C	7.20308771155313	-1.12749219474226	2.42456311110965
H	1.82910638263926	6.50682084601859	2.72182938162104

H	1.17077190273545	7.23133138570286	4.19682316317064
H	2.73963297605171	6.42480450674914	4.24584411759836
H	0.44497387983819	5.80007262011684	6.13646998680477
H	0.43152464467548	4.03253538095818	5.99217288000159
H	1.97449908671026	4.89437193484558	6.11389592861376
H	-0.42626241086479	5.22020482259778	2.52267688655621
H	-1.02638573880614	4.28857020040630	3.88600313638354
H	-0.92595588986303	6.04814584133514	4.01121009452283
O	-2.13925066007861	-1.01704330339794	1.25500029400295
C	-3.03485510312786	-2.97981806339162	0.26242374568036
H	-0.51658053483815	-5.10997990556503	2.34275359250623
C	-1.95871895590506	-5.13660875399266	0.74894742238203
O	2.24293699745849	-1.33426767315166	-1.12246559699591
N	-0.12727187954921	-0.42456504486309	-0.44812982025164
N	2.12317988719526	1.16908479517545	-0.62006233934329
C	5.97266158478281	-3.08416438455243	0.85312749852971
C	2.98218207757884	-4.33620712742962	0.88364154112194
C	2.24079705848953	-2.66291879807764	-0.68457363255140
H	7.67631369912328	-0.35428823899072	3.02231586077768
C	7.93820883160575	-2.17704463554972	1.93143020344908
P	-1.60596397445609	-0.13228288581240	0.00733943955160
C	-4.08674733547199	-2.16439846145085	-0.39117655819332
C	-2.92568337721831	-4.38355098037751	0.01268800232295
C	-1.80579053397104	-6.51825045720884	0.47737609571360
S	3.18066804430293	1.43972449335864	-1.87904495162921
H	1.48860503221127	2.31599187177891	-0.27574344014455
C	7.31807318381316	-3.15409927327496	1.12691541462410
H	5.50840151726722	-3.83717185620671	0.22755790626493
C	3.62821117301611	-4.75594891671193	2.07229223197522
C	2.19553421638256	-5.29225707752573	0.16724636842170
C	1.43262085094408	-3.58040173870205	-1.39883232708036
H	8.99960956437904	-2.24712846459808	2.14597743612080
O	-2.43932664741752	-0.68084786902230	-1.27488188746480
N	-1.95975455871995	1.38610519051618	0.31796135990948
C	-3.77586283883755	-1.05514906252542	-1.16054647767414
C	-5.46186529644340	-2.52708361501322	-0.23180271198169
C	-3.67647572443619	-5.04604365508627	-0.98755106741547
H	-1.07468548438028	-7.07872222179518	1.05316947218570
C	-2.54928755018513	-7.13102547217317	-0.50058449397080
O	3.45674716220053	2.88387749042480	-1.82139564571960
O	2.71874076134849	0.82199649614061	-3.12821398881637
H	7.90816775004615	-3.96445310367998	0.71164328326231
H	4.19105075447419	-4.03382830651158	2.65141832908838
C	3.53628564066282	-6.05652658918633	2.50717293874561
C	2.14117660478353	-6.63006189042450	0.63106093374011
C	1.45420798892691	-4.88928593958640	-0.96160845075494
C	0.54659086011882	-3.17011019387055	-2.50726264482304
S	-2.64627808925383	2.06841937371914	1.60868950944419
C	-4.74104289512500	-0.36629537911496	-1.94929832545413
C	-6.44780540643923	-1.84301832358397	-1.00453525847578

C	-5.89347497381079	-3.51426658115859	0.68639632563397
C	-3.48662147274868	-6.38354181601123	-1.24259315931214
H	-4.39468975415533	-4.48107297363583	-1.57042252871489
H	-2.41649798098342	-8.18787104069956	-0.70773271662205
H	4.03320024786680	-6.35172592165717	3.42555426097422
C	2.79757931056378	-7.00855331629791	1.77519907816206
H	1.55599294944239	-7.34842653602993	0.06398651554572
H	0.86330091594748	-5.63216962614319	-1.48899065246899
C	0.92987153841380	-2.28886824636331	-3.52553945069260
C	-0.74689297721768	-3.69387145740261	-2.55267197003824
O	-2.04681718906643	1.63714640899685	2.88575628520702
O	-2.71378704858919	3.51982134500432	1.32648870488723
C	-6.04823652867451	-0.80486116325685	-1.86704633672646
C	-4.43209301473614	0.76397941861528	-2.85143128102535
C	-7.81246519454213	-2.19237111802078	-0.85818881365805
C	-7.22602899871914	-3.82525898219071	0.81154804071252
H	-5.16295064554464	-4.01521615027998	1.31013696265035
H	-4.06133035486191	-6.86752026529553	-2.02550511151467
H	2.74285896558919	-8.03454183273914	2.12402058599741
C	0.04942732617477	-1.97061496439352	-4.54989993644701
H	1.92035741766639	-1.85024539219449	-3.53011489435874
C	-1.62879762886171	-3.35353215693212	-3.56879921582276
H	-1.08033927423459	-4.35605975638022	-1.76194458403534
H	-6.80810170002165	-0.28700759715501	-2.44434089018669
C	-5.00269878915855	0.80538611452020	-4.13169079485542
C	-3.66804050417286	1.85915148446530	-2.45116912970614
H	-8.54992136919383	-1.66067332634972	-1.45224784139142
C	-8.19580895133026	-3.16789034035337	0.02808166647163
H	-7.53612778424985	-4.57125613461251	1.53577583906407
F	6.83772220061662	-2.15340187351709	-3.96822327037238
C	-1.25152462328790	-2.48960822009148	-4.59920997526677
H	0.39745667306534	-1.29954502995629	-5.32879473054045
H	-2.62696975844071	-3.77389883523898	-3.53712607964661
C	-4.79367145273395	1.88958000487111	-4.97035522858141
H	-5.59203381751137	-0.03646488845276	-4.48338398762323
C	-3.47135767659216	2.94937505648753	-3.29108743201238
H	-3.22451388098759	1.87709194324432	-1.46494957214464
H	-9.24428395242825	-3.42413418129545	0.13956343316909
C	6.70819019980322	-2.35354342844792	-2.63368564124845
C	-2.17581457857909	-2.16452932478226	-5.77431695535399
C	-4.01587708707340	2.98621439768904	-4.57470453391254
H	-5.23952705120262	1.86974940126519	-5.96013024715455
H	-2.86928182722864	3.77046237011057	-2.91963926608004
F	7.86973902647818	-2.88288072784368	-2.19438323508873
C	6.35506325293719	-1.07652330967081	-1.92258220408439
F	5.74484006442156	-3.29229435500282	-2.47361413379875
C	-2.19758202408676	-0.65159995987178	-6.05002508350577
C	-3.61871472692711	-2.61598375224670	-5.51238893640475
C	-1.64476761539220	-2.89901919668475	-7.02100546412118
C	-3.78280836944156	4.15320289293465	-5.53385828065024

C	7.29880647407157	-0.39907628161324	-1.15843267207515
C	5.07411463253983	-0.56309665488636	-2.09532826680091
H	-1.20598610919096	-0.26730318843481	-6.30497740289395
H	-2.86010317342439	-0.43571739076789	-6.89536646294694
H	-2.57585593344969	-0.10221173486396	-5.18348991077347
H	-3.69768069998371	-3.70444023000052	-5.42778527772934
H	-4.01791416776890	-2.16419537888855	-4.59719652605839
H	-4.25439466519034	-2.30529743464842	-6.34778823744772
H	-1.62181308931071	-3.98059617113860	-6.85315283684199
H	-2.28784435803954	-2.69547068380925	-7.88483380198584
H	-0.62881944882731	-2.57420161226604	-7.26559317823889
C	-3.15306425396625	3.62275917223087	-6.83610881560255
C	-2.83615913120013	5.20475215441229	-4.94118308959394
C	-5.13119350306919	4.82529060898402	-5.85187963449379
C	6.94041887797392	0.79411530126583	-0.54114617937534
H	8.29683433119395	-0.79992222028009	-1.03790203412159
C	4.74483436026881	0.63087799235899	-1.47098948174668
H	4.34980613301880	-1.06471259218000	-2.72573716607216
H	-3.80714272622930	2.90794748796536	-7.34330497415938
H	-2.96163046990944	4.45138830111557	-7.52691523329587
H	-2.20183910449223	3.12406291497467	-6.62414980082060
H	-2.68992509775478	6.00977570277556	-5.66872749751211
H	-3.24012167900475	5.64904734343186	-4.02595288381103
H	-1.85470804683888	4.77615743780596	-4.71109200723373
H	-5.82924401426965	4.12524506570516	-6.32094827434306
H	-5.59738789473320	5.20540844051453	-4.93736218850251
H	-4.98173515810229	5.66577433216661	-6.53881265801177
C	5.66006806719899	1.31874391042001	-0.68757703556761
C	7.95399401521232	1.59329004067545	0.22893692660547
H	5.38757732159493	2.26896128013330	-0.24187124593197
F	7.44303051715696	2.05701576812095	1.39848792340480
F	8.35269248116874	2.68083321194139	-0.47455923578732
F	9.06026703737349	0.88156613387891	0.52602046672825
C	-5.26556241289964	2.00650352938409	0.71598016184314
C	-4.35373350848954	1.46988428534529	1.61690628759736
H	-4.96958907274462	2.80158603550341	0.04069726631588
C	-6.55713150975304	1.49257110876070	0.69038039562200
C	-4.71364661470428	0.46266987605733	2.49860706230610
C	-6.94462565511470	0.48058083425267	1.56522976131673
C	-7.55844379025389	2.07094846330064	-0.27155972198768
C	-6.01802976572859	-0.02322025071195	2.46899230486985
H	-3.98863916486677	0.07097812857999	3.20127751661319
H	-7.95069448161559	0.08110472281825	1.53122589221734
F	-8.18053693879708	3.15503359876535	0.25505185107991
F	-8.52738603612097	1.17766935985832	-0.58776353895111
F	-6.98081759388360	2.46637795822008	-1.42487090294858
C	-6.41624653665598	-1.06437611685446	3.47792538723402
F	-5.46257234305285	-2.01494198651420	3.61792862729421
F	-6.59497878874878	-0.50918580388211	4.70449959340388
F	-7.56980518829833	-1.68317743076653	3.15439670622717

H	-2.06499114633098	4.67305342610804	-0.20802185701326
H	-0.82366496941774	6.27256731508919	0.55147013352083
H	0.61021999140785	7.54131444253946	-1.14381699262123
H	2.15974342845921	5.66248799733860	-1.65964519762730
C	-2.74557137373645	1.18152878405864	7.25286943682146
C	-1.88600352985323	2.42745054301605	7.02448333913742
C	-3.66395326432321	0.93768560805517	6.05331941470701
C	-4.55017981890226	2.15685671920095	5.78380320869258
C	-3.70506495696001	3.41685939055770	5.57693708763295
C	-2.76232762967565	3.65510728321837	6.75984687424021
H	-2.11390983314531	0.30302429633512	7.43340863831951
H	-3.35432732413303	1.32219514866369	8.15734316507528
H	-1.22239505012835	2.60407484814435	7.87996851980282
H	-1.24133543878758	2.25444430339349	6.14981358560425
H	-3.03541295398514	0.75626710040726	5.16995723764398
H	-4.27745157951718	0.04293855972008	6.21069417058080
H	-5.18385461106767	1.97996562751730	4.90670641577159
H	-5.22755888403564	2.30387063007631	6.63722662036447
H	-3.11105439792942	3.29493331821087	4.66171862309854
H	-4.35145432531824	4.28918979547010	5.42460479498618
H	-2.13626233673129	4.53631151382016	6.57373140176935
H	-3.35472012788424	3.87159477685008	7.66037352820300
C	4.69278430218474	3.47750764277005	-5.32640519608840
C	4.74264366986563	2.23924394263852	-6.22518780622760
C	6.09070804330164	3.86965735372724	-4.84051152675081
C	6.77265382681455	2.69948667779149	-4.12808221777612
C	6.83188904398847	1.46262458745068	-5.02780718338827
C	5.43738409644697	1.06946934658056	-5.52241574623966
H	4.23305265284158	4.31624911250276	-5.86495848462376
H	4.05474887603626	3.26792016596566	-4.45766758474760
H	3.72697923868037	1.94764724866308	-6.51974365755649
H	5.28850930139414	2.48295959744088	-7.14838841592000
H	6.70356714039573	4.17511411772912	-5.70118767213233
H	6.02875983346904	4.73531483138706	-4.17046030241291
H	7.78010901879720	2.98355011398806	-3.80084201260186
H	6.20112091493806	2.45965610147050	-3.22090526166747
H	7.47658858179942	1.67625954532169	-5.89256600455558
H	7.29219358005030	0.62368346620200	-4.49151640376268
H	5.50431939221311	0.20601008506548	-6.19529404589659
H	4.82117897373002	0.75887707855317	-4.66871310315045

282

Minor0 3 CyH

C	0.68632117574889	-1.69892281180763	-4.16408250077728
C	1.11207055235044	-1.59991370224023	-5.59222022500093
C	-0.01217715641263	-2.08891857397271	-6.53794640326039
C	-1.33962183420191	-1.37869950618772	-6.31243286041381

O	-1.91455886331507	-1.82523793020206	-5.08452265638147
H	1.34362486664939	-0.55467036586837	-5.81953028490493
H	-0.17345897335063	-3.16523718780485	-6.40553279844628
H	-2.02139337982503	-1.61029688031583	-7.14471973123928
C	0.62304965097118	-3.00899103772536	-3.52001426177761
C	1.24664747473716	-4.13455264227110	-4.08883971633586
C	0.03519256837717	-3.15403502534670	-2.25455621619544
C	1.30289878384687	-5.33678728453691	-3.40207784021065
H	1.73259830279780	-4.06341387978702	-5.05407157035369
C	0.09805984042494	-4.35164419227420	-1.56484576374228
H	-0.49042649171895	-2.32275228249875	-1.80627609104506
C	0.74038700933051	-5.44617608505989	-2.13212637600466
H	1.80779762082537	-6.18664561306717	-3.84898260958420
H	-0.35236160585337	-4.41895555481695	-0.58185779606950
H	0.80587587393365	-6.38381376010895	-1.58864478450349
C	0.45509422941905	-0.52902637886808	-3.45676972558809
H	0.50758652650604	0.41041731336248	-4.00203305562311
H	-0.28845124092857	-0.54534412956451	-2.65993243504138
H	3.33456953349134	6.84462443395086	-0.68214758074231
C	2.84390048212341	5.96683957993285	-0.24758531435180
C	1.44186043638586	6.34951892120123	0.26182375426273
H	3.48372187836784	5.58726345959744	0.55456135132268
H	2.77451641285058	5.19337046724901	-1.01780047068535
C	0.77409587146201	5.13647057751506	0.90904640156273
C	0.63604316990427	6.89044707511238	-0.92424127904813
C	1.56860641715274	7.46197819239727	1.32025218495496
C	3.21756314818662	2.49440827804731	-3.34282104139000
C	-0.47361584543349	4.64783135918781	0.51499396838876
C	1.41029907709968	4.46941042568121	1.96415756601728
H	-0.35431358537161	7.24051904530235	-0.61570342806713
H	1.16431181259874	7.74080226078109	-1.36678263208181
H	0.51092815670485	6.13186571879408	-1.70479507856211
H	0.58191997262096	7.74937258909023	1.69730693449820
H	2.17248324602089	7.13576759270500	2.17213556286810
H	2.04460894872320	8.34708398863151	0.88389331040042
C	2.68499231921734	2.59267971888724	-2.05362853522761
C	2.44093852292499	2.76714676637708	-4.46481199364540
H	4.25140201852802	2.18813406521208	-3.47523363965364
C	-1.06124304669977	3.55120223983107	1.13855497101815
H	-1.01241618445911	5.11195566223971	-0.30375347639520
C	0.82739925593261	3.38379894861743	2.59436154245633
H	2.38547911753255	4.80051465279982	2.30818188984333
C	3.54803147607954	2.37169489944655	-0.87620762127341
C	1.33665712313621	2.95413654414279	-1.93389591737331
C	1.10414896058593	3.16030308432380	-4.34806085102011
H	2.89886411605907	2.66662876472662	-5.44196845673377
C	-0.42498692062361	2.89869381671474	2.19875162834502
H	-2.03177575574880	3.22026233454452	0.79277159493477
H	1.36088943147447	2.89616455987106	3.40108561073206
C	3.09930990420170	1.68748400985059	0.28091292657802

C	4.84796061168553	2.83563964984561	-0.85513246434125
C	0.57450418271399	3.24143568986676	-3.05447025207441
H	0.88088319661599	3.04597604103034	-0.95729189273348
C	0.24088053108745	3.54516803964842	-5.54961391491108
C	-1.06499595614666	1.81327999225621	2.96893794967417
O	1.77669754022068	1.23918515245613	0.29545419159410
C	3.88604641402254	1.42645776151002	1.38901773304159
H	5.21846980761854	3.41117333213646	-1.69762828354586
C	5.71995057633764	2.57860611074243	0.22231855631190
H	-0.46193438502186	3.52005618196117	-2.89922059927814
C	0.87747423378118	3.12582707119979	-6.88033160583824
C	0.07241004378878	5.07741750364585	-5.54064623229421
C	-1.14415731873943	2.88388371343191	-5.44829682044308
C	-1.93117879970542	0.85180650572636	2.38617902872307
C	-0.89198837902607	1.73019499263761	4.33622811665640
P	1.56605496036413	-0.34114293614192	0.04052075158746
C	5.25520354032156	1.83251439102929	1.35085353927053
C	3.29630070365512	0.73041022265804	2.55969696618790
C	7.06593746348902	3.01800262254083	0.19329730188150
H	1.05292338704172	2.04439492233367	-6.91585859498015
H	0.20395417872194	3.38333380449988	-7.70348885820600
H	1.82793345269832	3.63678873216608	-7.06337375505064
H	-0.54809410648139	5.39743660192696	-6.38549099070654
H	-0.41000664506616	5.41039188700129	-4.61654966504008
H	1.04461650146377	5.57570257495494	-5.61471669038556
H	-1.05770690689595	1.79468320579251	-5.39259412869892
H	-1.69889849136652	3.20635751962283	-4.56458024954879
H	-1.74535155147571	3.13774021151870	-6.32796184299145
O	-2.05455436269451	0.84853335726066	0.99720457977755
C	-2.68661906612480	-0.05482321241183	3.10137301255869
H	-0.25541071210365	2.45310272916729	4.83683926923255
C	-1.52314280454812	0.74102044146984	5.11677270999912
O	2.57891639651774	-1.01311725365942	1.09940913918486
N	0.08165694604229	-0.67654296163433	0.35276624805409
N	2.20135051488337	-0.73606649729182	-1.41516673838554
C	6.18218306385292	1.49822373933031	2.36649415568798
C	3.31281675336169	1.30479423356623	3.86972611140117
C	2.62722422241866	-0.46739269659452	2.38722395714319
H	7.40489756101465	3.59403297094392	-0.66259964160424
C	7.93243662103526	2.70238958341660	1.21065211687106
P	-1.45862001888433	-0.40694326903921	0.16589837184815
C	-3.71510801811347	-0.88709285779024	2.43090157345457
C	-2.45133181801986	-0.15994128901417	4.50823744721291
C	-1.25320372072010	0.62506802837767	6.50235870629816
S	3.37389891044441	-1.88930339683818	-1.69023882537247
H	1.42925712371158	-0.57309882671320	-2.51310529316049
C	7.48787265386896	1.92321108011302	2.29771646390750
H	5.85351715733002	0.89227351444123	3.20238593297924
C	3.85067147037012	2.58645810180259	4.14315650106381
C	2.68479897364739	0.59262785496055	4.93958219234847

C	1.97323255721825	-1.18210410344661	3.41984074149841
H	8.96384696716338	3.03732162714162	1.17153989825681
O	-2.09113216318453	-1.71638675288442	0.88928368196806
N	-1.97077377295432	-0.26915598512984	-1.33230429487443
C	-3.40056117561435	-1.71341874640118	1.36387028146850
C	-5.06933140697858	-0.84802851045063	2.89389623217170
C	-3.03621117118430	-1.16780951898283	5.31145416790606
H	-0.55681654361014	1.32736747597437	6.95198142401827
C	-1.84148343879670	-0.36105742808264	7.25471500676009
O	3.50645719848446	-1.92955724066361	-3.15540524929672
O	3.12572301980271	-3.12375408991127	-0.93539872597654
H	8.18372505150465	1.64735338546709	3.08312554887024
H	4.28827847471669	3.15924662765488	3.33451517724146
C	3.80561514528235	3.11809186675601	5.40966988384125
C	2.67721186217556	1.15964341607143	6.23833475697208
C	2.04423148268127	-0.63683444378662	4.68580271890566
C	1.18537357948511	-2.40635227787838	3.16970576016115
S	-2.84738497495015	0.88025593025336	-2.04877078054818
C	-4.31660412829648	-2.65895684910691	0.82043164884648
C	-6.00814299409428	-1.77726239172795	2.35315781450152
C	-5.53047468030112	0.09720980851599	3.84118053629427
C	-2.73232555921117	-1.27011606630170	6.64824034338475
H	-3.71816833812660	-1.87586545517196	4.85528939752059
H	-1.61990774478008	-0.44753902435857	8.31342154673851
H	4.21553008437148	4.10616221420435	5.59189908367332
C	3.22617966871494	2.39523769340102	6.47268237207171
H	2.21292167590973	0.59683026138121	7.04311037468389
H	1.57161794735266	-1.15836438335052	5.51266606919089
C	1.61267304736323	-3.45175352612759	2.34238547447893
C	-0.05473887002507	-2.53926628852146	3.79792453018322
O	-2.38036156681966	2.24662884419286	-1.74531829090477
O	-2.96979986306414	0.48538107962494	-3.46970075081702
C	-5.58969056254210	-2.67781919004484	1.35580737738924
C	-3.99305517147170	-3.62087906937814	-0.25532322894862
C	-7.35246643731521	-1.74855308993770	2.79772035445166
C	-6.84344725955422	0.10599410262079	4.24634627248945
H	-4.84226825901635	0.83734691834916	4.23124577541275
H	-3.18056269458200	-2.06026808381458	7.24171705314491
H	3.20757573558456	2.82275854553161	7.46977091269911
C	0.82876382792777	-4.58529258176980	2.17789263449599
H	2.56382050767078	-3.39216409852310	1.82703711403185
C	-0.84254057447872	-3.66622060800090	3.61057625197865
H	-0.42576334011846	-1.73264027099766	4.41998445690908
H	-6.31578070379623	-3.37400034228190	0.94730730794890
C	-4.43374000156381	-4.94827981880781	-0.15131549620955
C	-3.35236914341842	-3.24619672611980	-1.43578558510977
H	-8.05351319624739	-2.46089293417910	2.37279155116738
C	-7.76325815612980	-0.82875874398981	3.73002003857165
H	-7.17901324205047	0.85354067815227	4.95756715572868
F	7.59667614855595	-3.21836220414091	1.64758867008333

C	-0.41838083454117	-4.72333501630809	2.80232578360166
H	1.21215237789283	-5.37976225811835	1.54541958259496
H	-1.80572736281290	-3.70040077914887	4.10573558283232
C	-4.22359841603102	-5.85219102644966	-1.18115507537054
H	-4.92303471299107	-5.28057907283775	0.75958160961670
C	-3.15548682898183	-4.15317390714543	-2.47099629345241
H	-3.01052653969128	-2.22859651405433	-1.56657182754195
H	-8.79693397828451	-0.80758958198241	4.05926710611972
C	7.28936777204221	-1.90492931788326	1.78345283482556
C	-1.23082925956244	-6.01086892227533	2.65062375754600
C	-3.57450439287492	-5.47927755534666	-2.36610342905634
H	-4.56583085743358	-6.87405047371128	-1.04882917587291
H	-2.65693420810978	-3.79750407831595	-3.36536065900489
F	8.41360771615241	-1.27148836666261	2.18018365847677
C	6.73541513511881	-1.34321106885804	0.50359879791141
F	6.39211319757353	-1.81696790637128	2.79407363431935
C	-1.31741522959585	-6.43653551482244	1.17537176861420
C	-2.66275372876657	-5.84967649068113	3.17890917059890
C	-0.52610163199958	-7.12027768495718	3.45596677206572
C	-3.34205531554902	-6.50716247462493	-3.47276117318159
C	7.51768435374240	-0.53975556906412	-0.31856133603548
C	5.44591291665920	-1.70653485872248	0.13021229519658
H	-0.33027027223075	-6.63352881321580	0.74785549124899
H	-1.90194987404540	-7.35892776091568	1.08813903227620
H	-1.81457855468532	-5.67003003253038	0.57471105981019
H	-2.68376747229781	-5.67709361843886	4.25958584912723
H	-3.17849668009063	-5.01997848799958	2.68274363670563
H	-3.22889747396984	-6.76576463357226	2.98260465361322
H	-0.44986405973049	-6.84355548693238	4.51238173485370
H	-1.08808721359657	-8.05827471565168	3.38211739022304
H	0.48614738701395	-7.29634981561989	3.07917432835234
C	-2.52733349601493	-7.68531693413340	-2.90629183055352
C	-2.56669735198366	-5.91455129996136	-4.65607048051809
C	-4.70186325501903	-7.01969528518331	-3.98214488538735
C	6.98761775845225	-0.07923386261018	-1.51827888165933
H	8.52385002136234	-0.27023573926543	-0.02471614762520
C	4.94307838408692	-1.23550597791904	-1.07368574951944
H	4.84915908637648	-2.36746455042313	0.74714619251501
H	-3.05441984322849	-8.19151280550852	-2.09227982874768
H	-2.33571047372666	-8.42371982286892	-3.69281912572488
H	-1.56457952994078	-7.33373411847186	-2.52112338764318
H	-2.41383445889129	-6.69018076338707	-5.41362222718724
H	-3.10932527248319	-5.08851785308510	-5.12655919276536
H	-1.58218537137671	-5.54671472900209	-4.34795580820132
H	-5.28001410203689	-7.49182988669441	-3.18230563977046
H	-5.29686108462309	-6.19456458403911	-4.38631413378940
H	-4.55436539052298	-7.76062845133500	-4.77583469999412
C	5.69548665932336	-0.42029762434578	-1.90683133035645
C	7.82564510576198	0.73258075307708	-2.46558133508549
H	5.29096072880038	-0.09630303703163	-2.85938272795578

F	7.14058607445183	1.80030772014528	-2.95092882958990
F	8.20028771017817	-0.00376952350899	-3.53953118165696
F	8.95123103773422	1.20326605705898	-1.89115192194891
C	-5.32245163938403	-0.31558102759179	-1.73172035782930
C	-4.50238104337631	0.73389329564380	-1.33295920302559
H	-4.99066263529505	-1.01590940755281	-2.49017738095039
C	-6.56586498765045	-0.45621944645587	-1.12688593422764
C	-4.90940939456908	1.64738246404378	-0.37350809826527
C	-6.99908651612131	0.44735026283334	-0.15924334399080
C	-7.46583574970626	-1.58601105654422	-1.54728583982789
C	-6.16696424327451	1.49701452230623	0.20624843999633
H	-4.25687231387482	2.46479812143322	-0.08915636480693
H	-7.96392623893385	0.32239474350638	0.31627475409875
F	-8.23250152597355	-1.24155616855434	-2.61128741856736
F	-8.31370808523149	-1.94874195568089	-0.55341364824968
F	-6.76512862802671	-2.68307465923752	-1.90063730212251
C	-6.60767662897588	2.51881840393772	1.21625568195859
F	-5.65096299226960	2.73220881730856	2.15066967592254
F	-6.84703873666077	3.71871268216066	0.62706672229637
F	-7.73689048058911	2.15998036789948	1.85878466036463
H	-2.25098117537061	-1.06195801920239	-4.58816641098922
H	-1.19030901845376	-0.28913846851422	-6.30364322936238
H	0.31604497559560	-1.92908322463875	-7.57196769877753
H	2.02152732462993	-2.18150568078826	-5.77365954761308
C	-3.61876071354945	5.87929388520783	-3.92630627526724
C	-4.41278234806743	4.63645545412175	-3.51651139551419
C	-2.67404956760324	6.32623050827864	-2.80736628175677
C	-3.44115064988834	6.57071841841191	-1.50545161114247
C	-4.21435740879499	5.31750111403274	-1.09043382931021
C	-5.16434060296529	4.86407550018964	-2.20243236989519
H	-3.05359962020241	5.68279818364420	-4.84568678716779
H	-4.31648127454583	6.69840708906521	-4.15241470867448
H	-5.11390292497921	4.35547948460587	-4.31131722528938
H	-3.72359073616350	3.79199487752380	-3.38575065699256
H	-1.92570791953836	5.53788259502009	-2.63415321547813
H	-2.12161900457030	7.22601546576069	-3.10536311110744
H	-2.75632560457353	6.87527537095752	-0.70482522249469
H	-4.14447276910315	7.40318823737927	-1.65001498482272
H	-3.49065983166832	4.51423208293348	-0.88854407019632
H	-4.76903520570162	5.49730423178689	-0.16188676149209
H	-5.68773435237478	3.94775134503726	-1.90603892883950
H	-5.93689046031086	5.63283834470140	-2.34841232087740
C	6.41711707534936	-4.90999740821756	-1.60114880426640
C	7.46805628168114	-4.14358603757943	-2.40939989813438
C	5.71457827569257	-5.96664259268570	-2.45851466159497
C	5.10206499121451	-5.33655119526356	-3.71178406054472
C	6.16227006516400	-4.59251165058179	-4.52777865955439
C	6.86248309166884	-3.53102111850659	-3.67520082414145
H	6.87959680477673	-5.37471894828879	-0.72212589671525
H	5.66085821787885	-4.20788764319592	-1.22635140650476

H 7.92411201883328 -3.36078246466729 -1.78944496641879
H 8.27632207803562 -4.83260012885075 -2.69350442957964
H 6.44311469289455 -6.73446388428393 -2.75706254344433
H 4.93885429663016 -6.47143101069689 -1.87043260723351
H 4.62097933014138 -6.10679090668672 -4.32790022893903
H 4.31892026873683 -4.62774656560599 -3.41065929770659
H 6.90728541642613 -5.31118657805294 -4.89932224225275
H 5.70498073142297 -4.12466514486503 -5.40787337017015
H 7.63712884653207 -3.01878843351645 -4.25816520681891
H 6.12158766610789 -2.77239634555421 -3.39226756729534
C -3.63070207039422 4.52382541069232 3.94640904266458
C -4.31910198692476 5.66993432219261 3.20023934176578
C -2.60347142200988 5.05748497337856 4.94814022685287
C -1.57827568723139 5.96424743301514 4.26072505432329
C -2.27051745983969 7.10986157195873 3.51773545349324
C -3.29187118497879 6.57248790540940 2.51258173504499
H -4.37353456327687 3.89936791660727 4.45749074528339
H -3.11853635617434 3.87618725998727 3.22090338917418
H -5.02944924350315 5.27383041713931 2.46645900108973
H -4.90186053248140 6.26717203416849 3.91646937854829
H -3.12586310537239 5.62910994692489 5.72893848249352
H -2.09885043829147 4.22329766312308 5.45075613661310
H -0.86662198063962 6.36087200559680 4.99510845070701
H -0.99691380834137 5.37151962409604 3.54148893815408
H -2.78433201956023 7.75625715120792 4.24405627387338
H -1.52786622477230 7.73579060440564 3.00846345794162
H -3.79459912598865 7.39911334065441 1.99577237004183
H -2.76218309759127 5.99093082310922 1.74508531732848

300
Minor0 4 CyH
C 0.70625791413754 -1.49897937444940 -4.49891155245964
C 1.04923965589718 -1.36617332673476 -5.94725228941368
C -0.00392007791067 -2.07588669581893 -6.83359928651116
C -1.42900662784312 -1.62749283153629 -6.54397952107580
O -1.84495217914088 -2.16699191375651 -5.28993878440752
H 1.07513651144641 -0.30348703647797 -6.20719766354237
H 0.05002594471491 -3.16108417927858 -6.68808192223947
H -2.09468056412751 -1.98755944657751 -7.34310932537500
C 0.91392923278660 -2.77850948634336 -3.82403860091819
C 1.69839403788853 -3.79093315773200 -4.40707513503250
C 0.42269487251513 -2.99182398557703 -2.52758410792372
C 1.99450999260752 -4.94996416074748 -3.70876706925196
H 2.11499572639066 -3.65841318140610 -5.39757583926628
C 0.72309609816596 -4.14716655542959 -1.82679771160598
H -0.21661481589746 -2.25328779596426 -2.06450264443893
C 1.51489591044475 -5.12819436977462 -2.41254397610812
H 2.61623026007848 -5.71146680245288 -4.16831839051523

H	0.33449637476501	-4.27554804898691	-0.82337439618029
H	1.76054774394630	-6.03261151641953	-1.86511536308011
C	0.28981507779235	-0.36837292385670	-3.81140869864594
H	0.14671491277790	0.54556905987236	-4.38355033878503
H	-0.40469672910525	-0.49033694379483	-2.98043859086085
H	1.71600981111023	7.59935383989194	-1.47196820184000
C	1.42974725138777	6.65108659961117	-1.00393600429155
C	0.00490572607053	6.76213345873642	-0.42877458593799
H	2.17010944734581	6.41966151312771	-0.23284471210094
H	1.47638096905092	5.86498434636351	-1.76318645286284
C	-0.36908949131610	5.46981112599935	0.29851829589510
C	-0.94994874354545	7.07098818506629	-1.58734342766906
C	-0.04930047770044	7.92532555241610	0.57995978593449
C	2.40138969918601	3.14223501714129	-3.91781903862020
C	-1.50680158843187	4.72010554511310	-0.00944611265891
C	0.43994866506384	4.99280375830482	1.33840233576322
H	-1.97591928788835	7.22568361175734	-1.23807728711599
H	-0.62841448473609	7.98872144231190	-2.08959799544769
H	-0.95024888299501	6.26506295439078	-2.32931510334435
H	-1.05292188739187	8.01939043267497	1.00629877142786
H	0.65326052929961	7.77340050211935	1.40485800245724
H	0.20639132959657	8.86820838375410	0.08399369678713
C	1.91446395245140	3.20728595866117	-2.60863389210892
C	1.53850880850603	3.18925589119148	-5.00890180432443
H	3.46908184196354	3.03734631265503	-4.08780696718398
C	-1.82518377156711	3.55281094340021	0.67873333547686
H	-2.16727488444453	5.02984440553499	-0.81131804714886
C	0.12149405562707	3.84074728536856	2.03612795563939
H	1.34198685722442	5.52929498439683	1.61709832256024
C	2.84890232568542	3.20959328368990	-1.46499544552117
C	0.52847618501831	3.30900167213759	-2.43531036363731
C	0.15564594139024	3.31430663254596	-4.84039416653736
H	1.96564289542911	3.12130596014705	-6.00278614157512
C	-1.02156365789487	3.09378673732464	1.72615276236089
H	-2.71911890430477	3.01294675904854	0.39337585475875
H	0.78073829972039	3.50592185463825	2.82762217334496
C	2.58609167928151	2.49016682460587	-0.27305311342424
C	4.02900588158818	3.92399373942471	-1.50897001439294
C	-0.32294265131784	3.37334608448277	-3.52606115054282
H	0.10578950096697	3.36797304921091	-1.44168209831079
C	-0.81776694436420	3.43775658124583	-6.01320685348532
C	-1.38923463463149	1.94071110605776	2.57239414144258
O	1.38236711037652	1.78738912620017	-0.19757949080994
C	3.44127815477608	2.43498222428409	0.81395337739631
H	4.24736302925598	4.53232871808182	-2.38107754793156
C	4.96987253306197	3.88290371922590	-0.45916159587307
H	-1.38631790250843	3.45158212582241	-3.33111455796002
C	-0.18428143484786	2.99278471114260	-7.33738740271805
C	-1.23313843609528	4.91777674716483	-6.13091539922701
C	-2.07156961342844	2.58068006786802	-5.76771423178028

C	-2.06157471339041	0.79229014142544	2.07868429231219
C	-1.14292785444967	1.96962673519095	3.93082903885153
P	1.48569293612066	0.18904810376146	-0.39345619566665
C	4.69986066713279	3.10228708537652	0.70923886028225
C	3.03580305995808	1.68177412861795	2.02708763775500
C	6.20088922714569	4.57693275144016	-0.55385541109370
H	0.17404513285795	1.95843580288581	-7.27790685396437
H	-0.93150367066692	3.04418807048490	-8.13520643373439
H	0.65324700888853	3.63233519846391	-7.63256002694268
H	-1.93249344485112	5.05256070891775	-6.96401991708214
H	-1.72393618666678	5.25781808983257	-5.21342378092866
H	-0.35911822574778	5.55406472843663	-6.30454203336723
H	-1.81006963352602	1.53086139091108	-5.60549271807252
H	-2.63819033511436	2.90656894255528	-4.89256670218114
H	-2.73982690117464	2.63940341466596	-6.63355267263581
O	-2.24729669138184	0.69124785060234	0.70000581936266
C	-2.57803564415629	-0.21034842201746	2.87418979656584
H	-0.65348285654596	2.83707705778556	4.36267093740637
C	-1.51056537923831	0.91117996934788	4.78557197570309
O	2.65016607000879	-0.21977170580168	0.64390992947243
N	0.11264869040104	-0.42426255179770	-0.00634850981985
N	2.13572159724812	-0.13096306500013	-1.86210047060598
C	5.70733140476696	2.99571682048210	1.69735462008516
C	2.97561817615994	2.29114146358076	3.32004292579658
C	2.61405448423566	0.37022701622508	1.91215242314129
H	6.39101573816712	5.17632848733095	-1.43912666898428
C	7.14676786072995	4.47411391177347	0.43622784324060
P	-1.45739653813481	-0.46709579103034	-0.11152402792898
C	-3.44929337842366	-1.26690797285068	2.30562162619460
C	-2.25252054088231	-0.19462974147353	4.26706714811621
C	-1.15753994351034	0.92528292717934	6.15710829594955
S	3.47925145572766	-1.08192320073459	-2.13697461326833
H	1.29741517033069	-0.18618554999815	-2.92664879625959
C	6.90132994693946	3.66392573119357	1.56307620168427
H	5.53281924627191	2.36960265785015	2.56435079282047
C	3.27028958734479	3.65853457592900	3.54373791666575
C	2.51853695004392	1.50869755453631	4.42731247850356
C	2.13471693367924	-0.42101196275560	2.98355725035706
H	8.09013130324868	5.00257747901801	0.34510457800346
O	-1.77676868777906	-1.83552005880524	0.70079572529270
N	-2.05872978823461	-0.51196280649391	-1.58219213240231
C	-3.03012696748026	-2.07023437211164	1.25800230209011
C	-4.75416750142628	-1.48176688203852	2.85444612766223
C	-2.56797097130188	-1.26317334561734	5.14027223600963
H	-0.61122241704490	1.78274529795099	6.53961739943336
C	-1.48238137049542	-0.12525753503180	6.97896298153448
O	3.57026973397740	-1.17757623214483	-3.60114603774253
O	3.46910554877411	-2.29601763401388	-1.31168400775024
H	7.66366041197706	3.55909251312321	2.32805501884198
H	3.56960702708165	4.27870356718821	2.70734448339603

C	3.16399569728889	4.21064506586170	4.79792167612149
C	2.44508444508183	2.10285483490428	5.71147539329019
C	2.11578397190240	0.17327528811334	4.22867699436204
C	1.65421126751630	-1.80373394662264	2.80154083195662
S	-3.18229410517262	0.39960172643407	-2.29570237640497
C	-3.75403409258128	-3.21488906322481	0.82098103209227
C	-5.50571718485675	-2.61467426294056	2.41910177208145
C	-5.34909539358818	-0.59989708337086	3.78790508889271
C	-2.18408795813066	-1.23263308335552	6.45987775002815
H	-3.10381400854197	-2.12147226319440	4.75185286220093
H	-1.19751529153183	-0.10951232610267	8.02590043107590
H	3.38797811922978	5.26259748877730	4.94179622118083
C	2.76237503407236	3.42493580758755	5.89762799726228
H	2.11920314024322	1.48693212848207	6.54480875031324
H	1.77450619638493	-0.40200922072465	5.08407919847688
C	2.32943931937988	-2.74272909933411	2.01396721244922
C	0.51190305772984	-2.22539573914138	3.48345038451820
O	-2.98225316249196	1.84634525030114	-2.08692119150595
O	-3.29736037678075	-0.09369624348654	-3.68621017982823
C	-4.96237525580336	-3.46750795937792	1.43919415960000
C	-3.28931819445604	-4.14708181267026	-0.22925410260816
C	-6.79901643108674	-2.83837914944872	2.95065042262282
C	-6.60959911650522	-0.83949884071406	4.27977986080817
H	-4.80998703350613	0.28696652017219	4.09865932624405
H	-2.42179270982165	-2.06993979908595	7.10783629097780
H	2.69657332111451	3.87036712602214	6.88483920972820
C	1.88567940461853	-4.05381953390118	1.94185393408285
H	3.21920985175628	-2.45716640525681	1.46725024535331
C	0.06199216532500	-3.53638600799175	3.39230010934579
H	-0.04541386755420	-1.51429574212124	4.08281287333116
H	-5.54460637525595	-4.32459978633541	1.11465644933323
C	-3.39848380084484	-5.52972205908521	-0.02420555444014
C	-2.84118833055316	-3.71362244112326	-1.47628478586308
H	-7.35792857618998	-3.70327185454646	2.60557693825149
C	-7.34041419345773	-1.97153699648476	3.86701721862098
H	-7.05380871580032	-0.13854486547498	4.97878415303011
F	7.92341192939028	-1.43659400511455	1.12910430939520
C	0.74298684030041	-4.48718993090756	2.62792938603464
H	2.45856994478693	-4.75243541895475	1.33949704927935
H	-0.83803366394917	-3.80540786710187	3.93279078582328
C	-3.07049067002378	-6.42739830918272	-1.02900758998762
H	-3.72542904025255	-5.90361220578303	0.94188149628343
C	-2.52721278784015	-4.61739525105036	-2.48528340774949
H	-2.74735652634701	-2.65524520147122	-1.67860944988465
H	-8.33537544700386	-2.14604910911444	4.26326947417400
C	7.42043522485980	-0.17844891296002	1.19129852849370
C	0.31941562639331	-5.95615031901218	2.56320887198059
C	-2.62901198261913	-5.99449482489376	-2.28679843911709
H	-3.15722954076297	-7.48936651340957	-0.82036253078212
H	-2.19095422987690	-4.21685842395991	-3.43464267213247

F	8.44371085751363	0.64648236894096	1.49563575563522
C	6.73967275089033	0.19747137160000	-0.09532175493229
F	6.55482369517822	-0.15807574265317	2.23329692226474
C	0.17980154407621	-6.41210558517162	1.09996288526762
C	-1.02141001237073	-6.20240054633864	3.26519323893816
C	1.40365429945232	-6.80380446326441	3.25664279114600
C	-2.28121326024925	-7.01283697835845	-3.37234201403607
C	7.30952211330489	1.12165349012307	-0.96369002656759
C	5.54720605411830	-0.44372245809181	-0.41440963124246
H	1.11980400338877	-6.30388967750844	0.55112330608079
H	-0.10309239761642	-7.47009101727702	1.06416999172626
H	-0.59699276818452	-5.84029457373830	0.58317682549998
H	-0.96568268165499	-5.98622353836330	4.33686777386448
H	-1.81814221426790	-5.59201445659442	2.82598467388301
H	-1.30345751372309	-7.25413863085039	3.15341599223964
H	1.52175666485304	-6.50043739978122	4.30197616511879
H	1.13006480240004	-7.86465776938261	3.23197701585237
H	2.37256241387437	-6.69021241223437	2.76022911708480
C	-1.18770433720798	-7.96362894515229	-2.84981698053484
C	-1.76291844734749	-6.34052578234662	-4.64979420079910
C	-3.54088487175332	-7.82667943039729	-3.72387848114300
C	6.66238339380379	1.41668268387876	-2.15890499971132
H	8.23962385327204	1.61222262115157	-0.70795162557778
C	4.92194973320574	-0.12838664792044	-1.61198698255775
H	5.12079499176392	-1.19344729306096	0.24205532295444
H	-1.52059477518149	-8.52148438649206	-1.96987891654781
H	-0.91868088717571	-8.68928036380434	-3.62527576040292
H	-0.28977152422592	-7.40063986439122	-2.57666420214563
H	-1.52035485222492	-7.11008071514216	-5.38986644479971
H	-2.51115405597934	-5.67523702332657	-5.09204497760258
H	-0.85521562305878	-5.75789283874080	-4.46039288881244
H	-3.93040026117502	-8.36349993907933	-2.85374243522850
H	-4.33284724249251	-7.17012799391411	-4.09792468011881
H	-3.30788321951483	-8.56428642957303	-4.49985096376697
C	5.46202561318796	0.79726076530916	-2.49365365148021
C	7.28236108922901	2.35862125494842	-3.15331721861537
H	4.96732232393747	1.00160177251877	-3.43682981385044
F	6.37208356162183	3.23691032717119	-3.64661825306005
F	7.78287970983367	1.68584571138657	-4.21804749675007
F	8.29318135933680	3.07827961320342	-2.62527486929384
C	-5.35324616869659	-1.23763461829907	-1.77583899068927
C	-4.73462214815083	-0.02961724065923	-1.47207870275469
H	-4.93668641838733	-1.89424525052578	-2.53120189450769
C	-6.50350876239869	-1.58940770904796	-1.08034927346978
C	-5.25518085373009	0.83169236408271	-0.52003144574544
C	-7.04758047456094	-0.74176193532343	-0.11753937549483
C	-7.19102565281611	-2.89000682482016	-1.39426346580476
C	-6.42053416774658	0.46725381690070	0.15041501629005
H	-4.75819086077454	1.77068936787317	-0.30664401297288
H	-7.93780994215642	-1.03119456557405	0.42711971023675

F	-8.12421307853670	-2.73514627695636	-2.36622160031718
F	-7.83496100322147	-3.39083403300920	-0.31104628616003
F	-6.32384617847999	-3.83054259194027	-1.82011501488023
C	-6.99233566273970	1.42973471015534	1.15292842292087
F	-6.03813383398149	1.88347765353571	1.99948195079885
F	-7.51782361314802	2.52251737776552	0.54120429770918
F	-7.97442212222021	0.88082513553729	1.89514536229121
H	-2.32755431707017	-1.48787526205490	-4.79162489808657
H	-1.48566567959796	-0.52897662526714	-6.53737405799406
H	0.23844002913844	-1.87105102555743	-7.88317867608622
H	2.04204189235282	-1.77660695385567	-6.15673786750449
C	-6.32122361502633	3.90919694420847	-2.46183606151806
C	-5.40704984136374	4.55263533951762	-1.41492575231229
C	-5.59069091589692	3.71663125921291	-3.79383784024071
C	-4.99876225850700	5.03495739462046	-4.29956352107136
C	-4.08744847089530	5.66938205008025	-3.24510299907652
C	-4.83767067370653	5.87715909661711	-1.92722290915245
H	-6.70065704308792	2.94701093735739	-2.09815901127010
H	-7.19758988905396	4.55526320473180	-2.61504073530144
H	-5.94731238939990	4.70373945501968	-0.47301133934503
H	-4.57019731882541	3.87087337845355	-1.20557347970458
H	-4.77937240282889	2.99084913676472	-3.65071237603811
H	-6.27055458053596	3.29317475539571	-4.54248088738886
H	-4.44326930172993	4.86926340266840	-5.23089919428209
H	-5.81244620238167	5.73536978233356	-4.53657561419473
H	-3.23260942327341	4.99947289812079	-3.06601092545773
H	-3.67680924536640	6.61897781293842	-3.60988920432767
H	-4.18037237235848	6.32595303123157	-1.17264796880554
H	-5.66061459497316	6.58800847106843	-2.08871399710519
C	6.21662784706811	-2.74842022636396	4.62058888068044
C	5.33220782055352	-3.93985662304132	4.24695831904460
C	5.36997832881774	-1.54910723748446	5.05320455164573
C	4.43700945676589	-1.92608244198829	6.20702502436278
C	3.55051011606710	-3.11850397468322	5.83629397590573
C	4.39232094696260	-4.31880211871694	5.39395540247563
H	6.88079204987011	-3.03739795101249	5.44810426573335
H	6.86227372723590	-2.47469805635812	3.77803455832179
H	4.72707239626304	-3.67836707716599	3.36764876021258
H	5.95171021455117	-4.79865108893061	3.96141291764842
H	6.01461923100088	-0.70976009814922	5.34071435141646
H	4.76778675296973	-1.20503262595492	4.20012726149109
H	5.04210074681406	-2.18477274462371	7.08801193649035
H	3.82005910327156	-1.06426207259266	6.49152837653117
H	2.90877469511670	-3.39486161745653	6.68191415535917
H	2.88278897052763	-2.83411361637387	5.01185199926751
H	4.98618837592397	-4.68136671220801	6.24548044995991
H	3.73702085663000	-5.14292085341420	5.08651899354776
C	-4.62652365944444	6.08243354460610	2.06594754103170
C	-3.63530091399578	6.87506719965610	2.92108169326049
C	-5.41816031597778	5.08871350283594	2.91916042734249

C	-4.47680852890751	4.14623324037885	3.67353076430461
C	-3.48019247880494	4.93248726211779	4.52910105976368
C	-2.69388902029888	5.93824616740064	3.68268995006158
H	-5.30768829935664	6.76223575530012	1.53925159285767
H	-4.06995990647893	5.52857057718732	1.29667222614375
H	-3.05745647603211	7.56254369244882	2.29173342933404
H	-4.19218258410832	7.49469087508253	3.63895862481915
H	-6.03071145647732	5.64329282948392	3.64483809039404
H	-6.11087426410358	4.51561068722120	2.29310852431776
H	-5.05085460055736	3.45085401663078	4.29801146325681
H	-3.92237622919607	3.53498914953915	2.94720299321547
H	-4.02846515072050	5.47105318099462	5.31560265994618
H	-2.79400776970372	4.24252356447384	5.03611981330980
H	-2.01025256604995	6.51700504901724	4.31585325195613
H	-2.07259769481551	5.39275023758714	2.96045041855461
C	4.53015702646875	-6.50291999219891	-0.19316509410015
C	5.23797020786168	-6.46876437974386	-1.55018205552900
C	5.35123781525643	-5.78056355560119	0.87736729086580
C	5.65754073037225	-4.34241768322741	0.45247745799459
C	6.36163535573346	-4.29871967466748	-0.90595382897385
C	5.54629241404664	-5.03067644357243	-1.97533599849570
H	3.55699174141978	-5.99968677585748	-0.29193722537727
H	4.32641519595670	-7.53717116097652	0.11034065285236
H	6.17549990824996	-7.03882500741209	-1.47867289101699
H	4.62432599710198	-6.96700437199324	-2.31147246716239
H	4.81931988303667	-5.78696516439953	1.83683982615944
H	6.29413699479152	-6.32302831413881	1.03698439068170
H	4.71231871289015	-3.78936440502076	0.37042904177982
H	6.26463127827475	-3.84102608385761	1.21623078142517
H	6.52903956667505	-3.25795499090037	-1.21002123423672
H	7.35289641922369	-4.76603620404093	-0.81513041948126
H	4.60505963086265	-4.48923863606139	-2.13441802768090
H	6.08374971006779	-5.02475739977805	-2.93131663645175
